# Origin, structure, and composition of the spider major ampullate silk fiber revealed by genomics, proteomics, and single-cell and spatial transcriptomics

**DOI:** 10.1126/sciadv.adn0597

**Published:** 2024-08-14

**Authors:** Sumalata Sonavane, Sameer Hassan, Urmimala Chatterjee, Lucile Soler, Lena Holm, Annelie Mollbrink, Gabriele Greco, Noah Fereydouni, Olga Vinnere Pettersson, Ignas Bunikis, Allison Churcher, Henrik Lantz, Jan Johansson, Johan Reimegård, Anna Rising

**Affiliations:** ^1^ Department of Animal Biosciences, Swedish University of Agricultural Sciences, Uppsala, Sweden.; ^2^ Department of Biosciences and Nutrition, Karolinska Institutet, Neo, Huddinge, Sweden.; ^3^ National Bioinformatics Infrastructure Sweden (NBIS), Science for Life Laboratory (SciLifeLab), Uppsala University, Uppsala, Sweden.; ^4^ Department of Medical Biochemistry and Microbiology, Uppsala University, Uppsala, Sweden.; ^5^ Department of Gene Technology, KTH Royal Institute of Technology, SciLifeLab, Stockholm, Sweden.; ^6^ Department of Immunology, Genetics and Pathology, National Genomics Infrastructure, SciLifeLab, Uppsala, Sweden.; ^7^ Department of Molecular Biology, NBIS, SciLifeLab, Umeå University, Umeå, Sweden.

## Abstract

Spiders produce nature’s toughest fiber using renewable components at ambient temperatures and with water as solvent, making it highly interesting to replicate for the materials industry. Despite this, much remains to be understood about the bioprocessing and composition of spider silk fibers. Here, we identify 18 proteins that make up the spiders’ strongest silk type, the major ampullate fiber. Single-cell RNA sequencing and spatial transcriptomics revealed that the secretory epithelium of the gland harbors six cell types. These cell types are confined to three distinct glandular zones that produce specific combinations of silk proteins. Image analysis of histological sections showed that the secretions from the three zones do not mix, and proteomics analysis revealed that these secretions form layers in the final fiber. Using a multi-omics approach, we provide substantial advancements in the understanding of the structure and function of the major ampullate silk gland as well as of the architecture and composition of the fiber it produces.

## Teaser

Multi-omics expose spider silk’s intricate three-layered structure and unique protein makeup.

## INTRODUCTION

Spider major ampullate silk, distinguished by its exceptional mechanical properties, is a product of renewable resources and is synthesized within fractions of a second under ambient conditions (*1*). Its unique combination of rapid production, sustainability, and superior performance characteristics positions spider silk as a compelling environmentally friendly material for high-performance fiber applications (*2*). To unlock its potential, the development of artificial replicas of the pristine silk fiber is imperative, which necessitates a comprehensive understanding of the bioprocessing of the silk fiber.

**Fig. 1.**
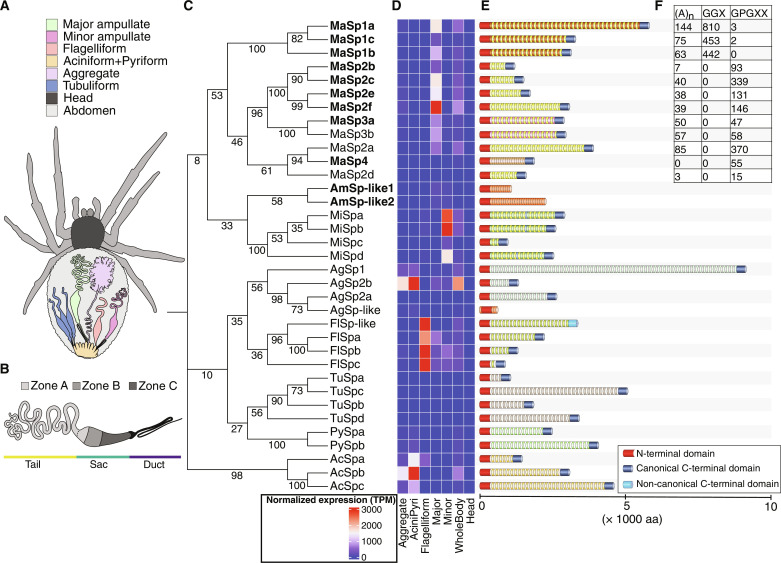
Spidroin catalog from *Larinioides sclopetarius*. (**A**) Schematic view of an orb-weaving spider with one set of each type of silk gland indicated. (**B**) Schematic figure of the major ampullate gland. The gland has three anatomical parts: the tail (yellow), the sac (green), and the duct (blue). The tail and sac are composed of a single-layered epithelium in which three morphologically distinct cell types are found, each localized to one of three zones [(A) to (C)], indicated as shades of gray. (**C**) Phylogenetic tree of the NT domain from the 35 spidroins identified in *L. sclopetarius*. Numbers on the branches indicate bootstrap values. Spidroins in bold were identified by proteomics analysis of the major ampullate gland and silk. (**D**) Heatmap with the expression of all spidroins in different tissues as determined by bulk RNA-seq. The colors correspond to transcripts per million shown in the scale (bottom left inset). (**E**) Schematic illustration of the spidroin genes. All spidroin genes encoded proteins with a signal peptide (not shown) and an NT domain (red block). Most spidroin genes encoded a canonical CT domain (dark blue block), except FlSp-like, which was found to have a noncanonical CT domain (light blue block), and AmSp-like1 and 2 and AgSp-like spidroins, which completely lacked the C-terminal nonrepetitive region. The repetitive motifs in each spidroin are represented as colored blocks. (**F**) Table showing the number of typical MaSp repeat motifs found in each of the MaSps. (A)_n_ refers to poly-alanine motifs; X in GGX and GPGXX represents any amino acid residue.

During the past 400 million years, spiders have refined their silk spinning abilities, and today, individual spiders of some species can spin seven different types of silks from specialized glands located in the abdomen (opisthosoma) (*3*) (Fig. 1A). These include major ampullate silk for making the radial threads and frame of the web, minor ampullate silk for making the inner spiral, flagelliform silk for making the extensible capture spiral that is coated by sticky droplets of aggregate silk, pyriform silk for making attachment discs, tubuliform silk for making the outer layer of the egg sac, and aciniform silk for making the inner part of the egg sac and/or swathing prey (*4*). The different silk types are produced in separate sets of glands, which have secretory ducts connected to spigots located caudoventrally in the abdomen (*5*). The spider silks are primarily composed of spider silk proteins (spidroins), characterized by a distinctive architectural arrangement that features nonrepetitive N- and C-terminal domains flanking an extensive repetitive region (*6*–*13*). The terminal domains, herein referred to as NT and CT, respectively, are evolutionarily conserved (*14*), while the nature of the repetitive regions differs between the different spidroin types (*7*–*11*).

The major ampullate silk is the strongest of the different silks, and the main components of this fiber are called major ampullate spidroins (MaSps) (*7*–*13*). The repetitive regions of the MaSps are rich in glycine and alanine that organize into typical motifs, which, together with clustering of the terminal domains in phylogenetic analyses, have led to the classification of MaSps into five types (MaSp1 to MaSp5). MaSp1 (*15*) typically has poly-Ala blocks interspersed with Gly-rich repeats. MaSp2 (*16*) repeats are rich in proline and glutamine with abundant Gly-Pro-Gly and Gln-Gln motifs that MaSp1 lacks. MaSp3 (*17*) is similar to MaSp1 but is void of the Gly-Gly motifs commonly found in MaSp1. In contrast, MaSp4 and MaSp5 (*18*) repeats lack poly-Ala blocks, but MaSp4 contains Gly-Pro-Gly motifs common to MaSp2 whereas MaSp5 has Gly-Gly motifs like MaSp1. Evidently, the major ampullate fiber is a composite of different MaSps, which has been suggested to be related to the supreme mechanical properties of the fiber (*19*). For example, MaSp1 likely contributes primarily to fiber strength, while MaSp2 is more related to the fiber strain (*12*, *20*), and expression of MaSp3 and MaSp4 has been suggested to be correlated to fiber toughness (*12*, *18*). Although recent work has extended our understanding of the spider genomes (*7*–*11*, *13*, *21*), the spidroin genes are difficult to assemble because they contain extended stretches of repeats and are often found in tandem arrays in the genome. Therefore, it appears that previous descriptions of spidroin gene repertoires are incomplete.

In addition to MaSps, the major ampullate silk contains several proteins that do not conform to the overall architecture of a classical spidroin. These proteins form a heterogeneous group termed spider silk-constituting elements (SpiCE) that differ in molecular weight and amino acid composition (*8*–*10*). A subgroup within this group of proteins, known as cysteine-rich proteins (CRPs), stands out for its high cysteine content (*22*), and is suggested to have a structural role in the fiber, possibly by forming large disulfide-linked complexes (*23*).

The major ampullate silk is produced in paired glands, each consisting of a long tubular tail, a wide sac, and a narrow s-shaped duct (*24*) (Fig. 1B). The lumen of the duct is lined by a cuticular intima, a hard and chitin-rich structure believed to aid in the removal of water during the spinning process and to protect against tissue laceration during spinning (*24*, *25*). Histological studies of the gland of distantly related spider species have shown that the tail and sac are composed of three types of columnar epithelial cells confined to three different zones (A, B and C) (*26*). Zone A cells are found in the tail and proximal parts of the sac (toward the tail), the middle part of the sac harbors zone B cells, while the most distal part of the sac next to the duct is composed of zone C cells (Fig. 1B). According to immuno-transmission electron microscopy studies performed using an antibody that identified the conserved spidroin NT domain, zone A and B cells produce spidroins, while the nature of the secretion produced by zone C cells remains uncharacterized (*26*). Investigations of the major ampullate silk have shown that the fiber has a core-skin structure (*26*–*33*), wherein the central core contains the spidroins (*32*, *34*) while the surrounding skin layer is composed of unknown proteins and other components like carbohydrates, glycoproteins, and lipids (*30*, *32*, *33*). Consequently, the compositions of the secretions from zones A, B, and C are largely unknown, and the link between the epithelial zones and the architecture of the fiber remains to be revealed.

In the gland, the spidroins are stored as a soluble and viscous dope at high concentrations (~50% w/v) (*35*) and at around neutral pH (*36*). During storage, the MaSps are dimeric, interconnected in the CT domain (*37*). As the dope travels from the sac and through the duct, the pH is lowered from ca. 6.3 to <5.7, which induces antiparallel dimerization of the NT domain (*36*, *38*, *39*). Because the MaSps are already interconnected via their CT domains (*36*, *37*), the dimerization of NT leads to the interlocking of the spidroins into large networks (*40*). At the same time, shear forces acting on the dope in the narrowing duct combined with the increasingly acidic environment destabilize the CT domain, which transitions into β-sheet conformation (*36*, *37*). When the fiber is pulled out from the spider (*41*), the pulling forces will be propagated along the protein chains (because they are linked in macromolecular networks), which aids in the alignment and conversion of the poly-Ala blocks in the repetitive region into β-sheet crystals (*1*, *40*).

Here, we present a complete spidroin repertoire from an orb-weaving spider, *Larinioides sclopetarius*. Spatial transcriptomics and single-cell RNA (scRNA) sequencing (scRNA-seq) showed that the tail and the sac of the major ampullate gland are composed of six silk-producing cell types found in the three distinct zones (A, B, and C). This, combined with image analyses of histological sections and proteomics analyses of sequentially dissolved fibers, indicate that the silk fiber is a three-layered structure in which the core is composed of proteins belonging to the MaSp1, MaSp2, and MaSp4 families, the middle layer is dominated by MaSp3, and the outermost layer contains several uncharacterized nonspidroin proteins. Thus, we present a detailed description of the structure and composition of the major ampullate silk gland and fiber, in which we successfully link the layered epithelial secretions in the gland to those in the silk fiber. Our results bridge the long-standing knowledge gaps necessary for the production of biomimetic artificial silk fibers.

## RESULTS

### The *L. sclopetarius* major ampullate fiber is composed of 18 silk proteins

**Fig. 2.**
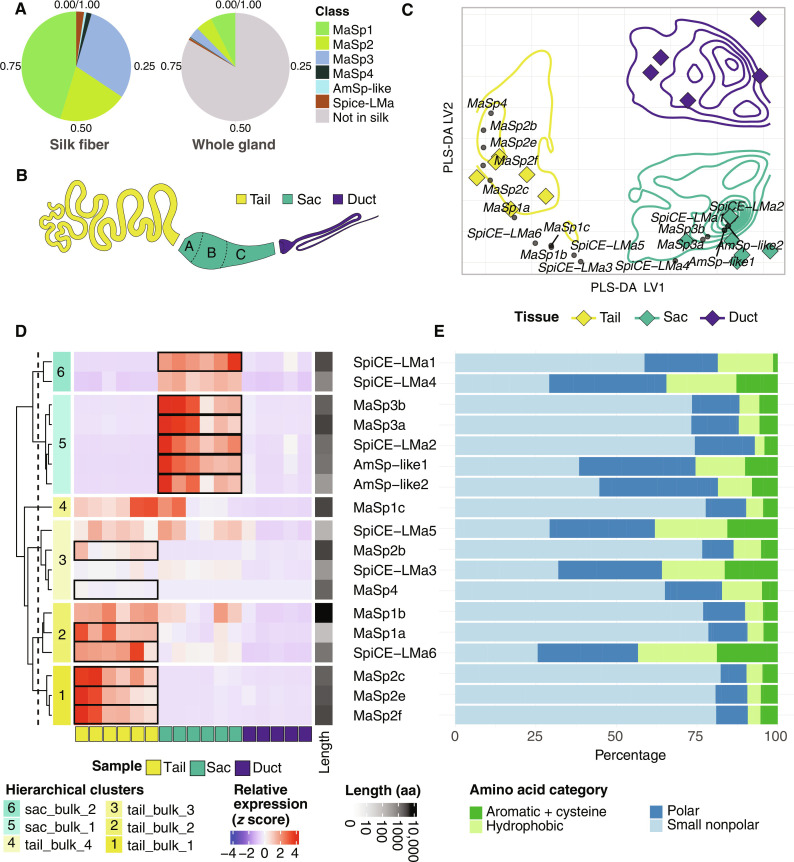
Expression of the 18 silk genes in the major ampullate tail, sac, and duct. (**A**) Relative quantification of the peptides identified in the major ampullate gland and in silk fibers, dissolved in 2 to 8 M urea, using LC-MS/MS proteomics, colored according to protein classes (MaSp1, MaSp2, MaSp3, MaSp4, AmSp-like, and SpiCE-LMa). (**B**) Schematic figure of the gland showing the three parts (tail, sac, and duct) that were separated for RNA-seq experiments. (**C**) PLS-DA of the bulk RNA data separates the three different parts (tail, sac, and duct). The *x* and *y* axes represent the values or the loading of genes on the first and second latent variables, LV1 and LV2, respectively. Colors indicate different sample types (tail/sac/duct) as shown in (B). Diamonds represent samples and small circles represent the overlay of the 18 silk genes. (**D**) Heatmap showing the relative expression levels (*z* score) for the 18 silk genes in the tail, sac, and duct samples (indicated as yellow, green, and blue, respectively, in the *x* axis). Gene names are shown on the right. Hierarchical clustering separates the genes into six subclusters based on their expression profiles shown as a dendrogram on the *y* axis. Black boxes indicate up-regulated differentially expressed genes (*P* < 0.05). The six subclusters identified are colored as shades of yellow or green based on whether the highest gene expressions were in the tail or sac parts, respectively. The bar on the right indicates protein length. The scale ranges from 0 (white) to 10,000 amino acid residues (black). (**E**) Percentage of different categories of amino acid residues in each of the 18 silk proteins (%). The colors indicate different categories (small nonpolar: A, G, P, S, and T; hydrophobic: I, L, M, and V; polar: D, E, H, K, N, Q, and R; aromatic and cysteine: C, F, W, and Y).

Here, we focus on the Swedish bridge spider (*L. sclopetarius*) that produces major ampullate silk fibers with impressive mechanical properties (fig. S1). First, the genome of this spider species was sequenced, assembled, and annotated (Table 1, Supplementary Notes, tables S1 to S11, and fig. S2). Manual curation resulted in a catalog of 35 complete spidroin genes (Fig. 1 and figs. S3 to S10). Next, to reveal the protein composition of the major ampullate gland and silk fiber, respectively, proteomics analysis was performed using liquid chromatography–tandem mass spectrometry (LC-MS/MS) (table S1). In the gland samples, 3985 proteins were identified in at least one of the three replicates (table S12). Dissolving the silk fiber can be challenging and different chemical treatments can extract different proteins (*22*). Therefore, three solvents, urea, hexafluoroisopropanol (HFIP), and lithium bromide (LiBr), were used to extract proteins from the fibers. A total of 1835 proteins were identified (table S12), of which 325 were identified in all three biological replicates from at least one treatment. However, because the fibers are easily contaminated by other silk types during spinning and by unrelated proteins during handling, only the proteins that were also found in the major ampullate gland proteomic data were predicted to have a signal peptide, and spectral counts exceeding 0.1% of the total spectra in LC-MS/MS analysis were considered (Fig. 2A). This process rendered 18 silk proteins that were named “the 18 silk proteins” (Table 2 and table S13) and corresponding genes were named “the 18 silk genes” (Table 2). The 18 silk proteins included 10 MaSps, two ampullate spidroin-like proteins (AmSp-like1 and AmSp-like2) and six proteins with unknown functions (table S13). The six proteins of unknown function were annotated as spider silk-constituting elements (*8*–*10*) of *Larinioides* major ampullate silk (SpiCE-LMa1 to SpiCE-LMa6) in order of abundance. LC-MS/MS analysis of fibers that were completely dissolved showed that peptides mapping to MaSp1 (a to c), MaSp2 (b, c, e, and f), MaSp3 (a and b), and MaSp4 represented 46%, 20%, 30%, and 1%, respectively, when considering percentage of total spectra (Fig. 2A). Peptides mapping to SpiCE-LMa proteins accounted for 2% while the AmSp-like proteins only constituted 1% (Fig. 2A). It should be noted that the large difference in molecular weight between the 18 proteins and the extreme repetitiveness and sequence similarity of the MaSps are problematic when quantifying the protein composition of these samples. We therefore analyzed the data with three additional methods: (i) intensity-based absolute quantification (iBAQ), (ii) normalized spectral abundance factor (NSAF), and (iii) label-free quantification (LFQ) intensity. The estimates of the composition of the fiber obtained from these four methods slightly differ (fig. S16). However, the order of abundance of MaSps and SpiCE-LMa proteins was similar across the methods; i.e., MaSp1, MaSp2, and MaSp3 were always the three most abundant classes of proteins and the SpiCE-LMa proteins only contributed a small fraction. The methods based on intensity (iBAQ and LFQ) suggested that MaSp2 proteins were the most abundant, whereas the MaSp1 proteins were the most abundant when using the methods based on spectral counts (total spectra and NSAF). Consequently, the intra-sample protein composition estimations reported herein, which are derived from label-free LC-MS/MS analyses, should be interpreted with caution. Comparisons across samples (see below), on the other hand, are more reliable because technical biases are the same.

**Table 1.** Assembly and annotation statistics of the *Larinioides sclopetarius* genome.

Genome assembly	
Assembly size (bp)	2,274,070,471
GC %	30.5
Number of contigs	1602
Longest contig (bp)	29,089,857
N50 (bp)	5,487,611
N90 (bp)	1,366,626
BUSCO complete (%)	97.3
	
**Repeat statistics**	
Number of elements	2,761,064
Length (bp) [% genome]	47.50%
	
**Genome annotations**	
Protein-coding genes	22,860
Transcript isoforms	46,911
BUSCO complete (%)	96.8

**Table 2.** Ranking of the 18 silk proteins/genes as markers in the different methods used.

	Differential gene expression					
	Bulk*		Spatial†		Single cell‡	
Gene	Part	Rank	Zone	Rank	Cell type	Rank
*MaSp2b*	Tail	19	Zone A	31	ZoneA_MaSp2	3
*MaSp2c*	Tail	9	Zone A	3	ZoneA_MaSp2	2
*MaSp2e*	Tail	6	Zone A	6	ZoneA_MaSp2	4
*MaSp2f*	Tail	1	Zone A	5	ZoneA_MaSp2	1
*MaSp4*	Tail	34			ZoneA_MaSp2	12
*MaSp1a*	Tail	38	Zone A	1	ZoneA_MaSp1	1
					ZoneA_MaSp2	13
*MaSp1b*			Zone A	4	ZoneA_MaSp1	2
*MaSp1c*			Zone A	32	ZoneA_MaSp1	3
*SpiCE-LMa6*			Zone A	12	ZoneA_SpiCE-LMa	6
*SpiCE-LMa3*					ZoneA_SpiCE-LMa	28
*SpiCE-LMa5*					ZoneA_SpiCE-LMa	11
*MaSp3a*	Sac	19	Zone B	2	ZoneB_MaSp3	5
*MaSp3b*	Sac	18	Zone B	3	ZoneB_MaSp3	1
*AmSp-like2*	Sac	42	Zone B	4	ZoneB_MaSp3	8
			Zone C	61		
*AmSp-like1*	Sac	49	Zone B	9	ZoneB_MaSp3	12
*SpiCE-LMa4*	Sac	39	Zone B	72	ZoneB_MaSp3	68
					ZoneC_SpiCE-LMa	85
*SpiCE-LMa2*	Sac	63	Zone B	23	ZoneB_MaSp3	6
			Zone C	16	ZoneC_SpiCE-LMa	11
*SpiCE-LMa1*	Sac	68	Zone C	13	ZoneC_SpiCE-LMa	1

*DEG analysis on the tail, sac, and duct parts of the major ampullate glands. Rank is based on average log_2_-fold change. The higher the rank, the more differentially expressed the gene is.

^†^Marker gene identification on spots manually annotated as different zones within the spatial transcriptomic sections. Rank is based on average log_2_-fold change; the higher, the better.

^‡^Marker gene identification in cell types identified with single-cell data. Rank is based on average log_2_-fold change; the higher, the better.

The six SpiCE-LMa proteins generally had a lower molecular weight compared to the MaSps (table S13). In terms of amino acid composition, SpiCE-LMa1 and SpiCE-LMa2 resembled the MaSp proteins and SpiCE-LMa2 had spidroin-like repeat motifs. SpiCE-LMa3 to SpiCE-LMa6 were rich in Cys and resembled the amino acid composition of AmSp-like1 and AmSp-like2 (Fig. 2E and figs. S17 to S19). The predicted secondary structure content and AlphaFold2 structural predictions (*42*, *43*) of the SpiCE-LMa proteins suggested large heterogeneity within this group of proteins (figs. S20 to S26).

### The 18 silk genes are expressed in the tail and the sac

Bulk RNA sequencing (RNA-seq) of whole major ampullate glands was used to verify that the RNA levels correlated with the protein abundance in the gland (fig. S27A). Notably, the 18 silk genes ranked among the most highly expressed genes (fig. S28A). However, this analysis did not permit localization of the expression of specific genes to different parts of the gland. To enhance the resolution of the gene expression profiles, the major ampullate gland was sequenced after being cut into three distinct anatomical parts: tail, sac, and duct (Fig. 2B). This implies that the tail samples contained transcripts from zone A while the sac samples contained transcripts from all three zones (Figs. 1B and 2B and table S1). The major ampullate gene set (table S14) was identified by principal components analysis (PCA) of the enriched genes in the gland (fig. S28). This gene set was then used to perform partial least square discriminant analysis (PLS-DA) regression on the tail, sac, and duct samples. This analysis separated the three types of samples into distinct clusters using only two latent variables with high predictive relevance (*Q^2^* = 0.857), verifying that the transcriptome in the tail, sac, and duct are diverse (Fig. 2C). To identify which part of the major ampullate gland the 18 silk proteins were originating from, the 18 silk genes were superimposed on the PLS-DA plot, which revealed that all the MaSp genes were expressed in the major ampullate tail, except *MaSp3a* and *MaSp3b*, which were expressed in the sac along with AmSp-like1 and AmSp-like2 (Fig. 2C). Three genes encoding proteins of unknown function, *SpiCE-LMa1*, *SpiCE-LMa2*, and *SpiCE-LMa4*, were also among the genes expressed in the sac. The expression profiles of the silk genes in the three parts (tail/sac/duct) were further visualized by a heatmap (Fig. 2D), which clearly indicated that the genes encoding the 18 silk proteins are expressed in the tail and the sac but not in the duct.

Hierarchical clustering grouped the 18 silk genes into six clusters based on the similarity in their expression profiles (Fig. 2D and fig. S27B). Of these, cluster 1 genes showed differential expression (DE) in the tail samples and were assigned to the tail, while all the genes in clusters 5 and 6 showed DE in sac samples and were assigned to the sac. Clusters 2, 3, and 4 had some genes that were differentially expressed in the tail and clustered in the same node as cluster 1 and, therefore, were assigned to the tail. The tail clusters are colored as shades of yellow while sac clusters are shown as shades of green. On the basis of these results, cluster 1 that contained *MaSp2*, *c*, *e*, and *f* genes, which were solely expressed in the tail, could be assigned to zone A (Figs. 1B and 2D), but the rest of the genes, which were also expressed in the sac, could not be assigned to one of the zones because the sac samples contained tissue from all the three zones.

### Expression of the silk genes is spatially resolved in the three zones

**Fig. 3.**
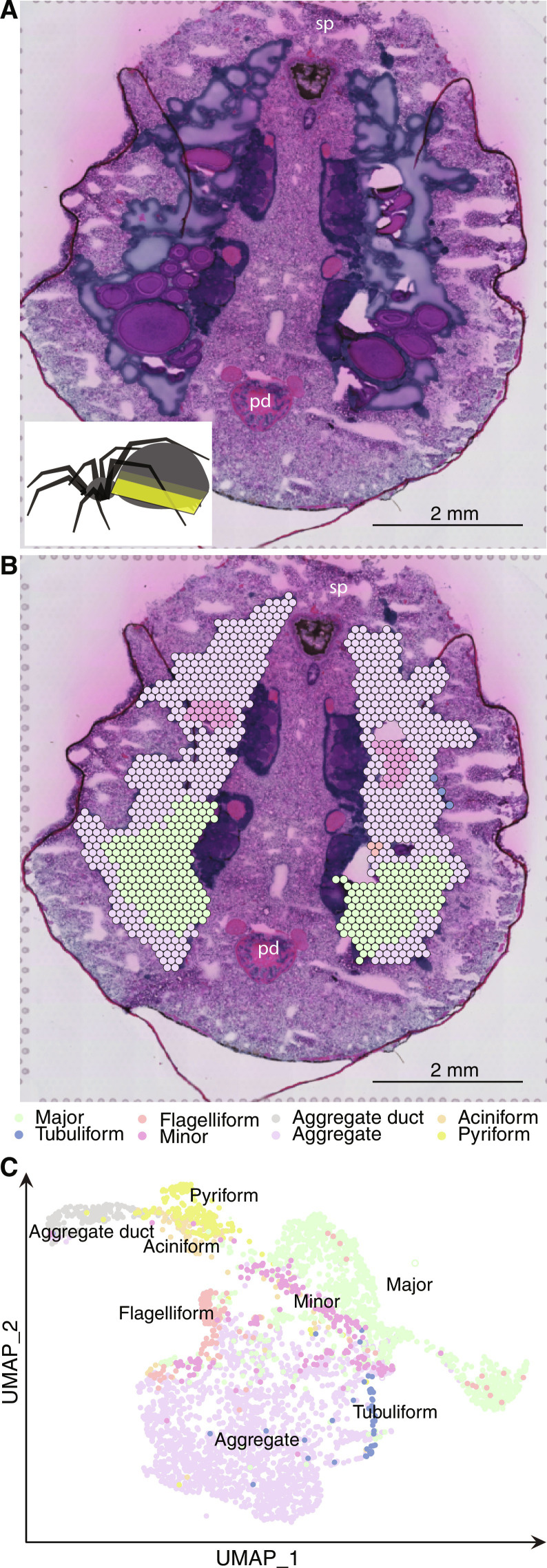
Spatial transcriptomics of silk glands. (**A**) H&E-stained section of the spider abdomen. The inset shows a top view of a lateral section of the abdomen with the approximate plane where the section was made. (**B**) The spots were annotated as different silk glands based on the morphology of the tissue and the spatial location. In (A) and (B), sp and pd indicate the location of the spinnerets and the pedicel, respectively; scale bar, 2 mm. (**C**) UMAP analysis of all spots, from eight sections, that were manually annotated as silk glands. The axes (UMAP-1 and UMAP-2) represent the first and second UMAP dimensions, respectively. Each dot represents a spot in the spatial sections.

To improve the resolution of the silk gene expression, we next used spatial transcriptomics (10X Visium). This is an unbiased and elegant technique that allows mapping of the gene expression in tissue sections with a resolution of 50 μm (*44*) (Supplementary Notes). Six sections of the whole abdomen from four female individuals were used and the spots on the hematoxylin and eosin (H&E)–stained sections were manually annotated to different silk glands based on histology (one section is shown in Fig. 3, A and B; figs. S12 and S13 show additional sections). The sequencing data from all spots from all the sections were then isolated and visualized using Uniform Manifold Approximation and Projection (UMAP) (*45*). In UMAP, the spots assigned as silk glands clustered together and separated from the spots belonging to other tissue types (fig. S14). Within the silk gland cluster, the spots from different silk glands clustered together, in line with the manual annotation (Fig. 3C). On the spatial sections, the spidroin expression was specific to corresponding glands (fig. S15) and confirmed the results from the bulk-RNA expression profiles (Fig. 1D), attesting to the quality of the spatial data.

**Fig. 4.**
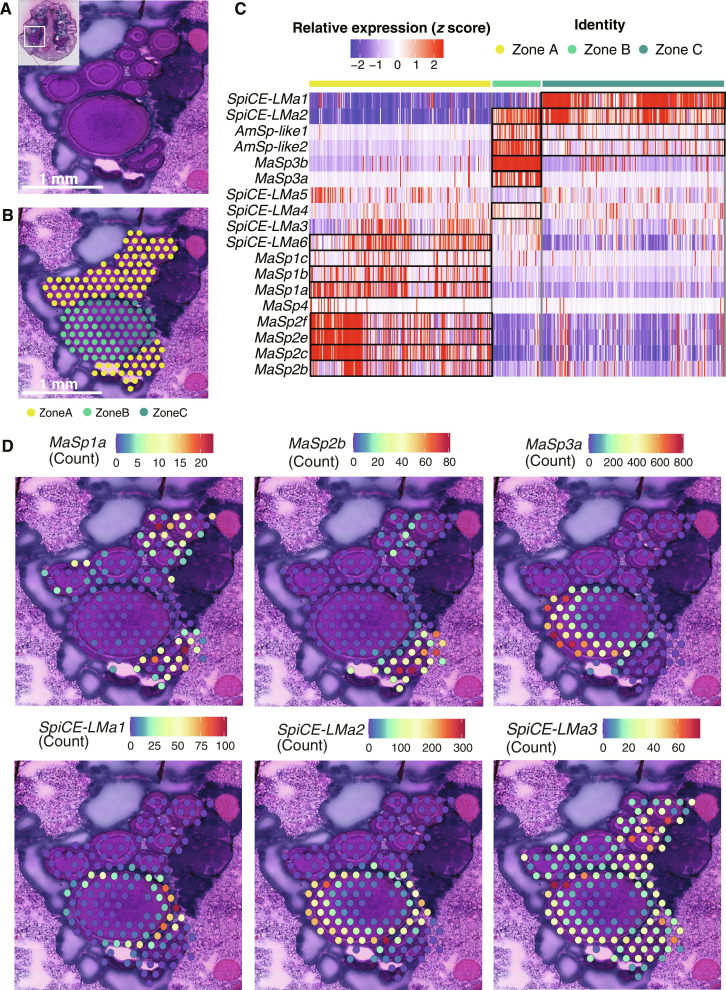
Spatial resolution of silk protein expression in zones A, B and C. (**A**) One of the major ampullate glands in section 1 contains several cross sections of the tail and a cross-sectioned sac (H&E staining). The inset shows the original image from which the region was magnified (white square). (**B**) The spots corresponding to the major ampullate gland were annotated as zone A, B, or C based on the morphology and staining of the epithelium overlaying each spot. In (A) and (B), scale bar, 1 mm. (**C**) Heatmap showing the expression of the 18 silk genes in the 847 spots annotated as zones A, B, and C, respectively. Each bar on the heatmap represents a spot on the spatial section and black boxes indicate marker genes (*P* < 0.05) in different zones. (**D**) Expression profiles of *MaSp1a*, *MaSp2b*, *MaSp3a*, *SpiCE-LMa1*, *SpiCE-LMa2*, and *SpiCE-LMa3* (in order) in the three zones of the major ampullate gland shown in (A) and (B).

The spots annotated as covering major ampullate gland tissue could be further assigned as zone A, B, or C based on the morphology of the epithelial cells in six of the sections (Fig. 4, A and B, and high-resolution H&E-stained image of zones in fig. S29A). This resulted in 847 spots from six sections that clustered according to zone in UMAP (fig. S29B), indicating that the expression profiles in these zones are indeed different. Furthermore, marker genes for zones A, B, and C identified from the spatial data overlapped with the bulk-RNA PLS-DA plot of the tail and sac but not the duct (fig. S30A). The expression of the 18 silk genes on all the spots annotated as zone A, B, or C was then visualized as a heatmap (Fig. 4C). Statistical analyses of the expression levels revealed that 15 of the 18 silk genes were predominantly expressed in at least one of the zones (Table 2). *MaSp1 (a–c)*, *MaSp2 (b, c, e, f)*, and *SpiCE-LMa6* genes had significantly higher expression in zone A cells; *MaSp3 (a, b)*, *AmSp-like1*, and *SpiCE-LMa4* in zone B cells; and *SpiCE-LMa1* in zone C cells. The expression of *SpiCE-LMa2* and *AmSp-like2* was significantly higher in both zone B and zone C. The expression of the remaining three genes, *MaSp4*, *SpiCE-LMa3*, and *SpiCE-LMa5*, did not differ significantly between the zones (Fig. 4C). The expression profiles of selected genes in one of the major ampullate glands in section 1 are shown as examples in Fig. 4D. Expression profiles of the 18 silk genes in all other spatial sections can be found in figs. S31 to S35.

### The 18 silk genes in the three zones are specifically expressed in six cell types

**Fig. 5.**
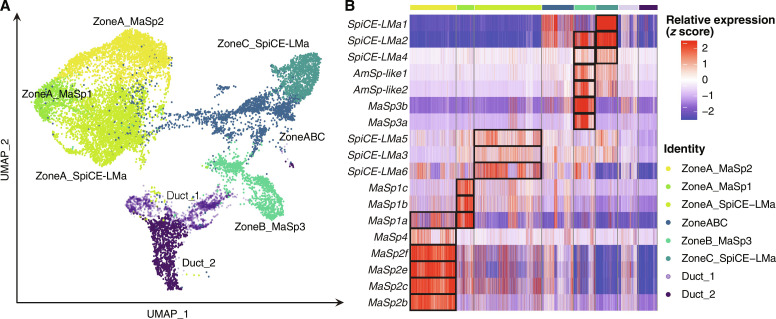
scRNA-seq analysis of major ampullate gland reveals eight cell types. (**A**) Eight cell types in the major ampullate gland visualized in UMAP. The axes (UMAP_1 and UMAP_2) represent the first and second UMAP dimensions, respectively. Each dot represents a cell. The cell types were annotated by correlating the gene expression with the bulk RNA and spatial transcriptomics data. This revealed that six cell types make up the secretory epithelium of the tail and sac; three cell types were confined to zone A, one to zone B, one to zone C, and one could be found in all three zones. Two cell types were found in the duct. (**B**) Heatmap of relative gene expression (*z* score) of the 18 silk proteins in different single-cell types. Black boxes indicate the marker genes (*P* < 0.05) in different cell types.

The *MaSp1* and *MaSp2* genes were both expressed in the zone A cells, but the spatial transcriptomics analyses revealed that their expression profiles differ in different regions of zone A (Fig. 4 and figs. S31 to S35). This suggests that the epithelial zones could harbor several different cell types. To elucidate this, scRNA-seq was performed on whole major ampullate glands isolated from seven individuals (table S1). After quality control (QC), filtering, and analysis, 9700 cells were obtained, which clustered into eight groups. On the basis of the gene overlap with the first two components of PLS-DA of the bulk RNA gene sets, these eight clusters were found to originate from either tail, sac, or duct of the gland (fig. S30B). Three of the cell clusters were specific for the tail, three for the sac, and two for the duct. The clusters were then compared with the spatial transcriptomics data, which allowed them to be classified as zone A/B/C cells based on their gene overlap with the marker genes of the three different zones (fig. S30C). Three clusters were identified in zone A, one in zone B, one in zone C, and one cluster was found in all three zones. The clusters were assigned as cell types and named according to the zone and top marker spider silk protein gene (Fig. 5A and table S15). One of the zone A cell types had *MaSp1a–c* as the top three marker genes and was named ZoneA_MaSp1. The second zone A cell type had *MaSp2b*, *c*, and *f* as the top three marker genes and was therefore annotated as ZoneA_MaSp2. This cell type included *MaSp4* as one of the top marker genes. In the last zone A cell type, *SpiCE-LMa3*, *SpiCE-LMa5*, and *SpiCE-LMa6* were the top marker genes among silk genes and the cell type was named ZoneA_SpiCE-LMa. In zone B cells, the top marker genes were *MaSp3a* and *MaSp3b* and the cell type was named ZoneB_MaSp3 cells. These cells also had *AmSp-like1* and *AmSp-like2*, two of the spidroins lacking the CT domain (Fig. 1E), and *SpiCE-LMa2* and *SpiCE-LMa4* as top marker genes. The zone C cells had *SpiCE-LMa1*, *SpiCE-LMa2*, and *SpiCE-LMa4* as the top marker genes among the silk genes and hence named ZoneC_SpiCE-LMa cells. The final cell type was mainly found in the sac but could not be assigned to a specific zone and was therefore named ZoneABC (fig. S30). The remaining two cell types were named according to their similarity in expression profile to the bulk-RNA data from the duct as Duct_1 and Duct_2 (fig. S30B), and these two cell types did not have any of the silk proteins as marker genes (Fig. 5B).

A compelling observation is that all the 18 silk genes were among the top 100 marker genes in at least one of the six cell types when considering all the 22,860 protein-coding genes. In most cases, they were among the top 10 (Table 2 and Fig. 5B). All the cell types express a distinct set of silk genes as evidenced by the fact that 15 of 18 silk genes were significantly expressed in only one of the six cell types, while the remaining three genes were expressed in two cell types (Table 2). In summary, the presence of the 18 silk proteins in the fiber can be directly related to the expression of the corresponding genes in the six cell types.

### The six silk-producing cell types can be spatially resolved along the gland

**Fig. 6.**
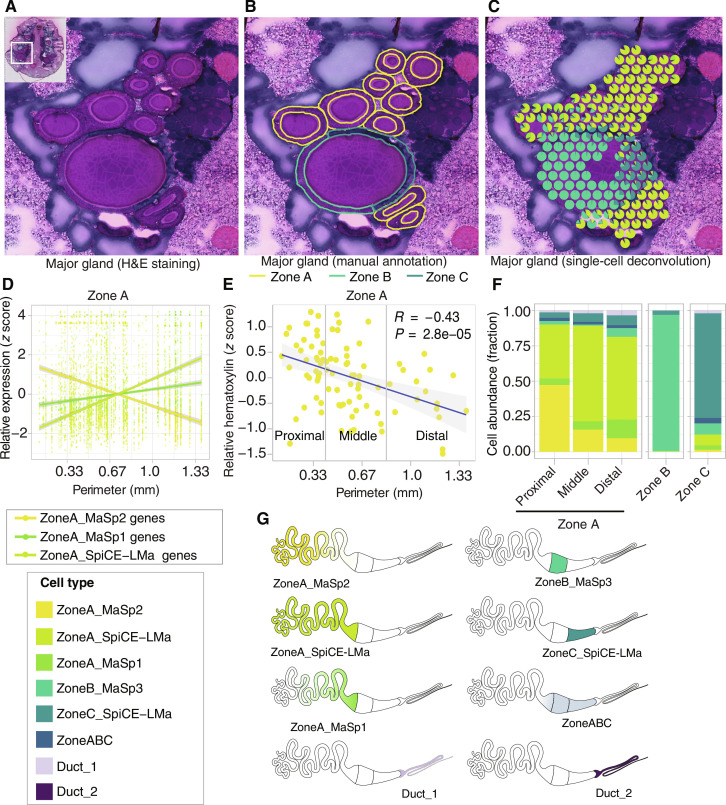
Spatial distribution of the eight major ampullate cell types. (**A**) H&E-stained section of the spider major ampullate gland; the inset shows the area magnified. (**B**) The same section as in (A) with the zones (A in yellow, B in green, and C in dark green) of the major ampullate gland indicated. The zones were identified based on the cell morphology, and 11 cross sections were obtained for this gland (9 for zone A, 1 for zone B, and 1 for zone C). (**C**) Deconvolution of the spatial spots using the scRNA-seq data generates a pie chart for each spot, where the colors represent cell types identified from the scRNA-seq data, and shows the fraction of cells that belongs to each cell type. (**D**) Relative gene expression of the silk protein genes that are marker genes for the cell types in zone A as a function of the perimeter of the gland cross sections. The colors correspond to the three zone A cell types and the lines are the linear models of the gene expression profiles for the respective cell types. (**E**) Pearson correlation (*R* = −0.43, *P* = 2.8 × 10^−5^, *N* = 80) of hematoxylin mean values to the perimeter of the major ampullate gland cross sections in zone A. (**F**) Average fraction of cell types identified from scRNA-seq data in zone A (proximal, middle, and distal), zone B, and zone C spots as evaluated from the deconvolution plots. (**G**) Schematic figures showing the spatial distribution of the eight cell types in the major ampullate glands.

To identify the spatial location of the cell types in the major ampullate gland, the scRNA-seq data were combined with spatial transcriptomics data, and deconvolution of the two datasets was performed. This allowed us to visualize the spatial distribution of cell types in the spots annotated as major ampullate glands on the spatial sections (Fig. 6, A to C, and figs. S36 and S37). The pattern that emerged suggested that the cell types confined to zone A may not be evenly distributed along this zone. To address this, we took advantage of the tapering nature of the gland’s tail to order the cross sections from proximal (small perimeter) to distal (large perimeter) and used image analysis using QuPath (*46*). The sectioned major ampullate glands were annotated according to zones, as shown in Fig. 6B, resulting in 101 cross sections for zone A, 2 for zone B, 9 for zone C, and 5 for the duct across all sections. This annotation was confirmed by digital color deconvolution in QuPath, which generates values for the degree of H&E staining, respectively, for each area of interest (fig. S38). Furthermore, the perimeters of all the cross-sectioned parts of the gland were determined using QuPath and associated with the spots on the spatial transcriptomic sections. The spatial spots corresponding to each cross section in zone A were extracted, and the mean expression of marker genes in these spots as a function of the perimeter of the cross section was visualized (Fig. 6D and fig. S39). This revealed higher *MaSp2* expression in zone A cross sections with smaller perimeters (proximal tail parts), while *MaSp1* and *SpiCE-LMa3* genes exhibited higher expression in zone A cross sections with larger perimeters (distal tail, toward the sac). Notably, a significant negative correlation was observed between hematoxylin values and the cross-sectional perimeter in zone A, further supporting the presence of different cell types along this zone (Fig. 6E). Next, based on the perimeter values, the zone A cross sections were split into three parts: proximal, middle, and distal. The distribution of cell types in the spots corresponding to different parts of zone A was obtained by combining the scRNA-seq, the spatial, and the QuPath data and visualized in Fig. 6F. ZoneA_MaSp2 cells showed higher abundance in proximal zone A regions, which gradually decreased in middle and distal parts. Conversely, ZoneA_MaSp1 cells were more prevalent in distal parts compared to the most proximal region. ZoneA_SpiCE-LMa cells were found along the whole length of zone A, but most frequently in the middle and distal parts. ZoneB_MaSp3 cells were almost exclusively found in spots annotated as zone B, affirming their identification as zone B cells. Zone C spots were dominated by ZoneC_SpiCE-LMa cells, with a minor fraction of other cell types. ZoneABC cells were found in all the zones with low abundance. The distribution of the different cell types along the major ampullate gland is illustrated in Fig. 6G.

### The location of the silk-producing cell types determine the composition of layers in the silk fiber

**Fig. 7.**
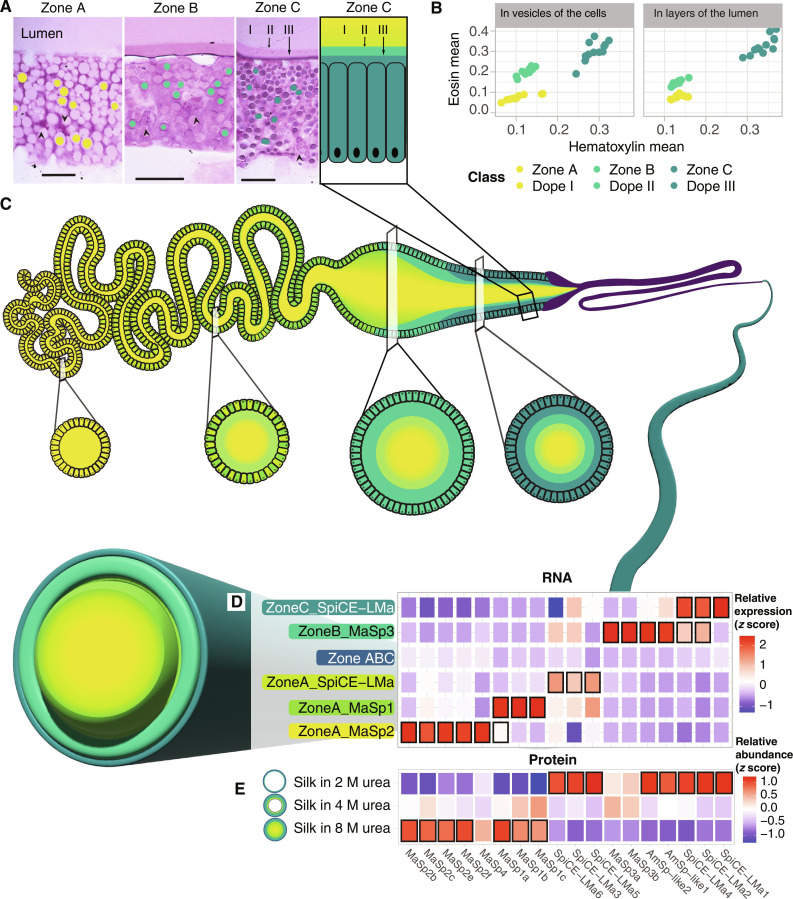
Origin and model of the multi-layered architecture of the major ampullate silk. (**A**) Histological sections of the *L. sclopetarius* major ampullate gland zones A, B and C, respectively (H&E staining). Secretions from zones A, B, and C form three layers in the lumen (indicated as I, II, and III in the zone C panels). Filled circles around the intracellular vesicles in the first three panels indicate the annotated regions for image analysis in QuPath. Scale bar, 20 μm. (**B**) H&E intensity plots of the annotation objects shown in (A). (**C**) Schematic image of the major ampullate gland and model of the multi-layered architecture of the major ampullate silk. The localization of the six cell types and the layered secretions are illustrated in the gland schematic. Below the gland, schematic drawings of cross sections from four locations along the gland are shown. In the model of the major ampullate silk, the colors correspond to the cell types the layers originate from, and the thickness of each layer is based on the proteomics analysis of the fiber (% total spectra). It should be noted that the histological sections suggest that the middle and outer layers could be thinner than indicated in the schematic figure of the fiber. (**D**) Heatmap showing gene expression values of the 18 silk proteins in the six cell types that are found in zones A, B and C. (**E**) Heatmap showing the peptides mapping to each of the 18 silk proteins in soluble extracts from major ampullate fibers incubated in 2, 4, and 8 M urea, respectively. In (D) and (E), colors indicate expression values (*z* scores), and the black frames indicate genes/proteins that were identified as significantly enriched (*P* < 0.05 when comparing samples between 2 and 8 M urea). The 18 silk genes/proteins are listed below the panels.

In line with previous reports for other spider species (*26*), the secretions originating from zones A to C within *L. sclopetarius* major ampullate glands stained distinctly with H&E and were separated in the gland lumen (Fig. 7A). To confirm the zone-specific origin of these secretions, the vesicles in the cells of each zone and the secreted substances forming layers in the lumen were annotated and evaluated for staining intensity using QuPath image analysis. The obtained H&E intensity values for the vesicle content and the secreted substances within the lumen were plotted and color-coded according to their respective zones. The data obtained for vesicle content and secretions from the respective zones formed clusters, clearly indicating that the zone A secretion formed the bulk of the silk feedstock in the sac lumen, the secretion from zone B contributed a surrounding middle layer, while the secretion from zone C formed the outermost thin layer (Fig. 7B and fig. S40).

To verify that the three layers in the liquid silk feedstock persist in the major ampullate silk fiber, a solubilization protocol involving the use of different concentrations of urea (2, 4, and 8 M) was used (*28*). Low concentrations of urea (2 M) will not solubilize the entire silk and, therefore, enrich for proteins present at the surface, while the highest concentrations (8 M) will dissolve the whole fiber. LC-MS/MS analysis of the solubilized fractions provided the relative fraction for each protein in the different urea concentrations. The SpiCE-LMa1 to SpiCE-LMa6 and AmSp-like1 and 2 proteins were significantly enriched in 2 M urea supernatants compared to the samples dissolved in 8 M urea (*P* < 0.05), while the MaSp1a to MaSp1c and MaSp2b, MaSp2c, MaSp2e, and MaSp2f were significantly enriched in the 8 M compared to 2 M urea supernatants (*P* < 0.05) (Fig. 7E and fig. S41). MaSp3a and MaSp3b were most enriched in 4 M urea supernatants. Next, we compared the protein composition in our proteomics data to the corresponding spatial gene expression in the gland. All the eight proteins enriched in the 8 M urea samples overlapped with the marker genes of ZoneA_MaSp1 and ZoneA_MaSp2 cells. *MaSp3a* and *MaSp3b*, the most significant marker genes in ZoneB_MaSp3 cells, showed the highest relative fraction in the 4 M urea samples (Fig. 7F). Among the eight proteins enriched in the 2 M urea samples, five overlapped with marker genes of either ZoneB_MaSp3 or ZoneC_SpiCE-LMa cells (Fig. 7, E and F).

To ensure that the results obtained from the sequential solubilization of the fibers reflected the proteins’ localization in the fiber and not only their relative solubility in urea, major ampullate silk fibers were completely dissolved in formic acid and subsequently dried. Next, the dried samples were incubated in 2, 4, and 8 M urea, respectively, and the presence of different proteins in the supernatants was determined by LC-MS/MS. If the distribution of the proteins in the supernatants from the intact fibers and dissolved fibers would be similar, the results would support the absence of layers in the fiber. However, proteins that, in our model, reside in the outer layer of the fiber, i.e., AmSp-like1, AmSp-like2, SpiCE-LMa1, SpiCE-LMa2, and SpiCE-LMa4, show a higher relative abundance in the 2 M urea supernatants from intact fibers compared to the dissolved fiber samples (fig. S42). In the 2 M urea supernatants from the dissolved fiber samples, SpiCE-LMa5 and SpiCE-LMa6 show a significantly higher relative abundance compared to those obtained from the intact major ampullate fibers. The reason for the high abundance of these proteins in the 2 M fraction, despite the fact that they are primarily produced by zone A cells, could be attributed to their higher solubility compared to the MaSps (fig. S42). Another contributing factor could be that both these SpiCEs are also expressed in zones B and C, albeit to a lower degree (Figs. 4 and 5).

We further compared the relative abundance of the 18 proteins in the silk extracted at different urea concentrations for both the intact and the dissolved silk samples using pairwise Pearson correlation for all combinations of the samples (fig. S43). The results indicate that the 2 and 4 M intact samples differed the most compared to the other samples (see the dendrogram in fig. S43A). There was a higher variation between the 2 and 8 M intact silk samples than the variation between the 2 and 8 M dissolved samples (fig. S43A). In addition, there was a low correlation between the 2 M dissolved and the 2 M intact silk samples, similar to that found between the 2 and 8 M intact silk samples (fig. S43, B and D). This suggests that the obtained protein profiles in the intact samples are not a result of the differences in protein solubility but reflect the differences in protein composition of the different fiber layers. When comparing any of the intact 8 M samples to any of the dissolved samples, regardless of urea concentration, or when comparing any of the dissolved samples to each other, they all showed a significant positive correlation (*P* < 0.001) (fig. S43, A, C, E, and F), which further contradicts the idea that the solubility of different proteins is the main explanation for the variation observed between the intact silk samples at different urea concentrations. In addition, certain proteins expressed in zones B and C, such as SpiCE-LMa1, SpiCE-LMa2, SpiCE-LMa4, AmSp-like1, and AmSp-like2, are enriched in the 2 M urea extracts from intact silk (fig. S43B). In summary, these data support the inhomogeneous distribution of proteins in the major ampullate silk fiber.

## DISCUSSION

Advanced molecular methodologies, such as scRNA-seq and spatial transcriptomics, serve as pivotal tools in advancing our comprehension of diverse tissue biology. However, their effectiveness depends on well-annotated genomes, posing a substantial challenge when investigating nonmodel organisms. This challenge has been particularly pronounced in investigations of spider silk production due to the nature of spidroin genes, characterized by their substantial size and repetitiveness. Addressing this limitation, we present a high-quality genome assembly of *L. sclopetarius*, featuring nearly complete annotations for all coding genes. Notably, our annotation reveals the presence of 35 full-length (FL) spidroins, which is higher than previously reported (*7*–*11*, *13*). Consistent with prior findings (*7*–*11*, *13*), the spidroins exhibit variable lengths spanning 575 to 9146 amino acid residues (Fig. 1 and table S11). All the spidroin genes encoded proteins with signal peptides, the NT domain, and a repetitive region, and had a stop codon in-frame. Manual inspection of the 3′ downstream sequences revealed no sign of shifted reading frames, which means that the spidroin gene catalog described herein encompasses exclusively complete genes.

Because the focus of this work was to provide a detailed understanding of the structure and function of the major ampullate silk gland, as well as the fiber it produces, we next sought to identify the proteins that make up the major ampullate fiber. This is not a trivial task, because the major ampullate fiber is easily contaminated by other silk types during spinning and collection of the fibers. To avoid these problems, we used LC-MS/MS, both on major ampullate silk fibers continuously collected from a spider mounted under a microscope and on isolated major ampullate glands. By only considering the secretory proteins that were found in both datasets, we could ensure that any contaminating proteins from other glands were removed. Assuming that the most abundant proteins are contributing most to the properties of the fiber, we filtered proteins with abundance of more than 0.1% based on the percentage of total spectra, which allowed us to identify 18 proteins as the predominant constituents in the major ampullate silk. Ten of the 18 proteins came from the four distinct classes of MaSps, namely, MaSp1 to MaSp4. Together, these spidroin proteins constitute 96% of the total protein content of the fiber (Fig. 7G), which is in line with the reported MaSp content in the major ampullate silks from other spider species (*9*, *10*, *13*, *22*, *47*), but the abundance of the different MaSp types differ between fibers from some species (*8*, *9*).

In addition to the MaSps, a previously unreported type of spidroin, which we named AmSp-like, was also found in the fiber. The AmSps were characterized by having an NT domain that grouped with the minor and major ampullate spidroins and a repetitive region, but these spidroins lacked the CT domain and displayed an amino acid composition more similar to the SpiCE proteins than the MaSps (Fig. 2E and fig. S17). Six proteins, designated as SpiCE-LMa1 to SpiCE-LMa6, were identified as constituents of the fiber. Despite their common naming “SpiCE-LMa,” their properties are diverse in terms of molecular weight (table S13), tertiary structure predicted by AlphaFold2 (fig. S26), and amino acid composition (Fig. 2E and fig. S17). The amino acid composition of SpiCE-LMa1 and SpiCE-LMa2 that is expressed in zone C is similar to that of the MaSps, yet they lack both NT and CT domains. While the other SpiCE-LMa proteins either lacked homologs or exhibited homology to hypothetical proteins from other spiders, SpiCE-LMa3 and SpiCE-LMa5 demonstrate similarity to previously reported SpiCE proteins (tables S16 to S20). Specifically, SpiCE-LMa3 shares similarity with SpiCE-CMa2 (produced by spiders from the Caerostris family), while SpiCE-LMa5 shares similarity with SpiCE-NMa4 (Nephilinae family), the latter of which has been designated as a CRP (*9*). Notably, SpiCE-LMa3 to SpiCE-LMa6 proteins that were found to be expressed and secreted from zones A and B have a high cysteine content. To date, only one study has experimentally investigated the impact of SpiCE on the mechanical properties of materials made from recombinant mini-spidroins. A composite film composed of a mini-spidroin and SpiCE-NMa1 had higher tensile strength compared to films comprising the mini-spidroin alone. However, corresponding composite silk fibers display reduced tensile strength (*9*), highlighting the need for further investigation into the functional role of SpiCE proteins in the silk fiber.

To determine which cell types express the 18 silk proteins and their spatial distribution in the gland, we combined three unbiased transcriptomics techniques. First, we used bulk RNA-seq to reveal that all 18 silk genes are expressed in the tail and the sac and not in the duct (Fig. 2D). Thirteen of these were among the top 100 significantly differentially expressed genes in either the tail or the sac of the gland, indicating that the expression profile indeed differs along the gland. Second, scRNA-seq analysis of whole major ampullate glands identified eight cell types. Notably, the marker genes of five of these eight cell types overlapped with all 18 silk genes (Table 2 and Fig. 5B). By cross-referencing the marker genes of the scRNA cell types with the differentially expressed genes found in the bulk RNA data, we were able to assign three cell types to the tail, three to the sac, and two to the duct. The transcriptional profile of cells expressing spider silk proteins could be matched to the bulk RNA-seq data from the tail and sac samples, but not to the samples derived from the duct. This means that the 18 silk proteins are produced by the five cell types located in the tail and sac. To spatially resolve the distribution of the cell types, we used a third transcriptomic technique, 10X Visium. In the sections used for spatial transcriptomics, we first manually annotated the transcriptomic spots within the major ampullate gland zones A, B, and C using the distinct H&E staining pattern and morphology of the epithelium in the respective zones (Fig. 4B). Comparing the expression of genes between the zones revealed that 15 of the 18 silk genes are differentially expressed in at least one of these three zones (Fig. 4C). Next, by integrating the spatial transcriptomics with the single-cell data, the precise localization of the cell types was revealed. In line with the bulk RNA data, the three cell types assigned to the sac were predominantly present in zone B and zone C. Notably, by using the spatial transcriptomics data, we could see a clear distinction between ZoneB_MaSp3 cells that were confined to zone B and ZoneC_SpiCE-LMa cells that were dominant in the zone C epithelium. The three cell types that were assigned to the tail were indeed found in zone A. The tail is long and winding, and by taking advantage of the observation that the cross section of the tail increases along the gland, we could generate information about the spatial location of cell types even within zone A. By determining the perimeter of each cross-sectioned part of the tail, we could order them from proximal (small) to distal (large) (Fig. 6). We found that the ZoneA_MaSp2 cells were primarily localized to the proximal part of zone A, while ZoneA_MaSp1 cells were most abundant in the distal portion of zone A (closer to zone B cells). ZoneA_SpiCE-LMa cells were present in all parts of the zone A (Fig. 6). Last, again in line with the bulk RNA data, the two cell types assigned to the duct were not detected in the tail or sac. Together, our data support that the proteins that make up the major ampullate silk are produced by five cell types that have specific regional anatomical localizations in zones A, B, and C, but not by the cell types confined to the duct.

Next, we sought to connect the expression of the genes in the cell types along the gland to the multiple layers observed in the silk feedstock (Fig. 7A). By using digital image analysis, we showed that the H&E staining of the layers in the dope (the liquid feedstock stored in the sac) as determined by QuPath corresponds to the staining of the intracellular vesicles in the corresponding epithelium. The inner layer of the dope matched the staining of the vesicles in zone A, the middle layer matched the staining of the vesicles in zone B, and the outer layer matched the staining of the vesicles in the zone C epithelial cells (Fig. 7, A and B). This indicates that the three zones indeed produce layered secretions with different protein compositions. Moreover, because we knew the presence of different cell types in zones A, B and C, respectively, and their expression profiles (Fig. 7D), we could predict the protein composition of the different layers in the fiber. Given this model (Fig. 7C), the inner layer of the silk fiber contains the MaSp1, MaSp2, MaSp4, SpiCE-LMa3, SpiCE-LMa5, and SpiCE-LMa6 proteins, and the middle layer primarily contains the MaSp3, AmSp-like1 and AmSp-like2, and SpiCE-LMa2 and SpiCE-LMa4 proteins, while the outer layer is dominated by SpiCE-LMa1, SpiCE-LMa2, and SpiCE-LMa4 but contains no classical spidroins. To test the hypothesis that the layers identified in the gland lumen persist to form layers in the fiber, we ran proteomics analysis on silk fiber extracts after exposure of major ampullate silk fibers to different concentrations of urea (2, 4, and 8 M, respectively). This approach was established by Vollrath *et al.* (*28*) to sequentially dissolve major ampullate silk fibers. Supernatants from fibers incubated in 2 M urea contained proteins primarily expressed in zones B and C, whereas in 8 M urea, which completely dissolve the silk fibers, proteins that are expressed in zone A were enriched (Fig. 7E). When using the 4 M urea, MaSp3a and MaSp3b, which are marker genes for zone B, had the highest relative abundance. These results were further verified to be related to the proteins’ localization in the fiber (figs. S42 and S43). The data presented herein allow us to present a detailed model of the composition of the three-layered major ampullate silk fiber, and to conclude that each layer has a distinct protein composition that is derived from specific cell types confined to zones A, B, and C, respectively (Fig. 7). Because the ZoneA_MaSp2 cells are the dominating cell type in the most proximal part of the tail, it is logical that MaSp2 proteins form the core of the fiber and that the more peripheral regions of the core are dominated by MaSp1 proteins, secreted by the ZoneA_MaSp1 cells that are located more distally in zone A. This finding is in contrast with the report from Hu *et al.* (*13*), which shows that the central core of the *Trichonephila* major ampullate fiber is dominated by MaSp1 proteins and MaSp2 are found more peripherally, but is in line with work by Sponner *et al.* (*32*), who, by biochemical and immunohistochemical investigations of the *Trichonephila* major ampullate silk, revealed the presence of both MaSp1 and MaSp2 in the inner core but exclusively MaSp1 in the peripheral parts of the core. The latter study also concludes that the layer surrounding the core of the fiber (referred to as skin layer) is more tough and resistant to chemical treatment than the outermost layer (coat) and the central core. If these notions are combined with the data presented herein, a plausible conclusion is that the skin layer is dominated by MaSp3 and corresponds to the middle layer in our model.

Thus, what is the purpose of the layered fiber structure? Despite decades of research, the function of each layer is not completely understood. The outermost layer and the skin layer have been suggested to protect against environmental impact (*32*), the skin layer is resistant to protease degradation, and the core of the fiber accounts for most of the mechanical properties of the fiber (*48*). However, Arakawa *et al.* (*12*) have recently published more than 1000 transcriptomes from different spider species along with corresponding mechanical data for the major ampullate fibers and showed that expression of *MaSp3* correlates with higher fiber toughness. Provided that the *MaSp3* is expressed mainly in zone B also in other species, this would suggest that the skin layer could be important for obtaining a high fiber toughness. On the other hand, the presence of layered structures and three epithelial zones in the major ampullate glands from species that do not express *MaSp3* have been reported (*12*, *26*), which suggests that not only the specific protein composition of the fiber layers but also the layered structure in itself may be important for the fiber’s properties. For example, spider species that do not express MaSp3 (*12*) (e.g., Pisauridae and Agelinidae) also have three epithelial zones in the major ampullate gland (*26*), and the Pisauridae spider *Euprosthenops australis* spins a fiber with one of the highest tensile strengths reported (*49*).

Recent publications have pointed toward the idea that formation of recombinant spidroin heterodimers, interconnected in the CT domain, may result in improved mechanical properties of the silk fiber (*19*). Our data suggest that heterodimer formation of spidroins via their CT domains is limited, because the dimerization would occur in the endoplasmic reticulum (ER) of individual cells (*36*, *37*). As can be seen in Fig. 5, there is some overlap of the expression of the MaSps in some cells, but *MaSp1*, *MaSp2*, and *MaSp3* are generally expressed in separate cell types (Fig. 5B). This implies that the spidroins in the soluble state would primarily form dimers composed of one type of spidroin. In the lumen of the sac, just before the duct begins, there is clear evidence that the secretions from the three zones are separated (Fig. 7A) (*26*, *50*). This means that the MaSp1 and MaSp2 spidroins would be found in the zone A “core” secretion, which is surrounded by the zone B secretion that contains MaSp3 (Figs. 4C and 7E). While some diffusion would occur between the layers, our data indicate that the protein composition of the layers mostly stays intact during fiber processing. When the dope travels down the duct, the pH gradient will cause the NT domains to dimerize (*36*, *38*, *39*, *51*), which leads to the notion that the spidroins are linked together in large complexes (*40*). Because the protonatable residues of the *L. sclopetarius* MaSp NT domain are evolutionary conserved (fig. S4) (*38*), inter-class NT dimerization between spidroins, even originating from different layers, could occur. Experimental support for this is currently lacking, but the fiber layers should, in any case, probably not be considered as completely isolated.

Conclusively, we present a model of the major ampullate spider silk, which contains three layers with specific protein compositions. Diffusion may take place across the interface of the layers, possibly making the transition between these gradual, but even if so, the distribution of proteins in the fiber is clearly inhomogeneous. The model is based on several observations: First, the results from the transcriptomics analyses (Figs. 3 and 4) clearly show that the MaSps, AmSp, and SpiCE-LMa proteins are primarily produced in three specific zones along the gland. Second, the H&E staining of the intracellular granules in the epithelium of said zones stain differentially, and the respective staining matches the staining of the three layers observed in the gland lumen, i.e., in the dope. The layered secretions from zones A, B, and C have been observed before and in several distantly related spider species (*26*, *50*), which suggest that this is a common feature of the major ampullate gland and its silk. Third, the proteomics data presented herein support the inhomogeneous distribution of proteins in the fiber and reflect the compositions that would be expected based on a layered structure originating from secretions from the three epithelial zones. Fourth, numerous studies using fiber diffraction, electron microscopy, and light microscopy support the conclusion that the major ampullate silk fiber is layered (*27*–*33*).

This work provides a detailed understanding of the major ampullate gland’s biology and the intricate structure and composition of the major ampullate silk fiber. Specifically, eight distinct glandular cell types were characterized, five of which contribute proteins to the silk feedstock. A high-resolution spatial mapping of these cell types within the major ampullate gland of *L. sclopetarius* was presented along with the identification of several previously uncharacterized genes exhibiting significant DE across the cell types. In addition, 18 spider silk proteins were found to make up the bulk of the fiber. Last, the protein compositions of the three enigmatic layered secretions in the gland were elucidated and correlated to the protein composition of sequentially dissolved fibers. These insights are important for improved bioprocessing of artificial spider silk fibers and should be incorporated in molecular dynamics simulations (*52*) and generative machine learning models (*53*), which hold promise to reveal molecular features that govern the mechanical performance of the fiber.

## METHODS

### Spider samples

*L. sclopetarius* adult female spiders were collected in the wild in a small habitat in Uppsala, Sweden. The taxonomic identity of the spider was verified by the Museum of Natural History, Stockholm, Sweden. The spiders were kept in big containers that allowed them to spin webs. They were fed with meal worms or *Drosophila* flies weekly and watered daily.

### Extraction and sequencing of genomic DNA

High molecular weight (HMW) genomic DNA was extracted from the whole body of one individual *L. sclopetarius* spider using the MagAttract HMW DNA Kit (Qiagen). One adult female spider was anesthetized using dry ice and dissected on ice. The exoskeleton was removed, and all soft tissue was collected to extract HMW DNA. The DNA extraction was done according to the manufacturer’s protocol, except for tissue incubation in RNase and proteinase K at 50°C for 30 min and elution of DNA that was done twice by adding an extra 100 μl of buffer AE to the beads. The purified DNA was run on a 0.5% agarose gel to assess DNA integrity. Absorbance ratios were evaluated on a Nanodrop spectrophotometer and determined to be as follows: 260/280: 1.82 and 260/230: 1.91, resulting in a total of 36.2 μg of DNA. The extracted genomic DNA was also subjected to a quality check using a BioAnalyzer (Agilent), which revealed a single peak at around 11 kb. DNA (10.3 μg) was used to make a 20-kb library. The National Genomics Infrastructure (NGI) platform at SciLifeLab, Uppsala University, performed the library preparation and sequencing using PacBio long-reads and 10X Genomics linked reads.

#### PacBio genomic library preparation and sequencing

The QC-passed DNA samples were sent to the NGI platform at SciLifeLab, Uppsala University, for library preparation and sequencing. The SMRTbell libraries obtained by the TPK1 kit, according to the manufacturer’s instructions, were size-selected at 20 kb using a BluePippin instrument (SAGE) and sequenced on 60 SMRT cells of the RSII instrument using P5-C3 chemistry. For each SMRT cell, 10-hour movies were captured. A total of 798 Gbp of data with an insert size of 11 kb was produced.

#### Library preparation and sequencing using 10X chromium linked reads

The HMW DNA was used to generate the 10X linked read libraries on the 10X Genomics Chromium platform (Genome Library Kit & Gel Bead Kit v2 PN-1000017, genome Chip Kit v2 PN120257) following the manufacturer’s guidelines. The 10X libraries were sequenced on an Illumina NovaSeq6000 instrument (NovaSeq Control Software 1.6.0/RTA version 3.4.4) with 151-bp paired-end setup using the NovaSeqXp workflow in an S4 flow cell. The Bcl-to-FastQ conversion was performed using bcl2fastq_v2.19.1.403 from the CASAVA software suite. Sanger/phred33/Illumina 1.8+ was used as the quality scale.

### Bulk RNA extraction and sequencing of silk glands, head, and abdomen

The spiders were anesthetized using dry ice before they were dissected on ice. After making an incision at the pedicel, the abdomen was gently pinned to a wax plate placed under a Zeiss Stemi 305 stereo microscope, and the exoskeleton was carefully removed with micro scissors to visualize the silk glands. Phosphate-buffered saline (PBS, pH 7.4) was used to wash off the excess nonsilk tissue. With the help of micro tweezers, silk glands (major ampullate glands, minor ampullate glands, flagelliform glands, aggregate glands, and tubuliform glands) were isolated separately by holding their ducts. The aciniform and piriform glands from these five spiders were extracted as a single sample due to difficulties in separating them owing to their small sizes. The major ampullate glands from six additional individuals were cut into three parts: tail, sac, and duct and used for RNA extraction. In another preparation, after removing the exoskeleton, the soft tissue from the whole abdomen was scraped out and used for extracting RNA. The RNA from the head was extracted similarly after removing the legs and the thick exoskeleton. Five replicates were collected for each sample type and RNA was extracted from each replicate separately.

A total of 57 RNA samples were extracted using the RNeasy Plus Mini kit (QiaGen) by following the manufacturer’s protocol. The integrity of the samples was estimated using Tapestation (Agilent Technologies). The transcriptome libraries from the different tissues were generated using Illumina TruSeq Stranded mRNA kit following the manufacturer’s protocol and 151-bp paired end reads were sequenced using the Novaseq6000 instrument.

### PacBio long-read Iso-seq library construction and sequencing for major ampullate glands

RNA was extracted from the major ampullate gland from one individual and homogenized in TriZol. The extracted RNA was sequenced at NGI, Uppsala University, Sweden. RNA QC was performed on the Agilent Bioanalyzer instrument, using the Eukaryote Total RNA Nano kit. The sequencing library was prepared according to PacBio’s Procedure & Checklist—Iso-Seq Express Template Preparation for Sequel and Sequel II Systems, PN 101-763-800 version 02 (October 2019) using the NEBNext Single Cell/Low Input cDNA Synthesis & Amplification Module, the Iso-Seq Express Oligo Kit, ProNex beads, and the SMRTbell Express Template Prep Kit 2.0. The sample (300 ng) was first amplified to 12 cycles, followed by 3 additional cycles, according to the protocol. In the purification of amplified cDNA, the Long Transcripts workflow was applied to obtain material enriched for longer transcripts (>3 kb). The quality control of the SMRTbell libraries was performed with the Qubit dsDNA HS kit and the Agilent Bioanalyzer High Sensitivity kit. Primer annealing and polymerase binding were performed using the Sequel II binding kit 2.0. The samples were sequenced on the Sequel II instrument, using the Sequel II sequencing plate 2.0 and the Sequel II SMRT Cell 8M, with 24 hours of movie time and 2 hours of pre-extension time.

### Single-cell preparation from major ampullate glands of *L. sclopetarius*

A total of 20 spiders were used for single-cell sequencing. The first 10 spiders were anesthetized in dry ice and dissected. All buffers were bubbled with carbogen during and before use. The major ampullate glands were taken out in ringer solution, pH 7.4. The duct was removed. The glands were washed with PBS, pH 7.4, and then incubated into prewarmed trypsin-EDTA (Gibco, 0.5%) at 37°C for 1 min in a low-binding micro-centrifuge tube. The glands were triturated with a pipette briefly and centrifuged at 300*g* for 3 min at 4°C. The pellets were resuspended in 300 μl of Dulbecco’s Modified Eagle’s Medium (DMEM; Gibco) containing 1% bovine serum albumin (BSA; Sigma). The DMEM and the tubes were briefly bubbled with carbogen before resuspension. The suspension was strained through a 40-μm cell strainer and transferred to low-binding micro-centrifuge tubes. Cells were counted using Trypan blue dye.

Because of problems with contaminating droplets of dope that made the separation of single cells challenging, a slightly modified protocol was used for the following 10 spiders. These were processed as described above, but with the exception that the duct was not removed, and the sac was cut and kept in PBS, pH 7.4, for 10 min to allow the dope to flow out. The pieces of glands were picked up and incubated in prewarmed trypsin-EDTA (Gibco, 0.5%) at 37°C for 2 min in a low-binding micro-centrifuge tube. The suspension was triturated with fire-polished glass Pasteur pipette for 1 min, incubated in trypsin-EDTA at 37°C for 2 min, and triturated again with a fire-polished glass Pasteur pipette for 3 min. The suspension was centrifuged at 300*g* for 3 min at 4°C, and the pellet was resuspended in 200 μl of DMEM containing 3% BSA. The suspension was strained through 40 μM prewashed cell strainers into low-binding micro-centrifuge tubes. The strainer was further washed with 100 μl of DMEM containing 3% BSA to reduce the loss of cells. The library preparation was done using the 10X 3′ GE kit on the 10X Chromium Single Cell 3′ Platform following the manufacturer’s protocol (Dual Index 10X_3′_V3.1) and sequenced using Novaseq (100 cycles).

### Sample preparation for spatial transcriptomics

Whole opisthosomas of spiders were flash-frozen in optimal cutting temperature compound medium on an isopentane–dry ice bath and samples were stored at −80°C until use. The samples were sectioned in a cryotome (10 μm) with the knife temperature set at −23°C and the sample holder temperature set at −10°C. Eight sections from five individual spiders were carefully mounted on the capture areas (6.5 × 6.5 mm) of the Visium spatial slide (10X Genomics) and permeabilized for 30 min following the 10X Visium spatial tissue optimization protocol. The libraries were constructed according to Visium spatial gene expression protocol (10X Genomics). For cDNA amplification, 13 to 16 PCR cycles were performed, and for indexing, 13 PCR cycles were used. The sequencing was performed using an SP-200 flow cell on the Illumina Nova-Seq 6000. All the sections were stained with H&E for histological evaluation.

### Genome assembly and polishing

The Falcon and Falcon-Unzip (pb-falcon version 0.2.7) (*54*) de novo assemblers were used to assemble the PacBio data. The initial polishing was done using the Falcon-Unzip polishing module. The Chromium 10X linked read data were used to further polish the long-read CLR assembly. Reads were aligned to the PacBio assembly using Long Ranger (version 2.1.4), and three rounds of polishing were done using Pilon (*55*) (version 1.22) using the diploid flag.

### Genome size and heterozygosity estimation

The raw Illumina reads from 10X Genomics linked sequencing libraries were trimmed using Trimmomatic (*56*), and the canonical 20-mer counts were collected using Jellyfish (*57*). With the 20-mer histogram, GenomeScope2 (*58*) was used to estimate the approximate genome size and heterozygosity.

### Mitochondrial genome assembly

The mitochondrial genome was identified by mapping the assembly to an existing reference spider species, *Neoscona adianta* (GenBank accession: NC_029756.1) using BLAST. The identified regions from the assembly were extracted using BEDTools (*59*). The extracted mitochondrial contigs were then annotated using the MITOS web server (*60*).

### PacBio Iso-seq transcriptome assembly

To generate FL consensus transcript isoforms from the major ampullate gland, the raw polymerase reads were processed using SMRTlink. The subread BAM file was processed to generate the circular consensus sequence reads. These reads were further classified into FL transcript sequences based on the criteria that they contain 5′ primer, 3′ primer, and polyA tails. The FL transcript sequences were processed using the IsoSeq3 platform for generating FL nonchimeric reads, which were further clustered using the ICE algorithm to produce both high- and low-quality polished FL consensus sequences. The high-quality sequences were used for the subsequent analysis. The high-quality transcripts were mapped to the de novo assembled *L. sclopetarius* genome using minimap2 (*61*) (version 2.2.4) with parameters *-ax splice -uf --secondary = no -C5*. The alignment in the SAM format was processed into nonredundant FL transcripts using the “collapse_isoforms_by_sam.py” script from the cDNA-Cupcake tool (https://github.com/Magdoll/cDNA_Cupcake). To assess the completeness of the genome assembly and the annotation, BUSCO (*62*) (version 4.1.4) analysis was performed using the Arachnida_odb10 lineage dataset.

### Genome annotation

The annotation of the de novo assembled *L. sclopetarius* genome was performed using MAKER (*63*) version 3.01.02. High-confidence protein sequences (561,356 proteins) were collected from the UniProt/Swiss-Prot database (downloaded in November 2019), and a specific set of spidroin sequences (1051) were downloaded from NCBI (November 2019).

A repeat library was created using the RepeatModeler package (version 1.0.11, https://repeatmasker.org/RepeatModeler/). Because the spidroin sequences are highly repetitive in nature, the repeats modeled by the RepeatModeler were vetted against our specific set of spidroin dataset. The repeat sequences in the assembled genome were identified using RepeatMasker (version 4.0.9, https://repeatmasker.org/) and repeatRunner (https://yandell-lab.org/software/repeatrunner.html). The tRNAs were identified using tRNAscan (*64*) version 1.3.1 while the conserved noncoding RNAs were identified using the Infernal package (*65*) and the RNA family database, Rfam version 11 (*66*).

The MAKER package was executed in two runs: (i) First, MAKER was used to create a profile using the UniProt/Swiss-Prot protein sequences, the specific set of spidroin sequences, and RNA-seq data from different tissues. An in-house pipeline was used to select a set of genes from this initial evidence-based annotation (first run) and to train Augustus (*67*) and SNAP (*68*). (ii) MAKER was run a second time using the evidence from the first run and the prediction from Augustus. For the construction of gene models, the prediction from Augustus was used.

Functional annotation of genes and transcripts was performed using the translated CDS features for each of the coding transcripts. The protein sequences were searched against UniProt/Swiss-Prot databases, and the specific set of spidroin sequences was scanned using BLAST to retrieve gene names and protein functions. InterProScan (5.30 to 69.0) (*69*) was used for extracting additional annotations (functional domains and sites) from various other biological databases (20 in total).

### Improving 3′ UTR annotation using single-cell data

The aligned BAM files from cell ranger were used for filtering BAM files using UMI-tools FilterBam (*70*) to include reads that were produced with a corrected molecular barcode tag by cell ranger counts. The filtered BAM file was processed to remove PCR duplicates using UMI-tools dedup (*70*). The tool findPeaks from Homer was used to identify peaks (−size 50 -fragLength 100 -minDist 1). The peaks file was converted into a bed file using BEDTools (*59*). These peaks were then either annotated as 3′ untranslated region (UTR) to genes that lacked this feature or reannotated as an extended 3′UTR feature to the nearest genes that were identified within 5000 bp.

### Identification of spidroins and manual curation

For identifying the spidroins in the *L. sclopetarius* genome, two different approaches were used: (i) The translated protein sequences of *L. sclopetarius* were scanned against the PFAM HMM profiles of NT domain, CT domain, and Tubuliform egg casing silk strand structural domains. The HMM profile for these domains, Spidroin_N.hmm (NT domain, PF16763), Spidroin_MaSp.hmm (CT domain, PF11260), and RP1-2.hmm (Tubuliform egg casing silk strand domain, PF12042), were downloaded from the PFAM database. To minimize the risk of false-positive results, the hits with an e-value cutoff below 1e−05 were filtered out. (ii) The reference spidroin sequences were downloaded from the NCBI database (in November 2019). The redundant sequences were removed using CD-HIT at a sequence identity cutoff of 95% and a custom database with FL spidroins (as retrieved from the database); NT and CT domain sequences were created using BLAST package. The spidroin sequences of *L. sclopetarius* were identified by homology search using BLASTp with an e-value cutoff of 1e−05 against the FL reference database. The identified sequences were confirmed for NT and CT domains.

Because of the huge size and high repetitiveness of spidroins, identification of exon boundaries by assembling tools can result in inaccuracies. Thus, the identified *L. sclopetarius* spidroin sequence loci and their surrounding regions (extending 5000 to 10,000 bp on either end of the gene) were manually inspected if those were defined by the automated MAKER gene model. The Web Apollo (*71*) genome browser was used for viewing gene models by entering the transcript identifier and identifying supporting data from bulk RNA-seq and PacBio Iso-seq experiments. The extended gene sequences were searched separately against the custom NT and CT domain reference database using BLASTx with an e-value cutoff of 1e−05. An additional 100-bp region upstream of the identified NT domain region was scanned for signal peptide using SignalP version 6 (*72*). Thus, we assume that the boundaries of spidroin genes were properly defined. The sequences for which the CT domains could not be defined were kept unchanged as per the automated MAKER gene model. We also looked for multiple spidroin genes that were collapsed into single locus due to their high sequence similarity and, therefore, were hard to assemble as separate loci. After defining gene boundaries for every identified spidroin, the gene sequences were translated in all six translational frames and manually inspected for repetitive motifs to identify whether any mis-annotations (missing exons due to incorrect reading frame) existed in the current gene model using the Unipro UGENE software (*73*). On the basis of the identified mis-annotations, either new genes were added, or the existing gene models were replaced with a corrected model. All the corrected sequences were later confirmed by mapping against the reference genome using exonerate. GeneWise (*74*) and Scipio (*75*) were used to generate a GFF file for the corrected gene models.

### Functional assignment to proteins with hypothetical function

To predict function for proteins assigned as “hypothetical protein” (from Genome annotation), Orthofinder (*76*) (version 2.5.2) was used with default settings to identify gene family clusters between *L. sclopetarius, Trichonephila clavipes* (NCBI accession number PRJDB10126), *Trichonephila clavata* (PRJDB10007), *Nephila pilipes* (PRJDB10128), *Trichonephila inaurata madagascariensis* (PRJDB10127), *Argiope bruennichi* (PRJNA629526), and *Araneus ventricosus* (PRJDB7092). The protein sequences were downloaded from NCBI. The putative transcript isoforms were removed from the *L. sclopetarius* proteome dataset, and the longest canonical sequence was kept for the analysis. Proteins from each of the species were processed using Orthofinder-Diamond to assign proteins into orthogroups. In-house python scripts were used to process equivalent genes that were grouped as an orthogroup to further assign functions to hypothetical proteins based on proteins with known functions within the same orthogroup. The annotation was performed at two levels: (i) analyzing homologous clusters assigned to an orthogroup within *L. sclopetarius* and (ii) analyzing gene clusters from other spider species (*T. clavipes, T. clavata*, *N. pilipes*, *T. inaurata madagascariensis*, *A. bruennichi*, and *A. ventricosus*) that were assigned to an orthogroup.

### Analysis of bulk RNA-sequencing data

The raw sequence reads were mapped to the de novo assembled genome using STAR (*77*) (version 2.7). The read counts were generated using featureCounts (*78*) from the Rsubread package (version 2.0.0) (*79*). Transcripts per million (TPM) were used for visualization and comparison of gene expression levels within samples. For PCA and PLS-DA DEseq2 variance stabilization transformation (VST), the DESeq2 package (version 1.38.3) (*80*) was used. For DE analysis between samples, DEseq2 default normalization was used. Genes with TPM >4 in at least one sample, a total of 15,086 genes, were kept for subsequent analysis.

A four-step filtering approach pinpointed variable major ampullate gland-specific genes (fig. S28). Initially, PCA on head and body samples distinguished the tissue types using the first PC, which explained 73.5% of the variation. Genes with a positive first PC loading value were retained, totaling 7777 genes. Subsequently, PCA on major ampullate gland and body samples further differentiated the tissue types, with the first PC accounting for 76.8% of the variation. Genes with a positive first PC loading value, along with *MaSp4* and *SpiCE-LMa4*, resulted in a set of 2776 genes. In the third step, PCA was conducted on tail, sac, and duct samples from the major ampullate gland. The first two principal components explained 77.0% of the sample variation. Genes with a Euclidean distance exceeding 0.13 from the origin were classified as the major ampullate gland-specific gene set and annotated based on their PCA coordinates: tail (PC1 < 0, PC2 ≥ 0), sac (PC1 ≥ 0, PC2 ≥ 0), and duct (PC2 < 0).

Last, PLS-DA was performed on the same samples (tail, sac, and duct) and the major ampullate gland gene set using ropls (version 1.34.0) (*81*). This analysis effectively distinguished tail, sac, and duct samples using two components, based on the *Q* value and visualized in Fig. 3. The PLS-DA loading of genes in the two principal components facilitated the classification of major-specific genes into tail (PC1 < 0), sac (PC1 ≥ 0, PC2 ≤ 0), and duct (PC1 ≥ 0, PC2 > 0). DE analysis using DESeq2 with stringent criteria (adjusted *P* < 0.001, fold change > 4) identified DE genes in each major ampullate gland region (maximum 100 per part). DE analysis compared one part of the major ampullate gland, e.g., tail, against the other two, i.e., sac and duct. VST, PCA loadings, PLS-DA loadings, and DESeq2 fold change for all comparisons of the major ampullate gene set can be found in table S14.

### Analysis of scRNA-seq data

Reads were mapped to transcripts using CellRanger (version 3.0.1, https://support.10xgenomics.com/single-cell-gene-expression/software/pipelines/latest/what-is-cell-ranger). Initial QC analysis removed all cells with fewer than 300 expressed genes and/or less than 500 total transcripts. Only samples with more than 500 cells were retained for further analysis. All steps to separate the different glands and identify corresponding marker genes were performed in Seurat (version 4.0.3, https://satijalab.org/seurat/) (*82*, *83*). Cells were normalized using SCTransform and integrated with canonical correlation analysis distances between samples.

After initial QC steps, 18,539 cells were obtained from seven samples that clustered into 23 clusters. Among these clusters, a subset was identified where the marker genes overlapped with the major ampullate tail, sac, and duct gene sets. By reevaluating this specific subset and restricting the genes analyzed to the intersection of the top 2000 most variable genes from the scRNA-seq data and the major ampullate gene set derived from the bulk RNA analysis, 9700 cells were obtained from seven samples, which clustered into nine groups. The smallest cluster only contained cells from one sample and was removed from further analysis. Marker genes for each cluster were identified using FindAllMarkers from Seurat (version 5.0.3). SCTtransformed values from gene counts, based on mapping using Cell Ranger (version 7.0.1), were used.

### Analysis of spatial transcriptomics data and deconvolution

Eight slides were manually annotated as silk glands based on the morphology using the annotation tool in the Loupe browser (version 6.4.1, https://support.10xgenomics.com/spatial-gene-expression/software/visualization/latest/what-is-loupe-browser). Reads were mapped with Space Ranger (version 1.2.0, https://support.10xgenomics.com/spatial-gene-expression/software/pipelines/latest/what-is-space-ranger) to the reference genome and annotation. Initial QC removed samples with less than 300 genes and 500 transcripts. All steps were carried out in Seurat (version 4.0.3) (*82*, *83*). Cells were normalized using SCTransform and integrated with canonical correlation analysis distances between samples. The distance between the samples was visualized using UMAP. Marker genes for different classes were identified using a subset of 400 cells per class.

For analysis of the major ampullate gland, five samples with good annotation of the gland were kept. The image files were imported in QuPath (version 0.4.3) (*46*) and the regions identified as major ampullate gland were further separated into different zones A, B, and C based on H&E staining and cell morphology using the brush tool. Average eosin, average hematoxylin, and perimeter values were determined for each region using QuPath default parameters. Marker genes for the different zones were identified using gene counts from Space Ranger (version 2.0.1) and findAllMarkers for SCTransformed data from Seurat (version 5.0.3).

Pairwise Pearson correlation was performed for eosin, hematoxylin, and perimeter values for the zone A regions. Zone A regions were split into three classes based on their perimeter value. Regions with a perimeter of less than 500 pixels were assigned proximal, regions with a perimeter larger than 1000 were assigned distal, and the rest were assigned at the middle. Spots on the spatial transcriptomic sections were assigned to the closest region that overlapped with the zone annotation from the QuPath analysis.

To identify the proportion of different cell types on each spot on the slide, we used CARD version 1.0.0 (*84*). Only genes from the single-cell analysis with an average log_2_-fold >2 in at least one cluster was kept to deconvolute the spatial spots. Only genes with at least 200 counts and found in at least 50 spots in the spatial transcriptomics data were kept for the deconvolution analysis. Cell-type proportions were estimated for each spot in the major ampullate gland. The average proportion for the five classes zone C, zone B, and the subclasses proximal, middle, and distal of zone A was calculated by taking the average cell-type proportions from all spots that belonged to the five classes.

### Sample preparation for proteomics

#### Major ampullate glands

The spiders were anesthetized and dissected as mentioned earlier. The major ampullate glands were carefully pulled out by holding the duct using micro tweezers. A cut was made in the sac of the gland allowing the dope to flow out for 15 min. The glands were washed three times with PBS and then transferred to a low-binding 1.5-ml microtube (Axygen) containing 60 μl of 8 M urea in 20 mM tris-HCl at pH 8, vortexed, and sonicated in an ultrasonic bath sonicator (VWR) for 30 min at room temperature. The samples were stored at −20°C until further use. Three biological replicates were used for the final protein sequencing.

Sample aliquots were supplemented with 0.2% ProteaseMAX (Promega) in 20% ACN/20 mM tris-HCl, pH 8, to obtain 4 M urea concentration before water bath sonication for 5 min. Proteins were reduced with 8 mM DTT incubated at 24°C for 1 hour with 550 rpm and alkylated with 20 mM chloroacetamide (CAA) incubated for 1 hour at room temperature in the dark. Digestion was started with the addition of 2 μg of LysC (Wako, Japan) incubated at 24°C for 2 hours and completed with 2 μg of sequencing grade modified trypsin (Promega) incubated at 37°C overnight (ca. 16 hours). Following centrifugation, the supernatants were collected, and proteolysis was stopped with 5% formic acid, and the samples were cleaned on a C18 Hypersep plate with a 40-μl bed volume (Thermo Fisher Scientific) and dried using a vacuum concentrator (Eppendorf).

#### Major ampullate silk fibers

For collecting the silk, each spider was first anesthetized with CO_2_ and gently pinned down to immobilize it, without injuring the animal. By using a Zeiss Stemi 305 stereo microscope, the major ampullate silk was identified, pulled out from the anterior spinneret (*85*) with the help of a tweezer. The silk was collected by rolling it onto a frame attached to a rotating wheel until the spider refused to spin silk. Several spiders were used to collect enough amount of silk for the experiments. After silking, the spiders were fed with fruit flies, watered, and not used again for the next 2 weeks.

The collected silk was treated in three different ways for solubilization since it has been reported that the proteins detected might vary depending on the treatment procedure (*22*). About 450 μg of silk was taken for each set of samples and every treatment was done in triplicates. Three different methods were used to prepare silk samples. In the first method, the silk was dissolved by adding 100 μl of HFIP and brief vortexing. The samples were then sonicated in an ultrasonic bath sonicator (VWR) for 30 min at room temperature. The HFIP was evaporated on a Centrivap concentrator system (Labconco). The protein was resuspended in 60 μl of 8 M urea in 20 mM tris-HCl, pH 8, and stored at −20°C until further use. In the second treatment, the silk was dissolved in 60 μl of 8 M urea in 20 mM tris-HCl at pH 8, sonicated, and stored as mentioned above. For the third method, the silk was dissolved in 60 μl of 9 M LiBr in 20 mM tris-HCl at pH 8, sonicated, and stored as mentioned above. Low-binding 1.5-ml microtubes (Axygen) were used throughout the experiments.

An aliquot of 30-μl samples (ca. 10 μg) was taken to further preparation. From samples with the HFIP and urea methods, proteins were reduced with 3 μl of 100 mM DTT, incubated at 37°C for 3 hours with 1200 rpm, and alkylated with 5 μl of 500 mM CAA incubated for 30 min at room temperature in the dark. Half of the samples were supplemented with 19 μl of 50 mM tris-HCl at pH 8.5 and digested with the addition of 1 μg of LysC (Wako, Japan) incubated at 24°C for 2 hours. Digestion was continued with 1 μg of sequencing-grade modified trypsin (Promega) after the addition of 56 μl of tris-HCl and incubated at 37°C overnight (ca. 16 hours). Samples with the third method (LiBr) were prepared similarly, except that 2 μl of 500 mM DTT was used for reduction, incubated at 95°C for 30 min with shaking at 12,500 rpm. Alkylation with 5 μl of 500 mM CAA (as above) was followed by digestion with LysC and trypsin as described above except that 3 μg trypsin was used. The digestion of all samples was stopped with 6.5 μl of concentrated formic acid, and the samples were cleaned on a C18 Hypersep plate with a 40-μl bed volume (Thermo Fisher Scientific) and dried using a vacuum concentrator (Eppendorf).

#### Layer-wise dissolution of major ampullate silk fibers

The major ampullate silk was collected by allowing each spider to fall freely from a wooden frame, and the extruded silk was rolled onto the same wooden frame. The silk from several individuals was collected in preweighed low-binding microtubes, which were measured again to determine the weight of the collected silk. After forceful silking, the spiders were fed with fruit flies, watered, and were not used again for the next 2 weeks. The collected silk was divided into three sets treated with the following: (i) 2 M urea in 50 mM tris-HCl and 0.5 M NaCl, (ii) 4 M urea in 50 mM tris-HCl and 0.5 M NaCl, and (iii) 8 M urea in 50 mM tris-HCl and 0.5 M NaCl. Samples were sonicated at room temperature for 2 to 3 hours followed by centrifugation at 17,000*g*. The supernatant was collected and stored at −20°C before being used for proteomics analysis. To ensure that the results obtained from sequential solubilization of the silk was not solely due to the relative solubility of the proteins in urea, the following experiment was performed: Two separate samples of the silk weighing 600 μg each were solubilized in 300 μl of formic acid each by vortexing. Each solubilized silk sample was then divided in three low-binding microtubes and subjected to speed vacuum for an hour. The obtained dried samples were further subjected to treatment with the following: (i) 2 M urea in 50 mM tris-HCl, (ii) 4 M urea in 50 mM tris-HCl, and (iii) 8 M urea in 50 mM tris-HCl, respectively. The urea-treated samples were sonicated at room temperature for 2 to 3 hours followed by centrifugation at 17,000*g*. The supernatant was collected and used for proteomics analysis.

#### LC-MS/MS data acquisition

Peptides were reconstituted in solvent A and injected on a 50-cm-long EASY-Spray C18 column (Thermo Fisher Scientific) connected to an UltiMate 3000 nano-flow UPLC system (Thermo Fisher Scientific) using a 90-min-long gradient: 4 to 26% of solvent B (98% acetonitrile, 0.1% FA) in 90 min, 26 to 95% in 5 min, and 95% of solvent B for 5 min at a flow rate of 300 nl/min. Mass spectra were acquired on a Q Exactive HF hybrid quadrupole orbitrap mass spectrometer (Thermo Fisher Scientific) ranging from mass/charge ratio (*m*/*z*) 375 to 1800 at a resolution of *R* = 120,000 (at *m/z* 200) targeting 5 × 10^6^ ions for a maximum injection time of 100 ms, followed by data-dependent higher-energy collisional dissociation fragmentations of precursor ions with a charge state 2+ to 7+, using 45 s dynamic exclusion. The tandem mass spectra of the top 17 precursor ions were acquired with a resolution of *R* = 30,000, targeting 2 × 10^5^ ions for a maximum injection time of 54 ms, setting quadrupole isolation width to 1.4 Th and normalized collision energy to 28%.

### Analysis of proteomics data

Acquired raw data files were converted to Mascot Generic File (*mgf*) format using an in-house developed tool, Raw2MGF (version 2.1.3), and searched with Mascot Daemon version 2.5.1 (Matrix Science Ltd., UK) against a protein database obtained from 22,860 protein entries. A maximum of two missed cleavage sites were allowed for full tryptic digestion, while setting the precursor and the fragment ion mass tolerance to 10 ppm and 0.02 Da, respectively. Carbamidomethylation of cysteine was specified as a fixed modification, while oxidation on methionine as well as deamidation of asparagine and glutamine were set as dynamic modifications. The search results were imported into Scaffold version 4.11 (Proteome Software Inc.) to calculate the contribution of each protein to the total sum of spectra (percentage of total spectra). For all proteins where there was no report of a percentage of total spectra for a sample, the percentage of total spectra value was set to 0.

To identify the proteins in the silk, we used MS/MS data from both the silk fibers and the glands. To determine their presence in the gland, the average percentage of total spectra for each protein was calculated by taking the mean of the three gland samples. All proteins that were reported in at least one of the samples were considered to be present in the gland. The average percentage of total spectra for each protein in the silk was calculated similarly by taking the mean of the nine samples, i.e., three biological replicates for the three different solvents (HFIP, LiBr, and urea). For a protein to be considered present in the major ampullate silk, four criteria had to be fulfilled: (i) it had to be present in the gland, (ii) it had to be found in at least two of the detergents, (iii) it had to have a signal peptide predicted at the 5′ end of its amino acid sequence, and (iv) it had to have an average percentage of total spectra greater than 0.15. After applying these criteria, 18 proteins remained. The relative molecular abundance of proteins in the three HFIP silk samples was calculated using three different methods. MaxQuant (version 2.5.2.0) used iBAQ and LFQ to determine protein relative abundance. In addition, NSAF values, based on spectral counts, were obtained using Scaffold (version 5.3.3). Statistical differences in the percentage of total spectra for a protein between different urea concentrations were determined using an unpaired *t* test, focusing on these 18 proteins. Relative percentage of total spectra for the 18 silk proteins were estimated for urea extracts from fibers dissolved in formic acid and compared with those obtained from urea extracts of intact silk fibers. Pairwise Pearson correlation (*r*) based on the relative abundance of the total spectra of the 18 silk proteins was calculated between all urea extract samples, both intact and dissolved silk. Hierarchical clustering of all the samples was carried out based on the Pearson correlation values to identify similarities in expression patterns between the samples.

### Tissue processing for histological analysis

Spiders were anesthetized with CO_2_ and then dissected at the pedicel on an ice-cooled wax plate using 154 mM sodium chloride solution (Fresenius Kabi AG, Germany). Dissections were performed using a Leica M60 stereomicroscope with a Leica IC80 HD camera. Whole opisthosomas were fixed in 2.5% glutaraldehyde in 67 mM phosphate buffer, pH 7.2, at 4°C for 24 hours, and later rinsed in 67 mM phosphate buffer. Tissues were dehydrated in graded ethanol (50, 70, 90, and 100%, for 30 min each), then infiltrated and embedded in water-soluble glycol methacrylate (Leica Historesin). Sections of 2 μm thickness were obtained using a Leica RM 2165 microtome with glass knives. The sections were stained with H&E and mounted using Agar 100 resin. Evaluation was performed using a Nikon Microphot-FXA (Tekno Optik AB) microscope equipped with a Nikon FX-35DX camera. Images were captured and edited using the software Eclipse Net version 1.20.0.

### Tensile tests of major ampullate silk fibers

Major ampullate silk fibers were reeled and mounted on cardboard frames with a square window of 1 × 1 cm (gauge length, 1 cm). The diameters were measured by means of light microscopy using a Nikon Eclipse Ts2R-FL inverted microscope. The diameter was measured before the tensile test at five locations along the fiber and then averaged. The tensile tests were performed with a 5943-Instron machine (USA) equipped with a 5 N load cell. A strain rate of 6 mm/min was used. Load-displacement curves were converted into engineering stress-strain curves assuming a circular cross section and using the average diameter for each fiber.

## Supplementary Materials

### This PDF file includes:

Supplementary Notes Figs. S1 to S43 References

## References

[1] A. Rising, M. J. Harrington, Biological materials processing: Time-tested tricks for sustainable fiber fabrication. Chem. Rev. 123, 2155–2199 (2023). 36508546 10.1021/acs.chemrev.2c00465

[2] K. Bourzac, Spiders: Web of intrigue. Nature 519, S4–S6 (2015). 25806496 10.1038/519S4a

[3] L. Brunetta, C. L. Craig, *Spider Silk: Evolution and 400 Million Years of Spinning, Waiting, Snagging, and Mating* (Yale Univ. Press, 2010).

[4] D. B. Peakall, Synthesis of silk, mechanism and location. Am. Zool. 9, 71–79 (1969).

[5] F. Vollrath, Strength and structure of spiders’ silks. J. Biotechnol. 74, 67–83 (2000). 11763504 10.1016/s1389-0352(00)00006-4

[6] N. A. Ayoub, J. E. Garb, R. M. Tinghitella, M. A. Collin, C. Y. Hayashi, Blueprint for a high-performance biomaterial: Full-length spider dragline silk genes. PLOS ONE 2, e514 (2007). 17565367 10.1371/journal.pone.0000514 PMC1885213

[7] P. L. Babb, N. F. Lahens, S. M. Correa-Garhwal, D. N. Nicholson, E. J. Kim, J. B. Hogenesch, M. Kuntner, L. Higgins, C. Y. Hayashi, I. Agnarsson, B. F. Voight, The *Nephila clavipes* genome highlights the diversity of spider silk genes and their complex expression. Nat. Genet. 49, 895–903 (2017). 28459453 10.1038/ng.3852

[8] N. Kono, H. Nakamura, R. Ohtoshi, D. A. P. Moran, A. Shinohara, Y. Yoshida, M. Fujiwara, M. Mori, M. Tomita, K. Arakawa, Orb-weaving spider *Araneus ventricosus* genome elucidates the spidroin gene catalogue. Sci. Rep. 9, 8380 (2019). 31182776 10.1038/s41598-019-44775-2 PMC6557832

[9] N. Kono, H. Nakamura, M. Mori, Y. Yoshida, R. Ohtoshi, A. D. Malay, D. A. Pedrazzoli Moran, M. Tomita, K. Numata, K. Arakawa, Multicomponent nature underlies the extraordinary mechanical properties of spider dragline silk. Proc. Natl. Acad. Sci. U.S.A. 118, e2107065118 (2021). 34312234 10.1073/pnas.2107065118 PMC8346794

[10] N. Kono, R. Ohtoshi, A. D. Malay, M. Mori, H. Masunaga, Y. Yoshida, H. Nakamura, K. Numata, K. Arakawa, Darwin’s bark spider shares a spidroin repertoire with *Caerostris extrusa* but achieves extraordinary silk toughness through gene expression. Open Biol. 11, 210242 (2021). 34932907 10.1098/rsob.210242 PMC8692038

[11] P. L. Babb, M. Gregoric, N. F. Lahens, D. N. Nicholson, C. Y. Hayashi, L. Higgins, M. Kuntner, I. Agnarsson, B. F. Voight, Characterization of the genome and silk-gland transcriptomes of Darwin’s bark spider (*Caerostris darwini*). PLOS ONE 17, e0268660 (2022). 35666730 10.1371/journal.pone.0268660 PMC9170102

[12] K. Arakawa, N. Kono, A. D. Malay, A. Tateishi, N. Ifuku, H. Masunaga, R. Sato, K. Tsuchiya, R. Ohtoshi, D. Pedrazzoli, A. Shinohara, Y. Ito, H. Nakamura, A. Tanikawa, Y. Suzuki, T. Ichikawa, S. Fujita, M. Fujiwara, M. Tomita, S. J. Blamires, J.-A. Chuah, H. Craig, C. P. Foong, G. Greco, J. Guan, C. Holland, D. L. Kaplan, K. Sudesh, B. B. Mandal, Y. Norma-Rashid, N. A. Oktaviani, R. C. Preda, N. M. Pugno, R. Rajkhowa, X. Wang, K. Yazawa, Z. Zheng, K. Numata, 1000 spider silkomes: Linking sequences to silk physical properties. Sci. Adv. 8, eabo6043 (2022). 36223455 10.1126/sciadv.abo6043 PMC9555773

[13] W. Hu, A. Jia, S. Ma, G. Zhang, Z. Wei, F. Lu, Y. Luo, Z. Zhang, J. Sun, T. Yang, T. Xia, Q. Li, T. Yao, J. Zheng, Z. Jiang, Z. Xu, Q. Xia, Y. Wang, A molecular atlas reveals the tri-sectional spinning mechanism of spider dragline silk. Nat. Commun. 14, 837 (2023). 36792670 10.1038/s41467-023-36545-6 PMC9932165

[14] J. E. Garb, N. A. Ayoub, C. Y. Hayashi, Untangling spider silk evolution with spidroin terminal domains. BMC Evol. Biol. 10, 243 (2010). 20696068 10.1186/1471-2148-10-243 PMC2928236

[15] M. Xu, R. V. Lewis, Structure of a protein superfiber: Spider dragline silk. Proc. Natl. Acad. Sci. U.S.A. 87, 7120–7124 (1990). 2402494 10.1073/pnas.87.18.7120 PMC54695

[16] M. B. Hinman, R. V. Lewis, Isolation of a clone encoding a second dragline silk fibroin. *Nephila clavipes* dragline silk is a two-protein fiber. J. Biol. Chem. 267, 19320–19324 (1992). 1527052

[17] M. A. Collin, T. H. Clarke III, N. A. Ayoub, C. Y. Hayashi, Genomic perspectives of spider silk genes through target capture sequencing: Conservation of stabilization mechanisms and homology-based structural models of spidroin terminal regions. Int. J. Biol. Macromol. 113, 829–840 (2018). 29454054 10.1016/j.ijbiomac.2018.02.032

[18] J. E. Garb, R. A. Haney, E. E. Schwager, M. Gregoric, M. Kuntner, I. Agnarsson, T. A. Blackledge, The transcriptome of Darwin’s bark spider silk glands predicts proteins contributing to dragline silk toughness. Commun. Biol. 2, 275 (2019). 31372514 10.1038/s42003-019-0496-1 PMC6658490

[19] M. Saric, L. Eisoldt, V. Doring, T. Scheibel, Interplay of different major ampullate spidroins during assembly and implications for fiber mechanics. Adv. Mater. 33, e2006499 (2021). 33496360 10.1002/adma.202006499 PMC11468934

[20] C. Y. Hayashi, R. V. Lewis, Evidence from flagelliform silk cDNA for the structural basis of elasticity and modular nature of spider silks. J. Mol. Biol. 275, 773–784 (1998). 9480768 10.1006/jmbi.1997.1478

[21] K. W. Sanggaard, J. S. Bechsgaard, X. Fang, J. Duan, T. F. Dyrlund, V. Gupta, X. Jiang, L. Cheng, D. Fan, Y. Feng, L. Han, Z. Huang, Z. Wu, L. Liao, V. Settepani, I. B. Thogersen, B. Vanthournout, T. Wang, Y. Zhu, J. Wang, Spider genomes provide insight into composition and evolution of venom and silk. Nat. Commun. 5, 3765 (2014). 24801114 10.1038/ncomms4765 PMC4273655

[22] C. Larracas, R. Hekman, S. Dyrness, A. Arata, C. Williams, T. Crawford, C. A. Vierra, Comprehensive proteomic analysis of spider dragline silk from black widows: A recipe to build synthetic silk fibers. Int. J. Mol. Sci. 17, 1–16 (2016). 10.3390/ijms17091537 PMC5037812 27649139

[23] T. Pham, T. Chuang, A. Lin, H. Joo, J. Tsai, T. Crawford, L. Zhao, C. Williams, Y. Hsia, C. Vierra, Dragline silk: A fiber assembled with low-molecular-weight cysteine-rich proteins. Biomacromolecules 15, 4073–4081 (2014). 25259849 10.1021/bm5011239

[24] J. Kovoor, Comparative structure and histochemistry of silk-producing organs in arachnids, in *Ecophysiology of Spiders*, W. Nentwig, Ed. (Springer, 1987), pp. 160–186.

[25] F. Vollrath, D. P. Knight, Structure and function of the silk production pathway in the spider *Nephila edulis*. Int. J. Biol. Macromol. 24, 243–249 (1999). 10342771 10.1016/s0141-8130(98)00095-6

[26] M. Andersson, L. Holm, Y. Ridderstrale, J. Johansson, A. Rising, Morphology and composition of the spider major ampullate gland and dragline silk. Biomacromolecules 14, 2945–2952 (2013). 23837699 10.1021/bm400898t

[27] S. F. Li, A. J. McGhie, S. L. Tang, New internal structure of spider dragline silk revealed by atomic force microscopy. Biophys. J. 66, 1209–1212 (1994). 8038392 10.1016/S0006-3495(94)80903-8 PMC1275828

[28] F. Vollrath, T. Holtet, H. C. Thøgersen, S. Frische, Structural organization of spider silk. R. Soc. Lond. 263, 147–151 (1996).

[29] S. Frische, A. B. Maunsbach, F. Vollrath, Elongate cavities and skin-core structure in *Nephila* spider silk observed by electron microscopy. J. Microsc. 189, 64–70 (1998).

[30] K. Augsten, P. Muhlig, C. Herrmann, Glycoproteins and skin-core structure in *Nephila clavipes* spider silk observed by light and electron microscopy. Scanning 22, 12–15 (2000). 10768384 10.1002/sca.4950220103

[31] P. Poza, J. Pérez-Rigueiro, M. Elices, J. Llorca, Fractographic analysis of silkworm and spider silk. Eng. Fract. Mech. 69, 1035–1048 (2002).

[32] A. Sponner, W. Vater, S. Monajembashi, E. Unger, F. Grosse, K. Weisshart, Composition and hierarchical organisation of a spider silk. PLOS ONE 2, e998 (2007). 17912375 10.1371/journal.pone.0000998 PMC1994588

[33] I. Iachina, J. Fiutowski, H. G. Rubahn, F. Vollrath, J. R. Brewer, Nanoscale imaging of major and minor ampullate silk from the orb-web spider *Nephila madagascariensis*. Sci. Rep. 13, 6695 (2023). 37095261 10.1038/s41598-023-33839-z PMC10125981

[34] A. Sponner, E. Unger, F. Grosse, K. Weisshart, Differential polymerization of the two main protein components of dragline silk during fibre spinning. Nat. Mater. 4, 772–775 (2005). 16184170 10.1038/nmat1493

[35] D. H. Hijirida, K. G. Do, C. Michal, S. Wong, D. Zax, L. W. Jelinski, 13C NMR of *Nephila clavipes* major ampullate silk gland. Biophys. J. 71, 3442–3447 (1996). 8968613 10.1016/S0006-3495(96)79539-5 PMC1233831

[36] M. Andersson, G. Chen, M. Otikovs, M. Landreh, K. Nordling, N. Kronqvist, P. Westermark, H. Jörnvall, S. Knight, Y. Ridderstråle, L. Holm, Q. Meng, K. Jaudzems, M. Chesler, J. Johansson, A. Rising, Carbonic anhydrase generates CO_2_ and H^+^ that drive spider silk formation via opposite effects on the terminal domains. PLOS Biol. 12, e1001921 (2014). 25093327 10.1371/journal.pbio.1001921 PMC4122339

[37] F. Hagn, L. Eisoldt, J. G. Hardy, C. Vendrely, M. Coles, T. Scheibel, H. Kessler, A conserved spider silk domain acts as a molecular switch that controls fibre assembly. Nature 465, 239–242 (2010). 20463741 10.1038/nature08936

[38] N. Kronqvist, M. Otikovs, V. Chmyrov, G. Chen, M. Andersson, K. Nordling, M. Landreh, M. Sarr, H. Jornvall, S. Wennmalm, J. Widengren, Q. Meng, A. Rising, D. Otzen, S. D. Knight, K. Jaudzems, J. Johansson, Sequential pH-driven dimerization and stabilization of the N-terminal domain enables rapid spider silk formation. Nat. Commun. 5, 3254 (2014). 24510122 10.1038/ncomms4254

[39] M. Landreh, G. Askarieh, K. Nordling, M. Hedhammar, A. Rising, C. Casals, J. Astorga-Wells, G. Alvelius, S. D. Knight, J. Johansson, H. Jörnvall, T. Bergman, A pH-dependent dimer lock in spider silk protein. J. Mol. Biol. 404, 328–336 (2010). 20887730 10.1016/j.jmb.2010.09.054

[40] A. Rising, J. Johansson, Toward spinning artificial spider silk. Nat. Chem. Biol. 11, 309–315 (2015). 25885958 10.1038/nchembio.1789

[41] J. Sparkes, C. Holland, Analysis of the pressure requirements for silk spinning reveals a pultrusion dominated process. Nat. Commun. 8, 594 (2017). 28928362 10.1038/s41467-017-00409-7 PMC5605702

[42] J. Jumper, R. Evans, A. Pritzel, T. Green, M. Figurnov, O. Ronneberger, K. Tunyasuvunakool, R. Bates, A. Žídek, A. Potapenko, A. Bridgland, C. Meyer, S. A. A. Kohl, A. J. Ballard, A. Cowie, B. Romera-Paredes, S. Nikolov, R. Jain, J. Adler, T. Back, S. Petersen, D. Reiman, E. Clancy, M. Zielinski, M. Steinegger, M. Pacholska, T. Berghammer, S. Bodenstein, D. Silver, O. Vinyals, A. W. Senior, K. Kavukcuoglu, P. Kohli, D. Hassabis, Highly accurate protein structure prediction with AlphaFold. Nature 596, 583–589 (2021). 34265844 10.1038/s41586-021-03819-2 PMC8371605

[43] M. Varadi, S. Anyango, M. Deshpande, S. Nair, C. Natassia, G. Yordanova, D. Yuan, O. Stroe, G. Wood, A. Laydon, A. Zidek, T. Green, K. Tunyasuvunakool, S. Petersen, J. Jumper, E. Clancy, R. Green, A. Vora, M. Lutfi, S. Velankar, AlphaFold protein structure database: Massively expanding the structural coverage of protein-sequence space with high-accuracy models. Nucleic Acids Res. 50, D439–D444 (2022). 34791371 10.1093/nar/gkab1061 PMC8728224

[44] P. L. Ståhl, F. Salmén, S. Vickovic, A. Lundmark, J. F. Navarro, J. Magnusson, S. Giacomello, M. Asp, J. O. Westholm, M. Huss, A. Mollbrink, S. Linnarsson, S. Codeluppi, Å. Borg, F. Pontén, P. I. Costea, P. Sahlén, J. Mulder, O. Bergmann, J. Lundeberg, J. Frisén, Visualization and analysis of gene expression in tissue sections by spatial transcriptomics. Science 353, 78–82 (2016). 27365449 10.1126/science.aaf2403

[45] L. McInnes, J. Healy, J. Melville, Umap: Uniform manifold approximation and projection for dimension reduction. arXiv: 1802.03426 [stat.ML] (2018).

[46] P. Bankhead, M. B. Loughrey, J. A. Fernandez, Y. Dombrowski, D. G. McArt, P. D. Dunne, S. McQuaid, R. T. Gray, L. J. Murray, H. G. Coleman, J. A. James, M. Salto-Tellez, P. W. Hamilton, QuPath: Open source software for digital pathology image analysis. Sci. Rep. 7, 16878 (2017). 29203879 10.1038/s41598-017-17204-5 PMC5715110

[47] R. C. Chaw, S. M. Correa-Garhwal, T. H. Clarke, N. A. Ayoub, C. Y. Hayashi, Proteomic evidence for components of spider silk synthesis from black widow silk glands and fibers. J. Proteome Res. 14, 4223–4231 (2015). 26302244 10.1021/acs.jproteome.5b00353 PMC5075943

[48] K. Yazawa, A. D. Malay, H. Masunaga, K. Numata, Role of skin layers on mechanical properties and supercontraction of spider dragline silk fiber. Macromol. Biosci. 19, 1800220 (2019). 10.1002/mabi.201800220 30230228

[49] B. Madsen, Z. Z. Shao, F. Vollrath, Variability in the mechanical properties of spider silks on three levels: Interspecific, intraspecific and intraindividual. Int. J. Biol. Macromol. 24, 301–306 (1999). 10342779 10.1016/s0141-8130(98)00094-4

[50] S. Sonavane, P. Westermark, A. Rising, L. Holm, Regionalization of cell types in silk glands of *Larinioides sclopetarius* suggest that spider silk fibers are complex layered structures. Sci. Rep. 13, 22273 (2023). 38097700 10.1038/s41598-023-49587-z PMC10721825

[51] K. Jaudzems, G. Askarieh, M. Landreh, K. Nordling, M. Hedhammar, H. Jornvall, A. Rising, S. D. Knight, J. Johansson, pH-dependent dimerization of spider silk N-terminal domain requires relocation of a wedged tryptophan side chain. J. Mol. Biol. 422, 477–487 (2012). 22706024 10.1016/j.jmb.2012.06.004

[52] S. Keten, M. J. Buehler, Atomistic model of the spider silk nanostructure. Appl. Phys. Lett. 96, 153701 (2010).

[53] W. Lu, D. L. Kaplan, M. J. Buehler, Generative modeling, design, and analysis of spider silk protein sequences for enhanced mechanical properties. Adv. Funct. Mater. 34, 2311324 (2024).

[54] C. S. Chin, P. Peluso, F. J. Sedlazeck, M. Nattestad, G. T. Concepcion, A. Clum, C. Dunn, R. O’Malley, R. Figueroa-Balderas, A. Morales-Cruz, G. R. Cramer, M. Delledonne, C. Luo, J. R. Ecker, D. Cantu, D. R. Rank, M. C. Schatz, Phased diploid genome assembly with single-molecule real-time sequencing. Nat. Methods 13, 1050–1054 (2016). 27749838 10.1038/nmeth.4035 PMC5503144

[55] B. J. Walker, T. Abeel, T. Shea, M. Priest, A. Abouelliel, S. Sakthikumar, C. A. Cuomo, Q. Zeng, J. Wortman, S. K. Young, A. M. Earl, Pilon: An integrated tool for comprehensive microbial variant detection and genome assembly improvement. PLOS ONE 9, e112963 (2014). 25409509 10.1371/journal.pone.0112963 PMC4237348

[56] A. M. Bolger, M. Lohse, B. Usadel, Trimmomatic: A flexible trimmer for Illumina sequence data. Bioinformatics 30, 2114–2120 (2014). 24695404 10.1093/bioinformatics/btu170 PMC4103590

[57] G. Marcais, C. Kingsford, A fast, lock-free approach for efficient parallel counting of occurrences of k-mers. Bioinformatics 27, 764–770 (2011). 21217122 10.1093/bioinformatics/btr011 PMC3051319

[58] T. R. Ranallo-Benavidez, K. S. Jaron, M. C. Schatz, GenomeScope 2.0 and Smudgeplot for reference-free profiling of polyploid genomes. Nat. Commun. 11, 1432 (2020). 32188846 10.1038/s41467-020-14998-3 PMC7080791

[59] A. R. Quinlan, I. M. Hall, BEDTools: A flexible suite of utilities for comparing genomic features. Bioinformatics 26, 841–842 (2010). 20110278 10.1093/bioinformatics/btq033 PMC2832824

[60] M. Bernt, A. Donath, F. Juhling, F. Externbrink, C. Florentz, G. Fritzsch, J. Putz, M. Middendorf, P. F. Stadler, MITOS: Improved de novo metazoan mitochondrial genome annotation. Mol. Phylogenet. Evol. 69, 313–319 (2013). 22982435 10.1016/j.ympev.2012.08.023

[61] H. Li, Minimap2: Pairwise alignment for nucleotide sequences. Bioinformatics 34, 3094–3100 (2018). 29750242 10.1093/bioinformatics/bty191 PMC6137996

[62] F. A. Simao, R. M. Waterhouse, P. Ioannidis, E. V. Kriventseva, E. M. Zdobnov, BUSCO: Assessing genome assembly and annotation completeness with single-copy orthologs. Bioinformatics 31, 3210–3212 (2015). 26059717 10.1093/bioinformatics/btv351

[63] B. L. Cantarel, I. Korf, S. M. Robb, G. Parra, E. Ross, B. Moore, C. Holt, A. Sanchez Alvarado, M. Yandell, MAKER: An easy-to-use annotation pipeline designed for emerging model organism genomes. Genome Res. 18, 188–196 (2008). 18025269 10.1101/gr.6743907 PMC2134774

[64] T. M. Lowe, S. R. Eddy, tRNAscan-SE: A program for improved detection of transfer RNA genes in genomic sequence. Nucleic Acids Res. 25, 955–964 (1997). 9023104 10.1093/nar/25.5.955 PMC146525

[65] E. P. Nawrocki, D. L. Kolbe, S. R. Eddy, Infernal 1.0: Inference of RNA alignments. Bioinformatics 25, 1335–1337 (2009). 19307242 10.1093/bioinformatics/btp157 PMC2732312

[66] S. W. Burge, J. Daub, R. Eberhardt, J. Tate, L. Barquist, E. P. Nawrocki, S. R. Eddy, P. P. Gardner, A. Bateman, Rfam 11.0: 10 years of RNA families. Nucleic Acids Res. 41, D226–D232 (2013). 23125362 10.1093/nar/gks1005 PMC3531072

[67] M. Stanke, R. Steinkamp, S. Waack, B. Morgenstern, AUGUSTUS: A web server for gene finding in eukaryotes. Nucleic Acids Res. 32, W309–W312 (2004). 15215400 10.1093/nar/gkh379 PMC441517

[68] I. Korf, Gene finding in novel genomes. BMC Bioinformatics 5, 59 (2004). 15144565 10.1186/1471-2105-5-59 PMC421630

[69] T. Paysan-Lafosse, M. Blum, S. Chuguransky, T. Grego, B. L. Pinto, G. A. Salazar, M. L. Bileschi, P. Bork, A. Bridge, L. Colwell, J. Gough, D. H. Haft, I. Letunić, A. Marchler-Bauer, H. Mi, D. A. Natale, C. A. Orengo, A. P. Pandurangan, C. Rivoire, C. J. A. Sigrist, I. Sillitoe, N. Thanki, P. D. Thomas, S. C. E. Tosatto, C. H. Wu, A. Bateman, InterPro in 2022. Nucleic Acids Res. 51, D418–D427 (2023). 36350672 10.1093/nar/gkac993 PMC9825450

[70] T. Smith, A. Heger, I. Sudbery, UMI-tools: Modeling sequencing errors in unique molecular identifiers to improve quantification accuracy. Genome Res. 27, 491–499 (2017). 28100584 10.1101/gr.209601.116 PMC5340976

[71] E. Lee, G. A. Helt, J. T. Reese, M. C. Munoz-Torres, C. P. Childers, R. M. Buels, L. Stein, I. H. Holmes, C. G. Elsik, S. E. Lewis, Web Apollo: A web-based genomic annotation editing platform. Genome Biol. 14, R93 (2013). 24000942 10.1186/gb-2013-14-8-r93 PMC4053811

[72] F. Teufel, J. J. A. Armenteros, A. R. Johansen, M. H. Gíslason, S. I. Pihl, K. D. Tsirigos, O. Winther, S. Brunak, G. von Heijne, H. Nielsen, SignalP 6.0 predicts all five types of signal peptides using protein language models. Nat. Biotechnol. 40, 1023–1025 (2022). 34980915 10.1038/s41587-021-01156-3 PMC9287161

[73] K. Okonechnikov, O. Golosova, M. Fursov; UGENE team, Unipro UGENE: A unified bioinformatics toolkit. Bioinformatics 28, 1166–1167 (2012). 22368248 10.1093/bioinformatics/bts091

[74] E. Birney, M. Clamp, R. Durbin, GeneWise and genomewise. Genome Res. 14, 988–995 (2004). 15123596 10.1101/gr.1865504 PMC479130

[75] O. Keller, F. Odronitz, M. Stanke, M. Kollmar, S. Waack, Scipio: Using protein sequences to determine the precise exon/intron structures of genes and their orthologs in closely related species. BMC Bioinformatics 9, 278 (2008). 18554390 10.1186/1471-2105-9-278 PMC2442105

[76] D. M. Emms, S. Kelly, OrthoFinder: Solving fundamental biases in whole genome comparisons dramatically improves orthogroup inference accuracy. Genome Biol. 16, 157 (2015). 26243257 10.1186/s13059-015-0721-2 PMC4531804

[77] A. Dobin, C. A. Davis, F. Schlesinger, J. Drenkow, C. Zaleski, S. Jha, P. Batut, M. Chaisson, T. R. Gingeras, STAR: Ultrafast universal RNA-seq aligner. Bioinformatics 29, 15–21 (2013). 23104886 10.1093/bioinformatics/bts635 PMC3530905

[78] Y. Liao, G. K. Smyth, W. Shi, FeatureCounts: An efficient general purpose program for assigning sequence reads to genomic features. Bioinformatics 30, 923–930 (2014). 24227677 10.1093/bioinformatics/btt656

[79] Y. Liao, G. K. Smyth, W. Shi, The R package Rsubread is easier, faster, cheaper and better for alignment and quantification of RNA sequencing reads. Nucleic Acids Res. 47, e47 (2019). 30783653 10.1093/nar/gkz114 PMC6486549

[80] M. I. Love, W. Huber, S. Anders, Moderated estimation of fold change and dispersion for RNA-seq data with DESeq2. Genome Biol. 15, 550 (2014). 25516281 10.1186/s13059-014-0550-8 PMC4302049

[81] E. A. Thévenot, A. Roux, Y. Xu, E. Ezan, C. Junot, Analysis of the human adult urinary metabolome variations with age, body mass index, and gender by implementing a comprehensive workflow for univariate and OPLS statistical analyses. J. Proteome Res. 14, 3322–3335 (2015). 26088811 10.1021/acs.jproteome.5b00354

[82] R. Satija, J. A. Farrell, D. Gennert, A. F. Schier, A. Regev, Spatial reconstruction of single-cell gene expression data. Nat. Biotechnol. 33, 495–502 (2015). 25867923 10.1038/nbt.3192 PMC4430369

[83] Y. Hao, S. Hao, E. Andersen-Nissen, W. M. Mauck III, S. Zheng, A. Butler, M. J. Lee, A. J. Wilk, C. Darby, M. Zager, P. Hoffman, M. Stoeckius, E. Papalexi, E. P. Mimitou, J. Jain, A. Srivastava, T. Stuart, L. M. Fleming, B. Yeung, R. Satija, Integrated analysis of multimodal single-cell data. Cell 184, 3573–3587.e29 (2021). 34062119 10.1016/j.cell.2021.04.048 PMC8238499

[84] Y. Ma, X. Zhou, Spatially informed cell-type deconvolution for spatial transcriptomics. Nat. Biotechnol. 40, 1349–1359 (2022). 35501392 10.1038/s41587-022-01273-7 PMC9464662

[85] R. Foelix, *Biology of Spiders* (Oxford Univ. Press, 1996).

[86] Y. Perez-Riverol, J. Bai, C. Bandla, D. Garcia-Seisdedos, S. Hewapathirana, S. Kamatchinathan, D. J. Kundu, A. Prakash, A. Frericks-Zipper, M. Eisenacher, M. Walzer, S. Wang, A. Brazma, J. A. Vizcaino, The PRIDE database resources in 2022: A hub for mass spectrometry-based proteomics evidences. Nucleic Acids Res. 50, D543–D552 (2022). 34723319 10.1093/nar/gkab1038 PMC8728295

[87] R. Challis, E. Richards, J. Rajan, G. Cochrane, M. Blaxter, BlobToolKit—Interactive quality assessment of genome assemblies. Genetics 10, 1361–1374 (2020). 10.1534/g3.119.400908 PMC7144090 32071071

[88] T. H. Clarke, J. E. Garb, R. A. Haney, R. C. Chaw, C. Y. Hayashi, N. A. Ayoub, Evolutionary shifts in gene expression decoupled from gene duplication across functionally distinct spider silk glands. Sci. Rep. 7, 8393 (2017). 28827773 10.1038/s41598-017-07388-1 PMC5566633

[89] L. J. McGuffin, K. Bryson, D. T. Jones, The PSIPRED protein structure prediction server. Bioinformatics 16, 404–405 (2000). 10869041 10.1093/bioinformatics/16.4.404

