# Supplementary material for: Origin, structure, and composition of the spider major ampullate silk fiber revealed by genomics, proteomics, and single-cell and spatial transcriptomics
Source: Sci Adv. 2024 Aug 14;10(33):eadn0597. doi: 10.1126/sciadv.adn0597 (PMC11323941; doi:10.1126/sciadv.adn0597)
Supplement: Supplementary file 1 — Supplementary Notes Figs. S1 to S43 References [file sciadv.adn0597_sm.pdf]

Science Advances
AAAS

Supplementary Materials for

Origin, structure, and composition of the spider major ampullate silk fiber revealed by genomics, proteomics, and single-cell and spatial transcriptomics

Sumalata Sonavane *et al.*

Corresponding author: Johan Reimegård, johan.reimegard@scilifelab.se; Anna Rising, anna.rising@ki.se

Sci. Adv. **10**, eadn0597 (2024)
DOI: 10.1126/sciadv.adn0597

The PDF file includes:

Supplementary Notes
Figs. S1 to S43
References

Other Supplementary Material for this manuscript includes the following:

Tables S1 to S20

Supplementary Notes

A complete spidroin catalogue from *L. sclopetarius* genome

High molecular weight genomic DNA was extracted from a single adult female *L. sclopetarius* spider (table S1) and sequenced using PacBio and 10X platforms. The combination of short- and long-read sequencing methods allowed us to build a complete and a high-quality *de-novo* assembly (statistics summarized in Table 1), with the final genome size of 2.27 Gb which was in good agreement with the predicted genome size of 2.1 Gb estimated using GenomeScope2 (*58*). The assembly was composed of 1602 contigs (average length 0.119 Mbp) with 30.5% GC content. A comparison of the assembly metrics with the other reported spider genomes is shown in table S2. The completeness of the genome assembly was 97.3 % according to Benchmarking Universal Single Copy Orthologs (BUSCO) (*62*) using the Arachnida_odb10 lineage (table S3).

To identify possible contamination in the genome assembly, BlobToolKit (version 4.3.6) (*87*) was used to find various markers indicative of contaminant DNA in the genome. This tool screened the genome for unexpected coverage, GC content, and sequence similarity, effectively ruling out significant contamination (fig. S2). It is worth noting that 16 contigs (0.03% of the assembly) were flagged as potentially belonging to other species. Examining these contigs further revealed that BLAST hits were matching only to small portions, suggesting they might be novel sequences rather than contaminants. Taken together, these results indicate negligible contamination in the final assembly.

The *de-novo* assembled *L. sclopetarius* genome was annotated using multiple approaches to predict genes, repetitive elements, and non-coding RNA. To further improve the annotation, bulk transcriptomic data from several tissue types was used, namely, freshly dissected silk glands (major ampullate, minor ampullate, flagelliform, aggregate, piriform + aciniform glands), as well as abdomen and head from five female individuals (Fig. 1A; table S1). Tubuliform glands were not used since the spiders were not in reproductive phase. After annotation, 22860 protein-coding genes were identified, of which 17886 (78%) genes were functionally annotated and 13524 (59%) were found in the Uniprot database (table S3). A BUSCO analysis using the Arachnida odb10 lineage resulted in 96.8 % completeness of the gene set (table S3).

Since it is known that the spidroins make up the bulk of the silk fiber, a complete spidroin gene catalog was generated for *L. sclopetarius* by screening the transcriptome for spidroin N- and C-terminal domains based on homology to known spidroins (tables S4 and S5), by identifying the repeat motifs, and by manual curation. We identified 35 full-length spidroin genes and successfully assigned them to different spidroin classes based on homology to reported spidroins, phylogenetic analyses of the terminal domains, as well as the presence of distinct repetitive motifs in the repeat regions (Fig. 1, C to F, tables S6 to S11, and figs. S3 and S4). At least one paralogue from each of the seven spidroin orthologous groups were identified. The classification was supported by the phylogenetic analysis of the N-terminal domains in which the 35 spidroins clustered according to spidroin type (Fig. 1C). The spidroin expression profiles in the different silk glands were evaluated using the bulk RNA sequencing data from silk glands, abdomen, and head. In general, the different spidroin types were highly expressed in their corresponding silk glands, i.e., *MaSps* were most highly expressed in major ampullate glands, *MiSps* in minor ampullate glands, and so on (Fig. 1D). However, as reported previously (*88-90*), some spidroins were expressed across multiple glands. There was no significant spidroin expression in the head.

All the spidroins were composed of a signal peptide and the conserved N-terminal domain but four spidroins lacked the canonical C-terminal domain. However, these four genes had stop codons in frame, indicating that the genes were complete. No evidence of shifted reading frames was found when 5000 nucleotides downstream of the stop codon were scrutinized for the presence of repeat elements and the C-terminal domain in any of the three reading frames for these four genes. Since classical spidroins are defined as having the evolutionary conserved terminal domains flanking a repetitive part (*14*), we decided to name these genes as spidroin-like. One of the spidroin-like genes encoded a non-repetitive region C-terminally before the stop-codon and was annotated as flagelliform spidroin-like (FlSp-like) due to the clustering of its N-terminal domain with other FlSps and its predominant expression was in the flagelliform gland (Fig. 1, C and D). The other three spidroin-like proteins completely lacked a non-repetitive region before the stop codon and the amino acid sequence in their repetitive regions did not show homology to any reported spidroin. Two of these were designated as ampullate spidroin-like (AmSp-like) 1 and 2, based on clustering of their N-terminal domains with ampullate spidroins in phylogenetic analysis, as well as on their expression in both ampullate glands (Fig. 1, C and D). The N-terminal domain of the remaining spidroin-like protein clustered with aggregate spidroins and was expressed in aggregate glands, and was hence

named aggregate spidroin-like (AgSp-like). Of the 35 spidroins, 12 genes belonged to the MaSp family which were further classified as MaSp1–4 based on the type and frequency of repetitive motifs (tables S8 and S10, and Figure 1, E and F). A multiple sequence alignment of the *L. sclopetarius* spidroin terminal domains show that they are well conserved and predicted to have the reported respective helical segments (figs. S5 to S10).

Gene expression profiles separate the silk glands

In order to assess the gene expression profiles in different silk glands, the bulk RNA sequencing data was used for hierarchical clustering of gene expression profiles (fig. S11). It revealed that the silk glands have more similar gene expression compared to the head and the abdomen, and that the minor and the major ampullate glands cluster together.

To further verify gene expression profiles in the silk glands, 10X Visium spatial transcriptomics was used. This is an unbiased and elegant technique that allows mapping the gene expression to the tissues with a resolution of 50 μm (*44*). Shortly, cryo-embedded tissue sections are placed on special glass slide with capture areas containing 5000 barcoded spots each. Each spot (~55 μm) harbors millions of capture probes with poly(dT) segments, that bind the polyA tail of mRNAs, a unique molecular identifier, and a spatial barcode which allow the RNA to be traced back to a specific spot on the spatial section. Once placed on the capture area, the tissue is stained and imaged followed by tissue permeabilization, cDNA synthesis and sequencing. Six sections of the whole abdomen from four female individuals were used for final sequencing experiments. The spots on the hematoxylin and eosin (H&E) stained sections were manually annotated to different silk glands based on cell histology (one section is shown in Fig. 3, A and B; figs. S12 and S13 show additional sections). The sequencing data from all spots from all the sections were then isolated and visualized using Uniform Manifold Approximation and Projection (UMAP) (*45*). In the UMAP, the spots assigned as silk glands clustered together and separated from the spots belonging to other tissue types (fig. S14). Within the silk gland cluster, the spots from different silk glands clustered together, in line with the manual annotation (Fig. 3C). On the spatial sections, the spidroin expression was mostly specific to corresponding glands (fig. S15) and confirmed the results from the bulk-RNA expression profiles. The bulk RNA and the spatial RNA transcriptomic analyses together show that the silk glands share a common gene set which separates them from the other tissues as well as display gene expression profiles that are unique to each gland.

Supplementary Figures

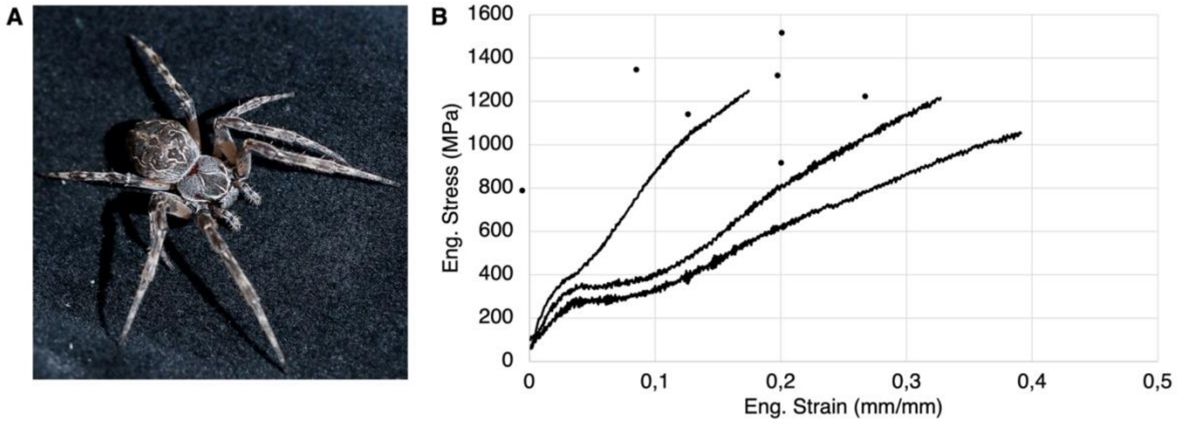

Fig. S1: Swedish bridge spider, *Larinioides sclopetarius*, and stress-strain curves of its dragline silk.
(A) A female *L. sclopetarius* spider. These spiders were selected because they are abundant in Europe and easily accessible in Uppsala. They spin a major ampullate fiber with impressive mechanical properties (B) and belong to the Araneidae family (commonly known as orb weavers). The spiders were caught in the wild in Uppsala, Sweden. **(B)** Stress-strain curves of the dragline silk obtained from forcefully silked spiders. X-axis represents the engineering strain in mm/mm and y-axis represents engineering stress in MPa. Photo Credit for (A): Lena Holm, Swedish University of Agricultural Sciences.

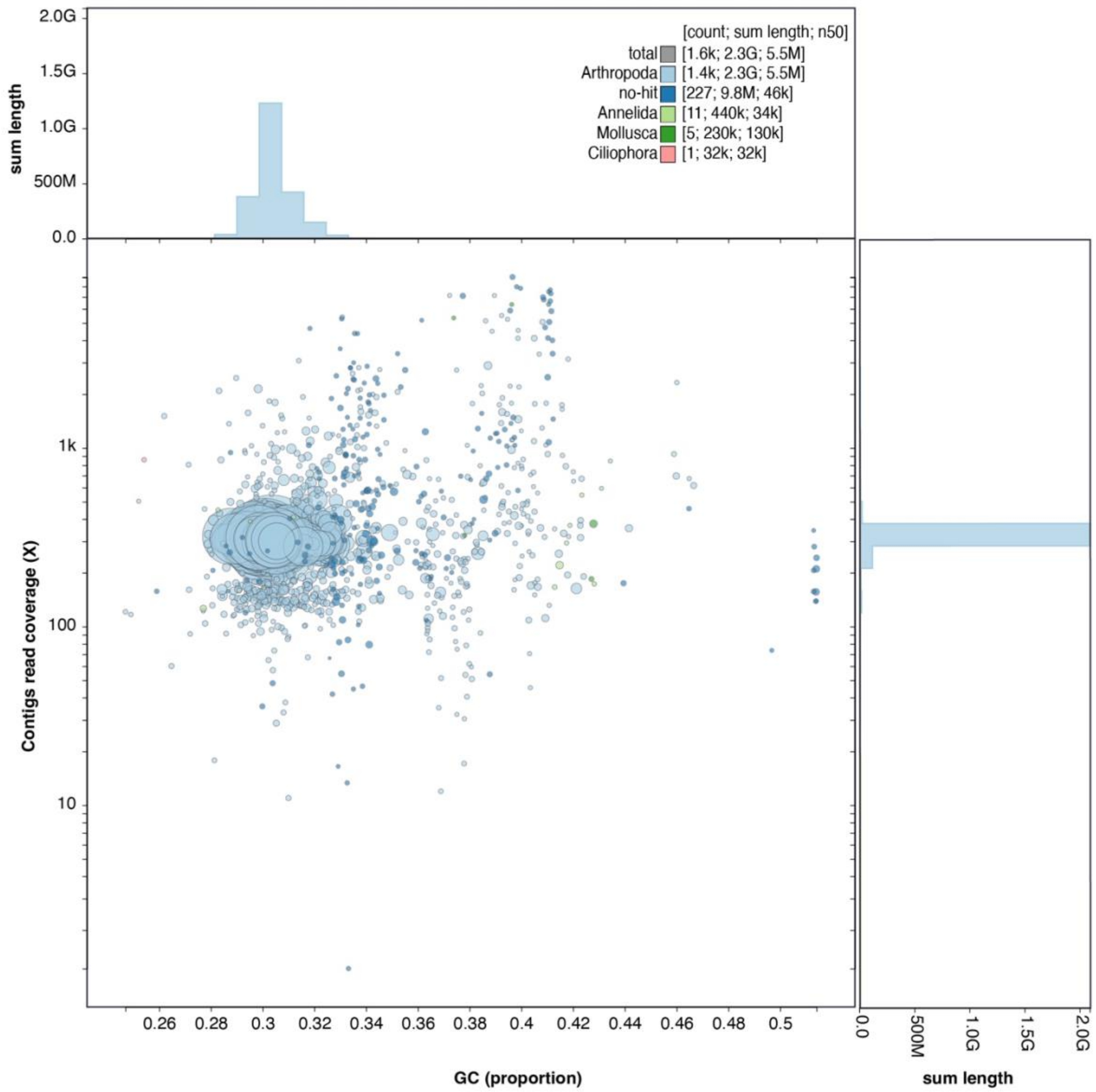

Fig. S2: Blob plot for the assembled contigs from *L. sclopetarius*.

Blob plot for all the assembled contigs from the *L. sclopetarius* genome generated using BlobToolKit. X-axis represents GC proportion and y-axis represents the contigs read coverage, a measure of how many times each contig was sequenced. Each dot/blob represents a contig with diameter proportional to the contig length. The colours indicate taxonomic affiliation of the contigs. The legend reflects the count (total number of contigs), sum length (cumulative length of contigs), and N50 (length of the shortest contig at 50% of the total assembly length) under each taxonomic affiliation.

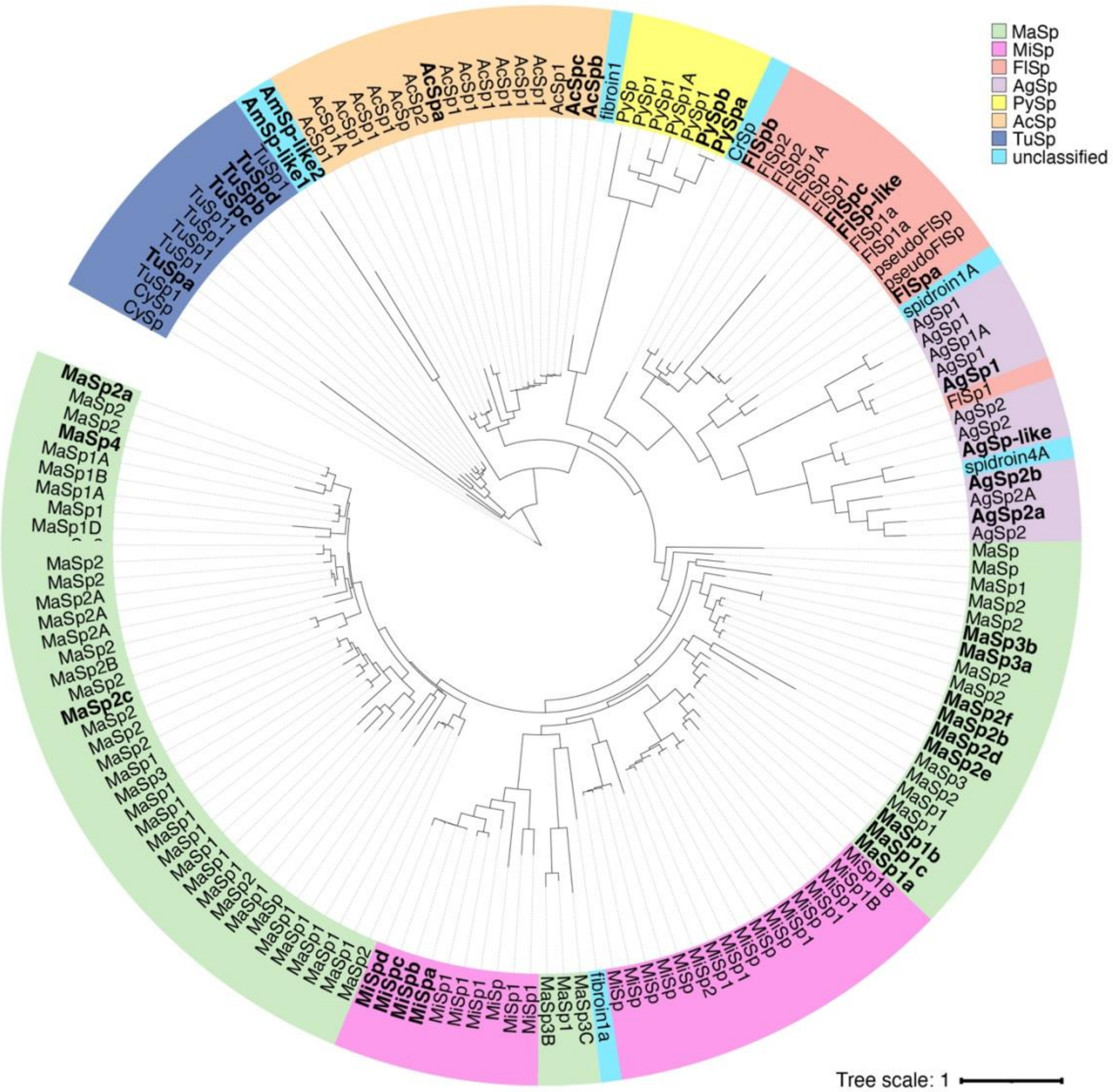

Fig. S3: Phylogenetic tree for spidroin N-terminal domains.

Maximum-likelihood phylogenetic tree for N-terminal domains extracted from 124 sequences from the NCBI database (listed in table S6) and N-terminal domains of *L. sclopetarius* spidroins (in bold). The multiple sequence alignment was performed using Clustal Omega and the consensus tree was built using RAxML. The spidroin classes are indicated in different colours.

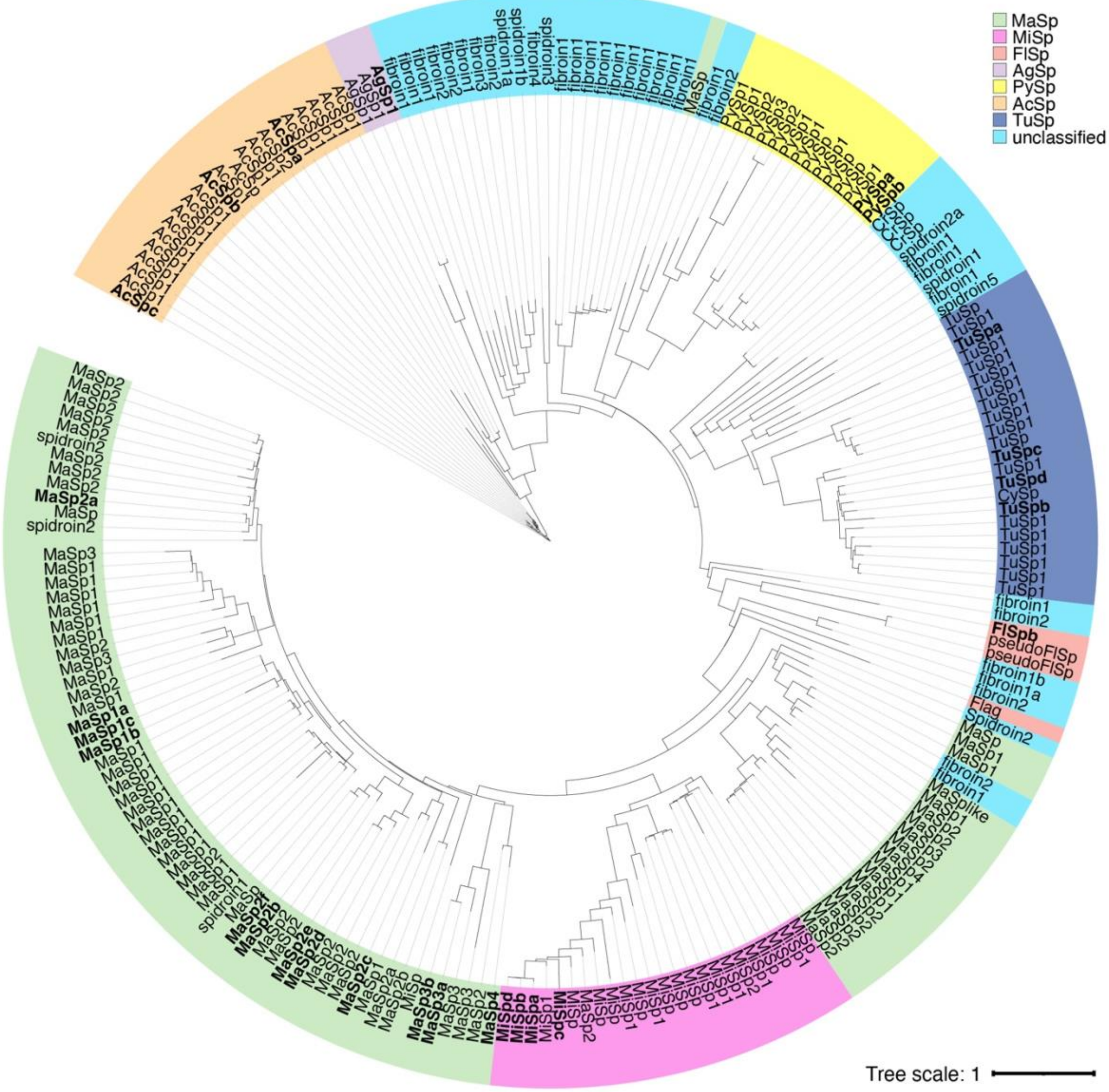

Fig. S4: Phylogenetic tree for spidroin C-terminal domains.

Maximum-likelihood phylogenetic tree for C-terminal domains extracted from 185 sequences from the database (listed in table S7) and C-terminal domains of *L. sclopetarius* spidroins (in bold). The multiple sequence alignment was performed using Clustal Omega and the consensus tree was built using RAxML. The spidroin classes are indicated in different colours.

In figures S5 to S10, the multiple sequence alignments were performed using Clustal Omega and visualized using Chimera. The color coding of amino acid residues is according to the Clustal Omega:
(https://www.rbvi.ucsf.edu/chimera/docs/ContributedSoftware/multalignviewer/colprot.par).

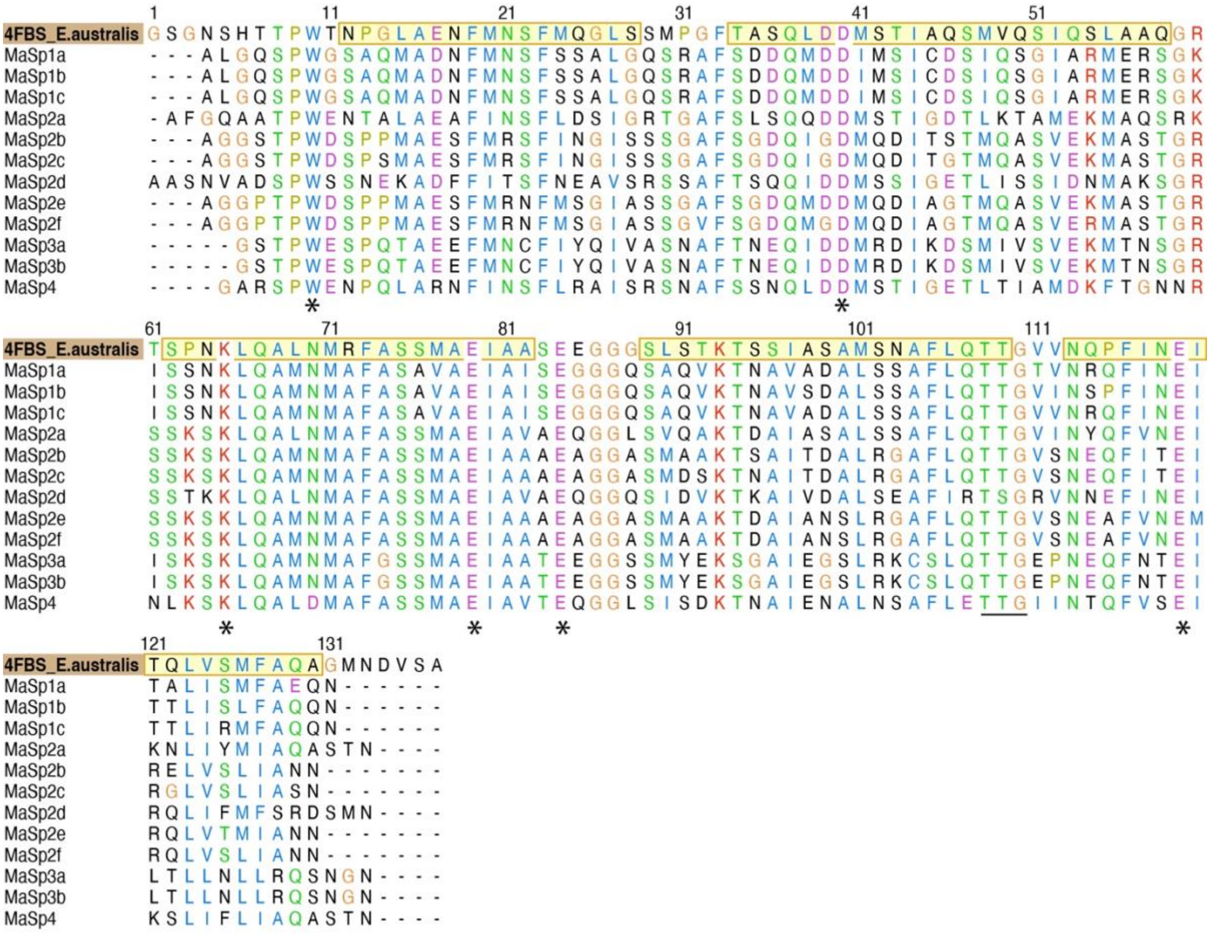

Fig. S5: Alignment of N-terminal domains of *L. sclopetarius* MaSp proteins.
N-terminal domain of *E. australis* MaSp (PDB code: 4FBS) was used as a reference sequence. The light-yellow boxes on 4FBS represent helices and the black stars indicate the amino acid residues that are important for dimerization of N-terminal domain. The conserved TTG motif is underlined.

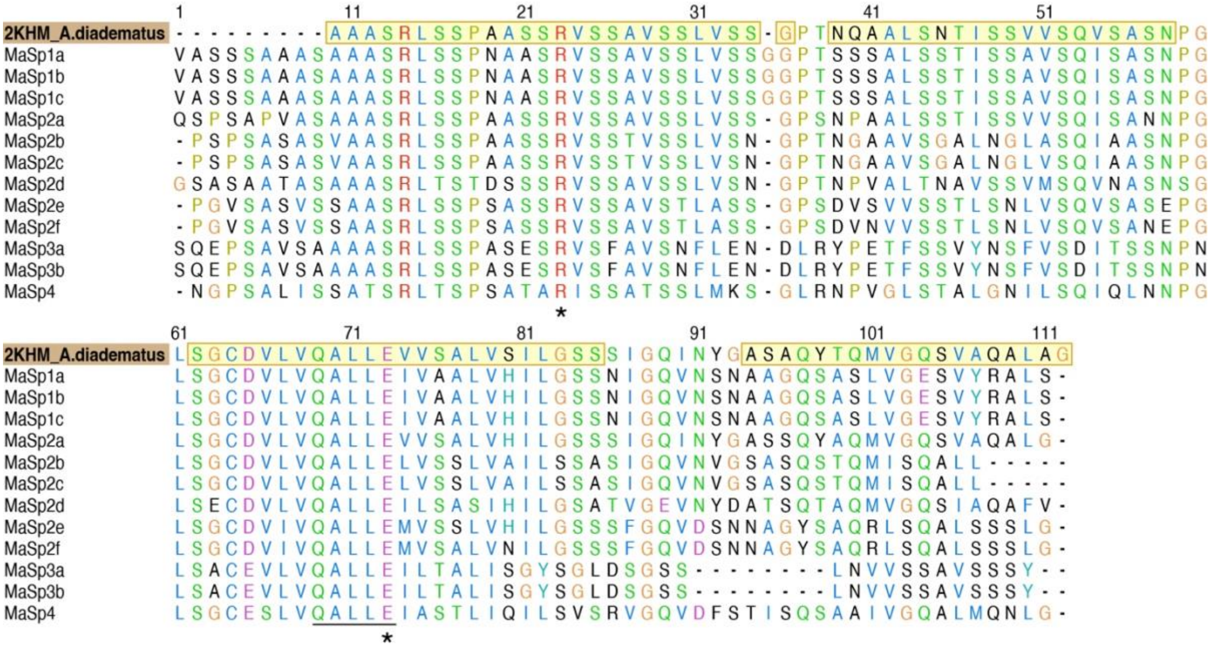

Fig. S6: Alignment of C-terminal domains of *L. sclopetarius* MaSp proteins.
The C-terminal domain from *A. diadematus* MaSp (PDB code: 2KHM) was used as a reference. The light-yellow boxes on 2KHM represent helices and the black stars indicate the amino acid residues that have been reported to participate in the formation of salt bridge. The conserved QALLE motif is underlined.

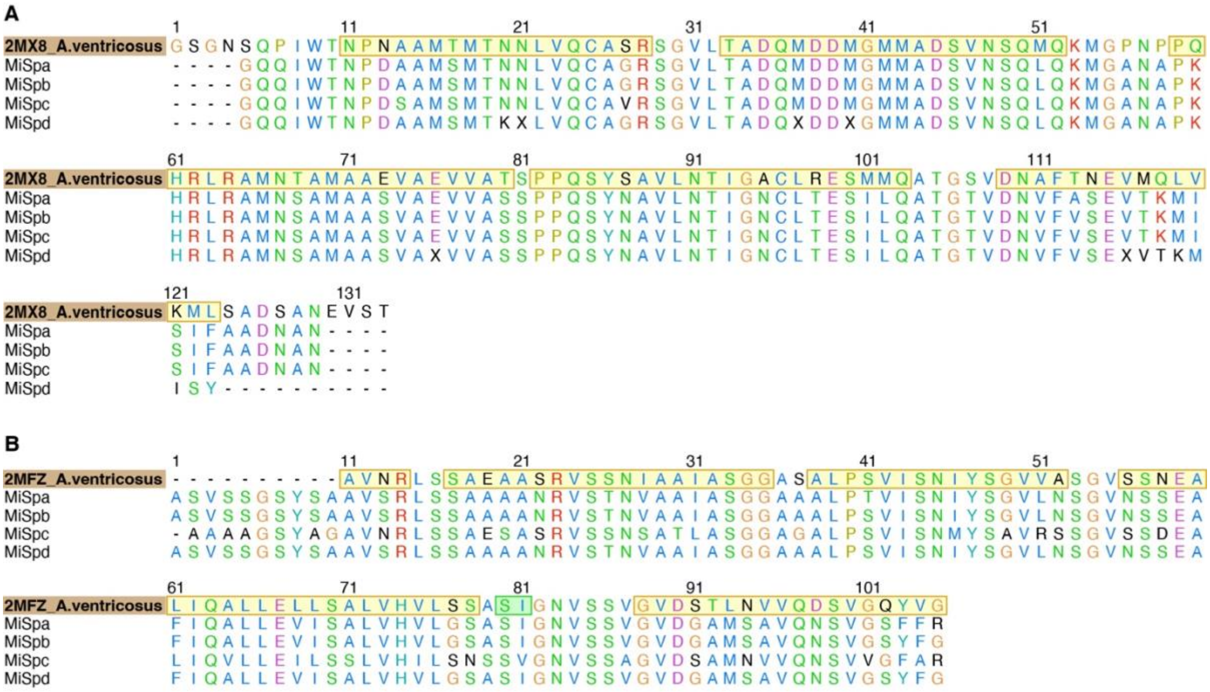

Fig. S7: Alignment of N- and C-terminal domains of *L. sclopetarius* MiSp proteins.
The N-terminal **(A)** and C-terminal **(B)** domains of *L. sclopetarius* MiSp proteins aligned with corresponding domains from *A. ventricosus* MiSp (PDB codes: 2MX8 and 2MFZ). The light-yellow boxes on 2MX8 and 2MFZ represent helices.

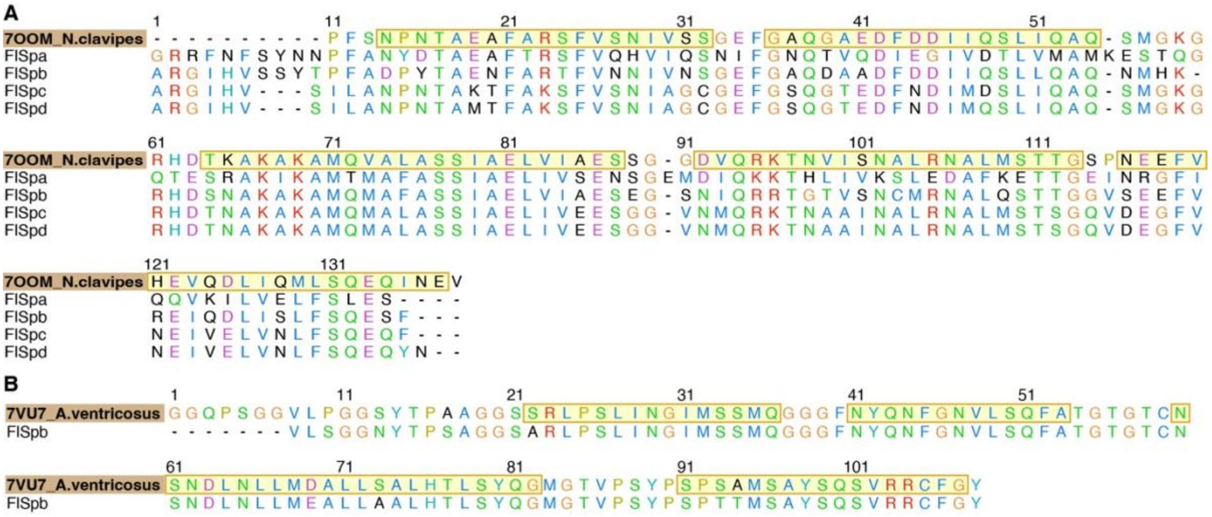

Fig. S8: Alignment of N- and C-terminal domains of *L. sclopetarius* FlSp proteins.

(A) The N-terminal domains of *L. sclopetarius* FlSp aligned with N-terminal domain from *N. clavipes* FlSp (PDB code: 7OOM). **(B)** C-terminal domains of *L. sclopetarius* FlSp aligned with C-terminal domain from *A. ventricosus* FlSp (PDB code: 7VU7). The light-yellow boxes on 7OOM and 7VU7 represent helices.

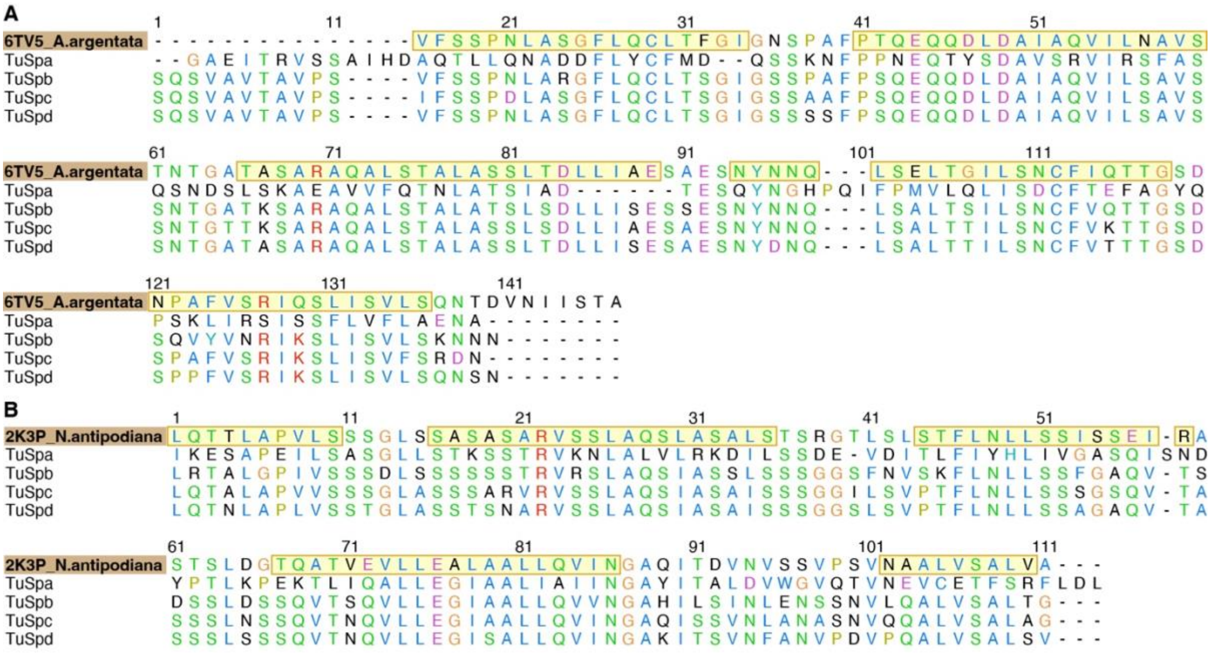

Fig. S9: Alignment of N- and C-terminal domains of *L. sclopetarius* TuSp proteins.

(A) The N-terminal domains of *L. sclopetarius* TuSp aligned with the corresponding domain from *A. argentata* TuSp (PDB code: 6TV5). **(B)** C-terminal domain from *N. antipodiana* (PDB code: 2K3P) was used as reference to align the C-terminal domains of *L. sclopetarius* TuSps. The light-yellow boxes on 6TV5 and 2K3P represent helices.

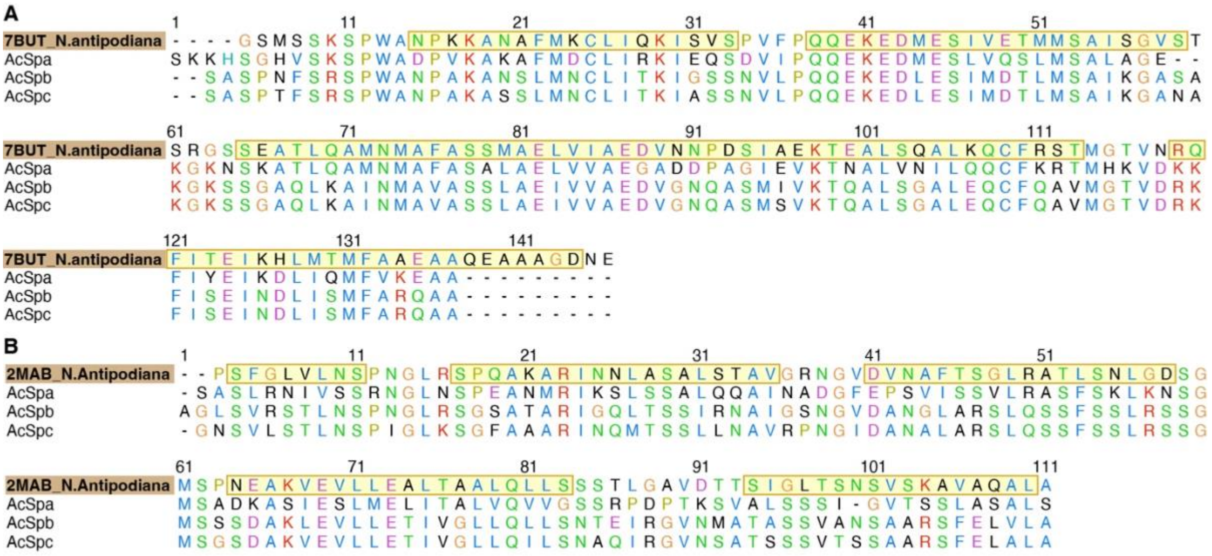

Fig. S10: Alignment of N- and C-terminal domains of *L. sclopetarius* AcSp proteins.
The N- and C-terminal domains from *N. antipodiana* AcSp (PDB code: 7BUT and 2MAB) were used as reference sequences to align the *L. sclopetarius* AcSp N-terminal **(A)** and C-terminal **(B)** domains. The light-yellow boxes on 7BUT and 2MAB indicate helices.

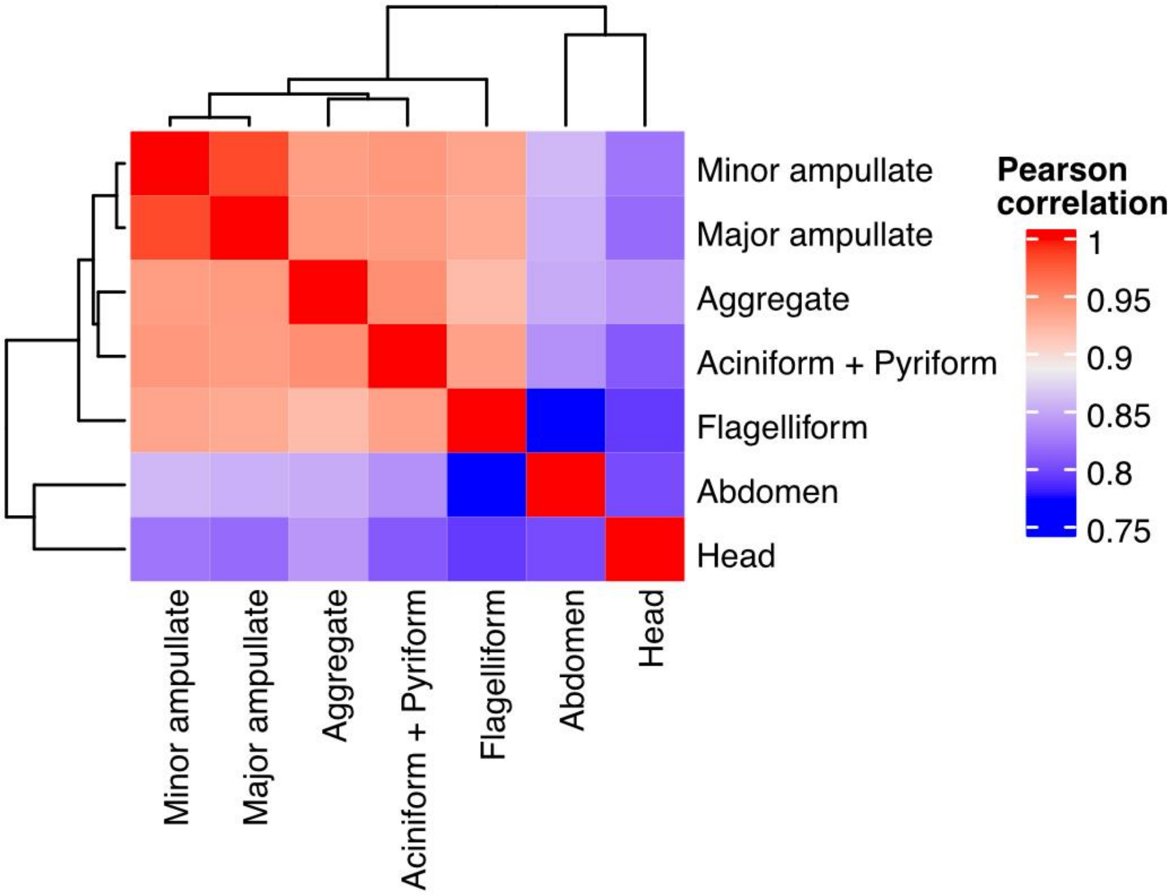

Fig. S11: Bulk RNA analysis of silk glands of *L. sclopetarius.*
Heatmap showing pairwise Pearson correlation based on the average log values for overall gene expression between the different samples of bulk RNA data. Tissues are clustered using hierarchical clustering with Euclidian distance and complete linkage.

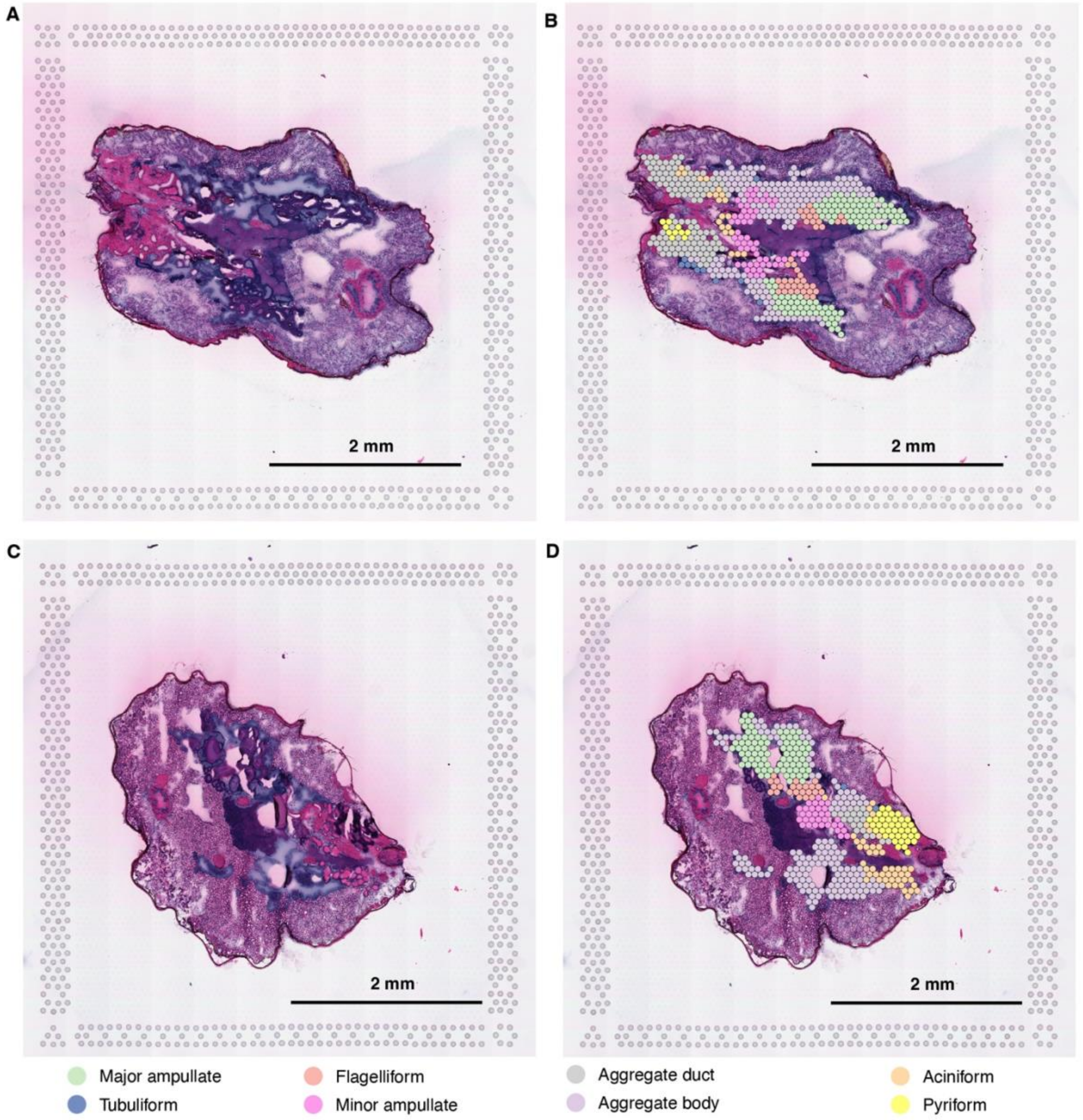

Fig. S12: Annotation of silk glands in the spider abdomen sections (2 and 3) used in Spatial transcriptomics.

Sections 2 **(A, B)** and 3 **(C, D)** used in spatial transcriptomics showing Hematoxylin-Eosin staining **(A, C)** and manual annotation of spots as different silk glands **(B, D)**. In (A) to (D), scale bar = 2 mm.

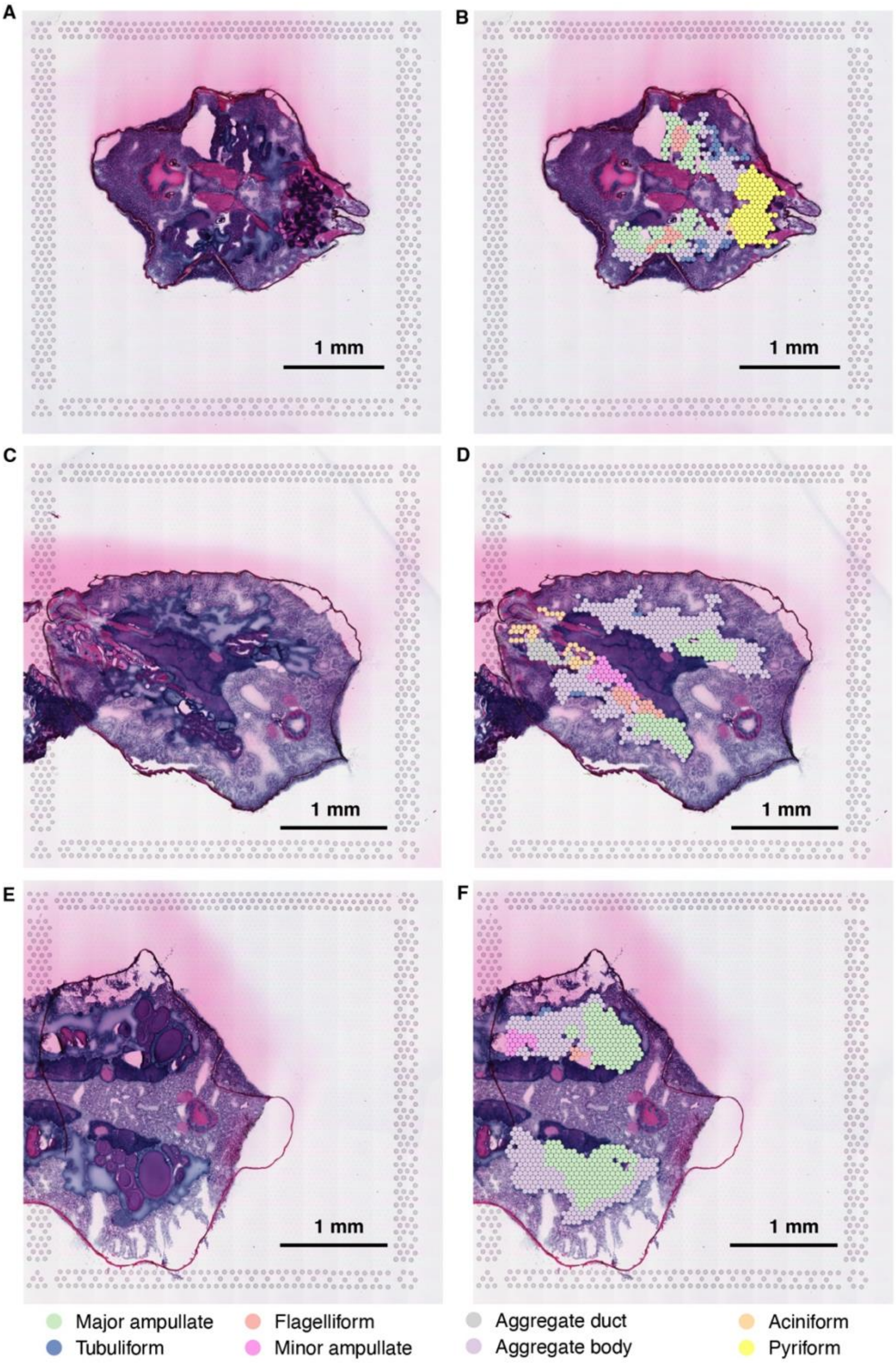

Fig. S13: Annotation of silk glands in the spider abdomen sections (4, 5 and 6) used in Spatial transcriptomics.

Sections 4 **(A, B)**, 5 **(C, D)** and 6 **(E, F)** used in spatial transcriptomics showing Hematoxylin-Eosin staining **(A, C** and **E)** and manual annotation of spots as different silk glands **(B, D** and **F)**. In (A) to (F), scale bar = 1 mm.

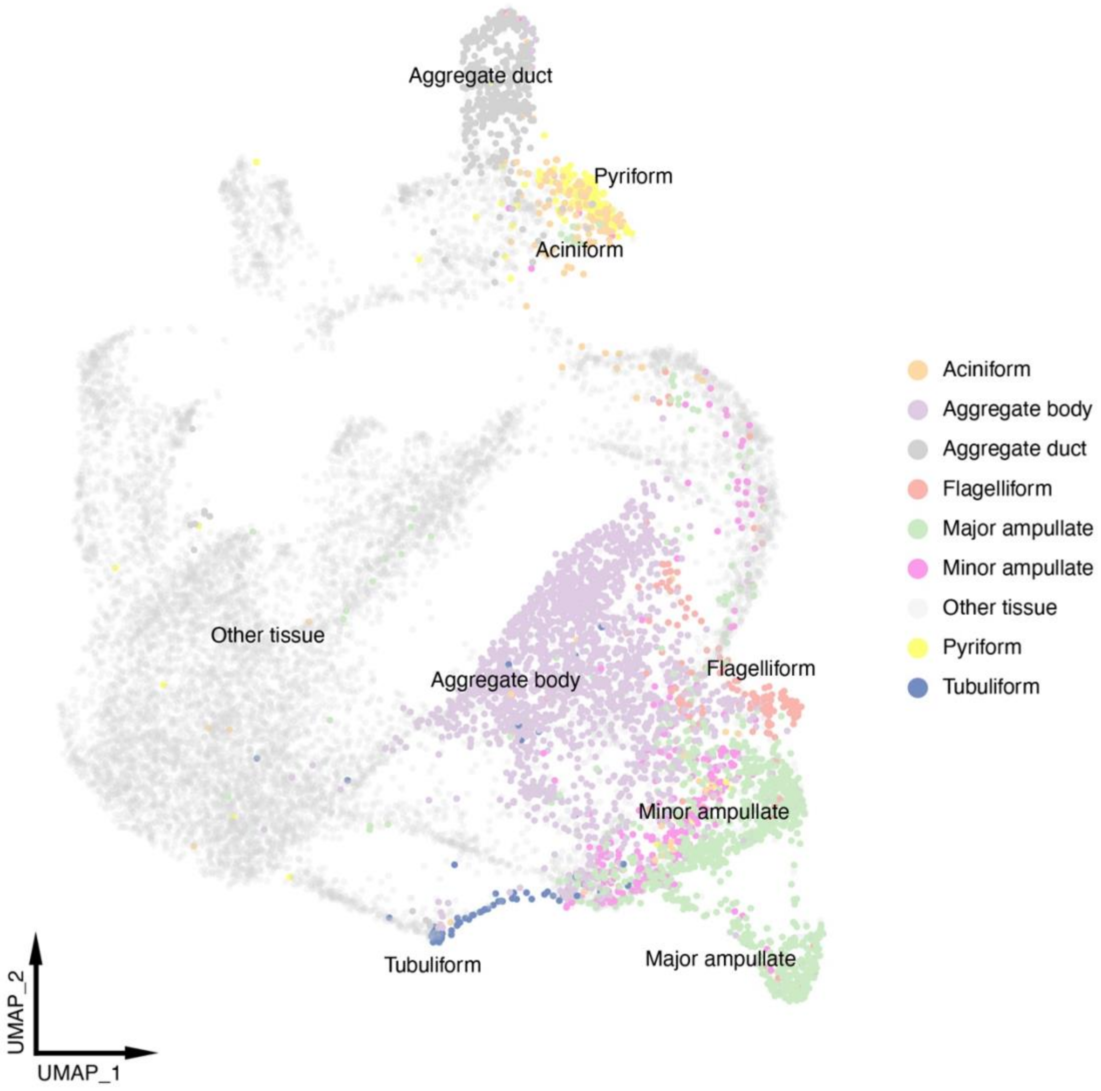

Fig. S14: Uniform manifold approximation and projection (UMAP) of all the spots from all sections used in spatial transcriptomics.

Each dot represents a spot on the spatial section. All spots from eight spatial sections were pooled and visualized using UMAP where the spots are colored according to the annotation. The axes (UMAP-1 and UMAP-2) represent the first and second UMAP dimensions, respectively. The spots annotated as silk glands separated from the spots of other tissues.

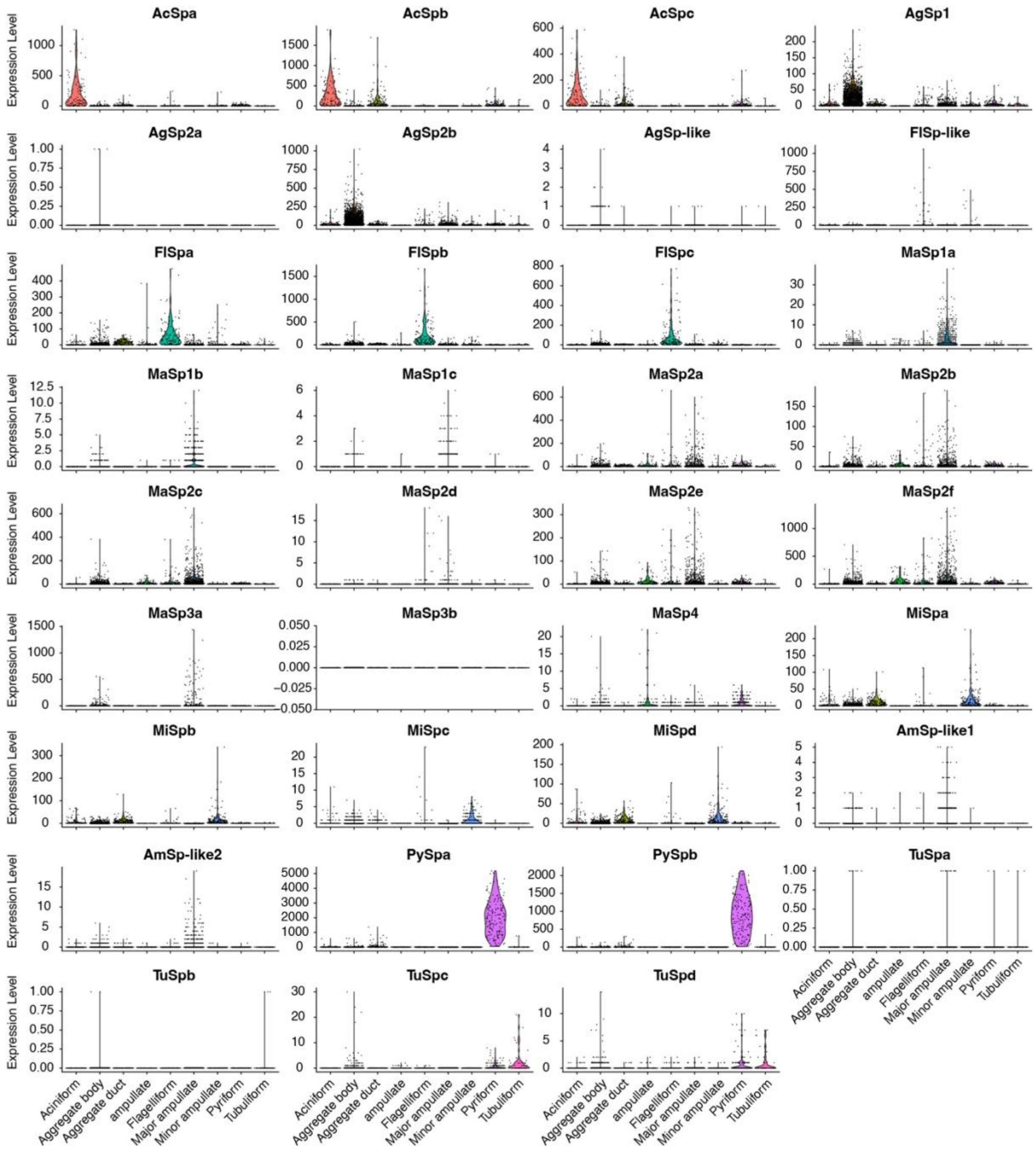

Fig. S15: Expression profiles of spidroin genes in the silk gland spots of spatial transcriptomic data.

Violin plots showing the expression of thirty-five spidroins identified in *L. sclopetarius* on the spatial spots annotated as silk glands. The silk gland type is indicated in the x-axis. Y-axis indicates expression levels. Each dot on the plots corresponds to a spot on the spatial sections. Ampullate cluster refers to spots which were identified as belonging to one of the ampullate glands but could not be distinguished further as major or minor ampullate glands.

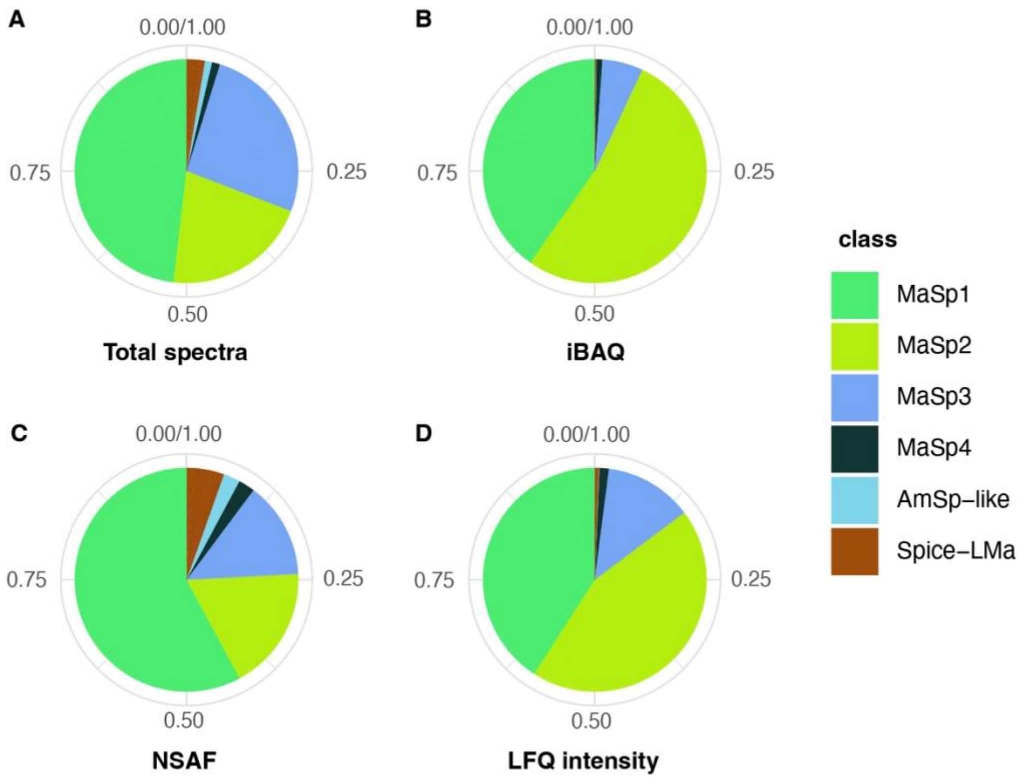

Fig. S16: Comparison of estimated protein composition in the silk fibers using different methods.

Estimation of relative abundance of silk proteins in the major ampullate fiber dissolved in HFIP using **(A)** number of total spectra, **(B)** intensity Based Absolute Quantification (iBAQ), **(C)** Normalized Spectral Abundance Factor (NSAF), and **(D)** Label free quantification (LFQ) intensity. Three biological replicates were used for this analysis. Total spectra (A) and NSAF (C) use spectral counts whereas iBAQ (B) and LFQ intensity (D) use intensity of peaks to estimate relative protein abundances. Colors indicate protein types.

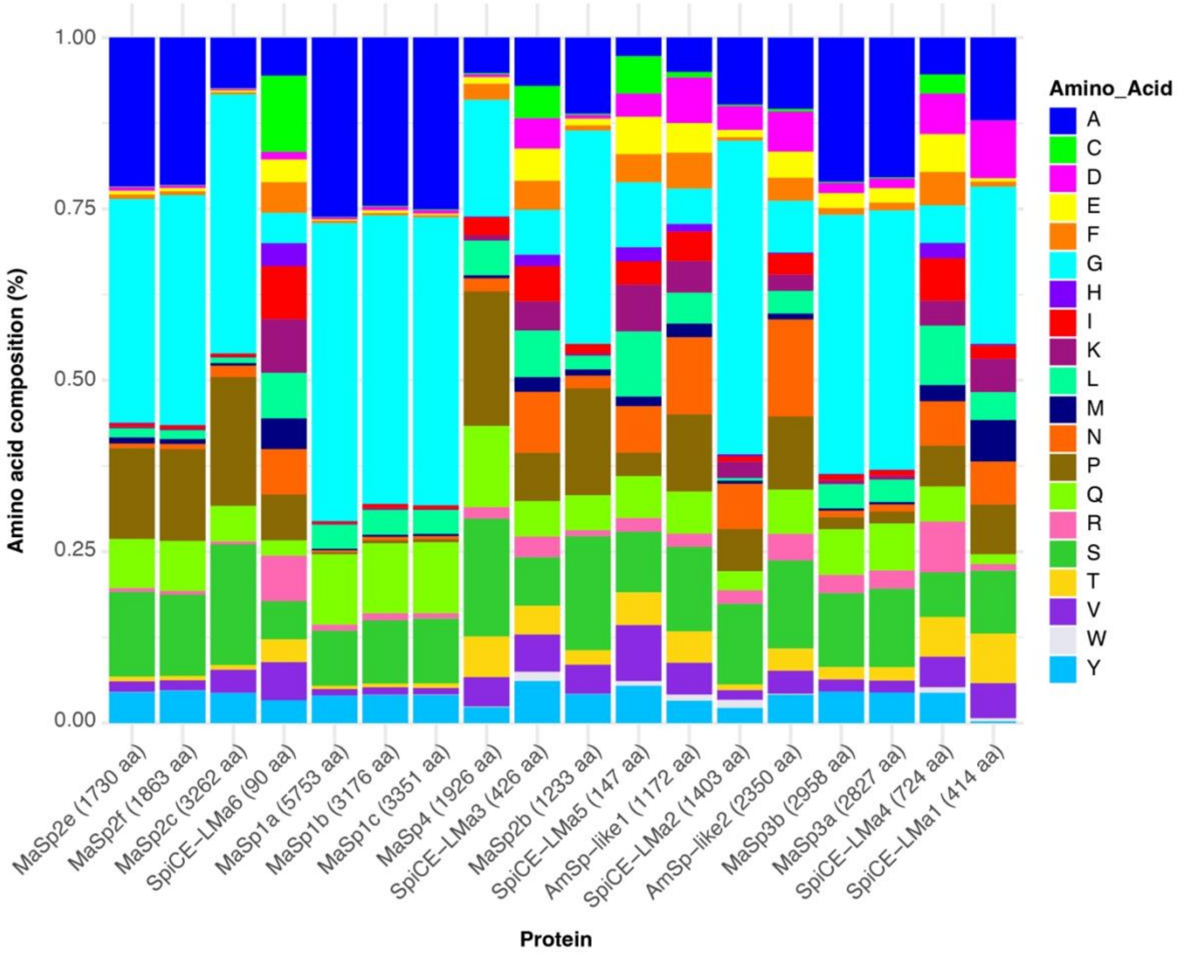

Fig. S17: Amino acid residue composition of the 18 silk proteins.

Fraction of amino acid residue content in the 18 silk proteins determined using ProtParam tool. The colors indicate the amino acid residues denoted as single letter codes in the legend.

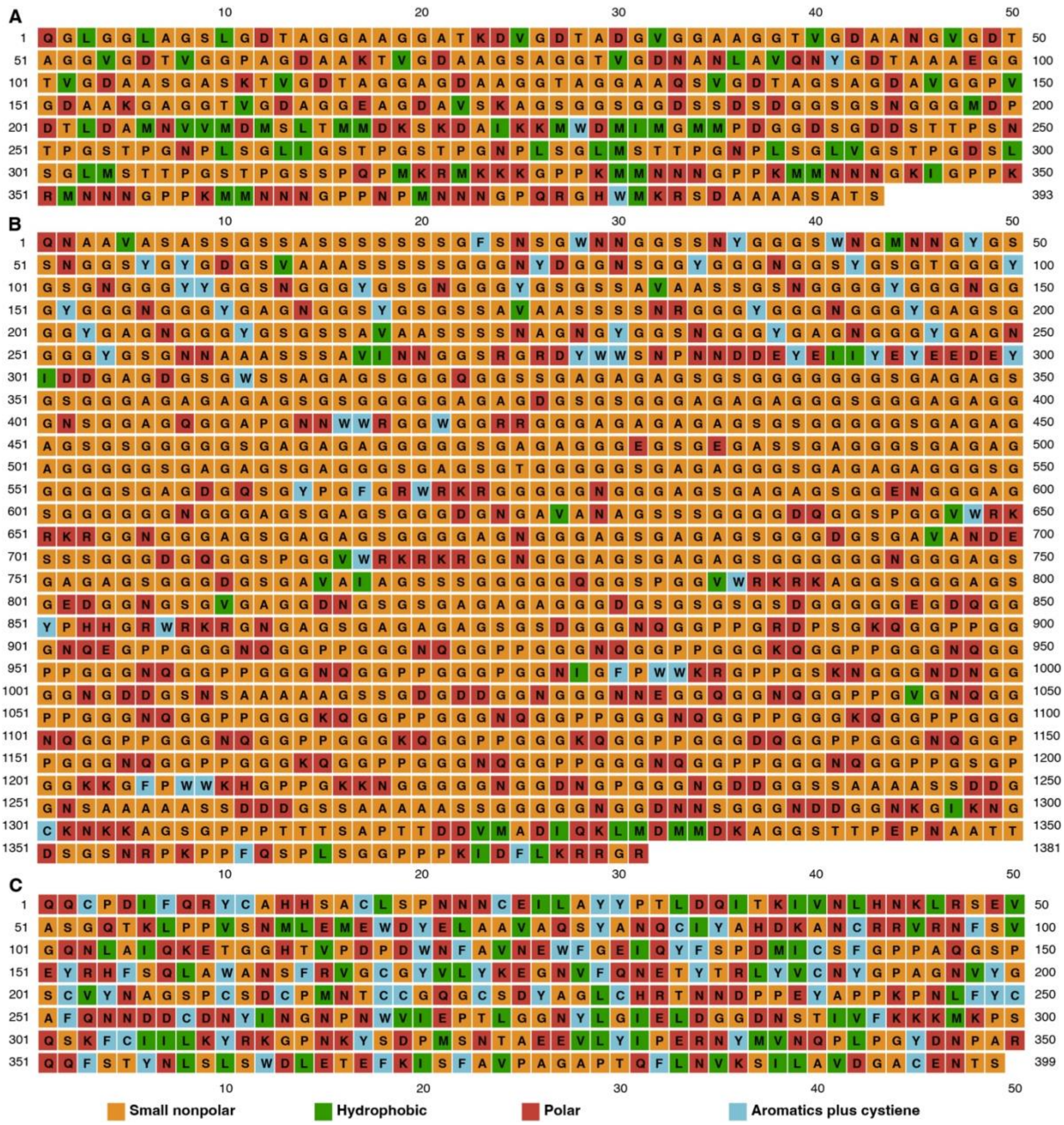

Fig. S18: Amino acid residue types in SpiCE-LMa1–3.

(A) SpiCE-LMa1. **(B)** SpiCE-LMa2. **(C)** SpiCE-LMa3. In all, the amino acid residues are colored according to the category they belong to (small nonpolar: A, G, P, S, T; hydrophobic: I, L, M, V; polar: D, E, H, K, N, Q, R; aromatic and cysteine: C, F, W, Y)

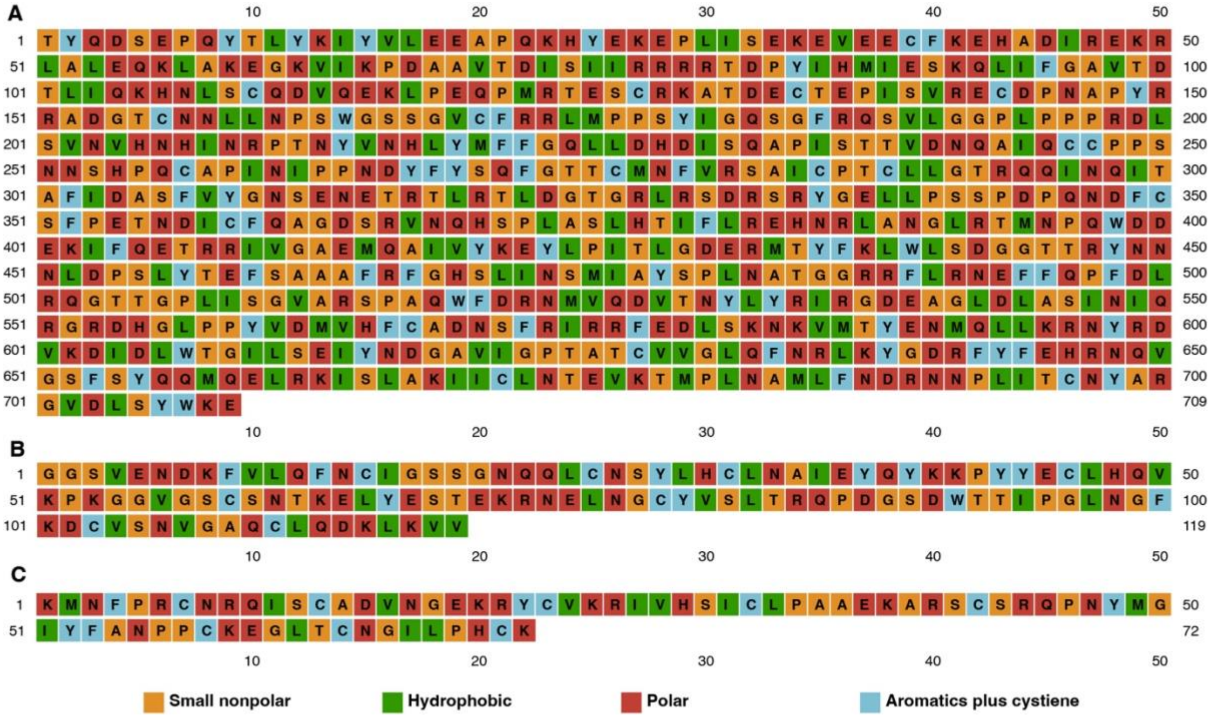

Fig. S19: Amino acid residue types in SpiCE-LMa4–6.

(A) SpiCE-LMa4. **(B)** SpiCE-LMa5. **(C)** SpiCE-LMa6. The amino acid residues are colored according to the category they belong to (small nonpolar: A, G, P, S, T; hydrophobic: I, L, M, V; polar: D, E, H, K, N, Q, R; aromatic and cysteine: C, F, W, Y)

Figures S20 to S25 show secondary structural predictions for SpiCE-LMa1–6 proteins using PSIPRED4.0 protein structure prediction server (*91*). The colors correspond to protein secondary structure elements (yellow for β-strands, pink for α-helix and grey for coil structures). Confidence of prediction is shown as shades of blue.

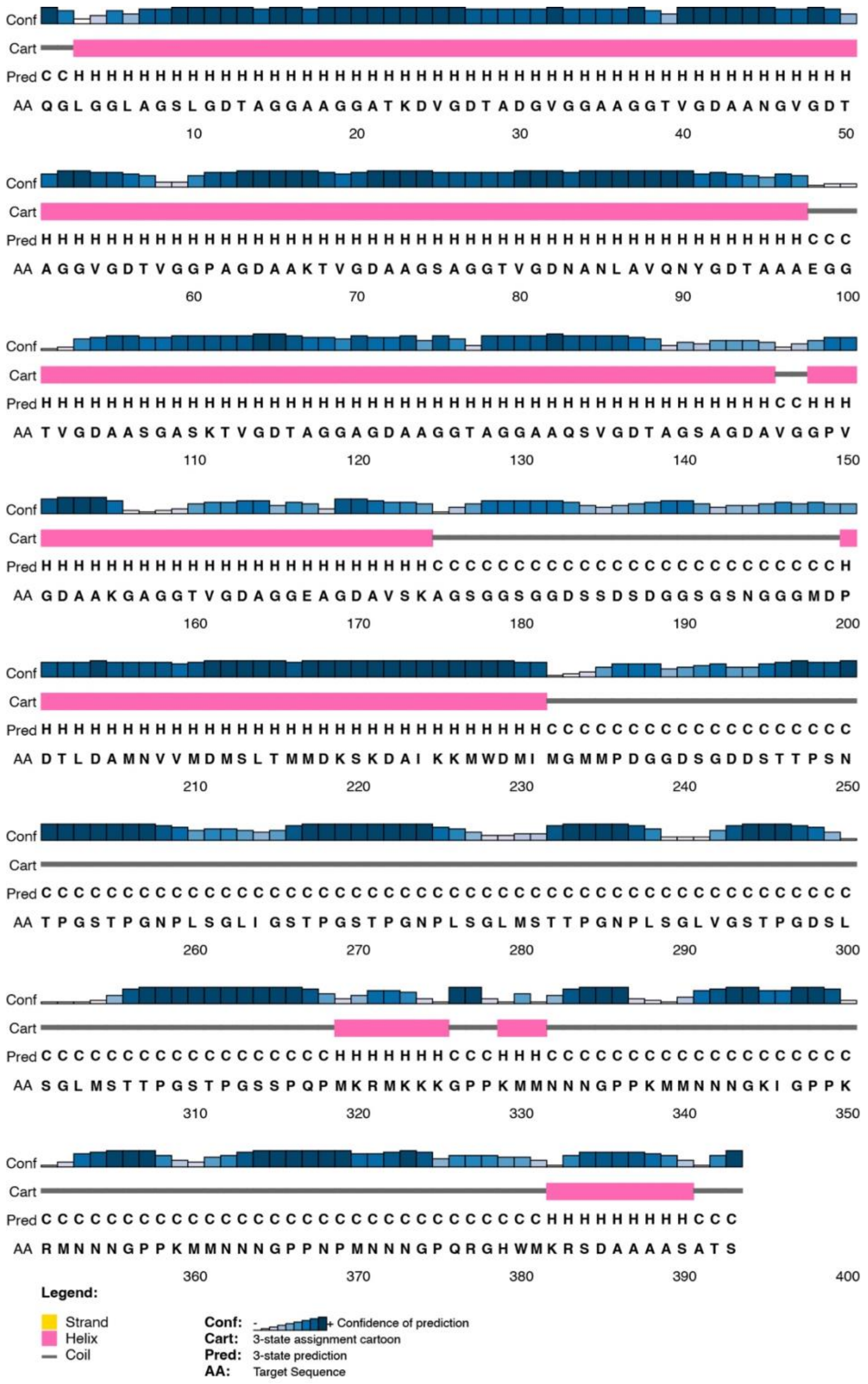

Fig. S20: Secondary structure prediction for SpiCE-LMa1.

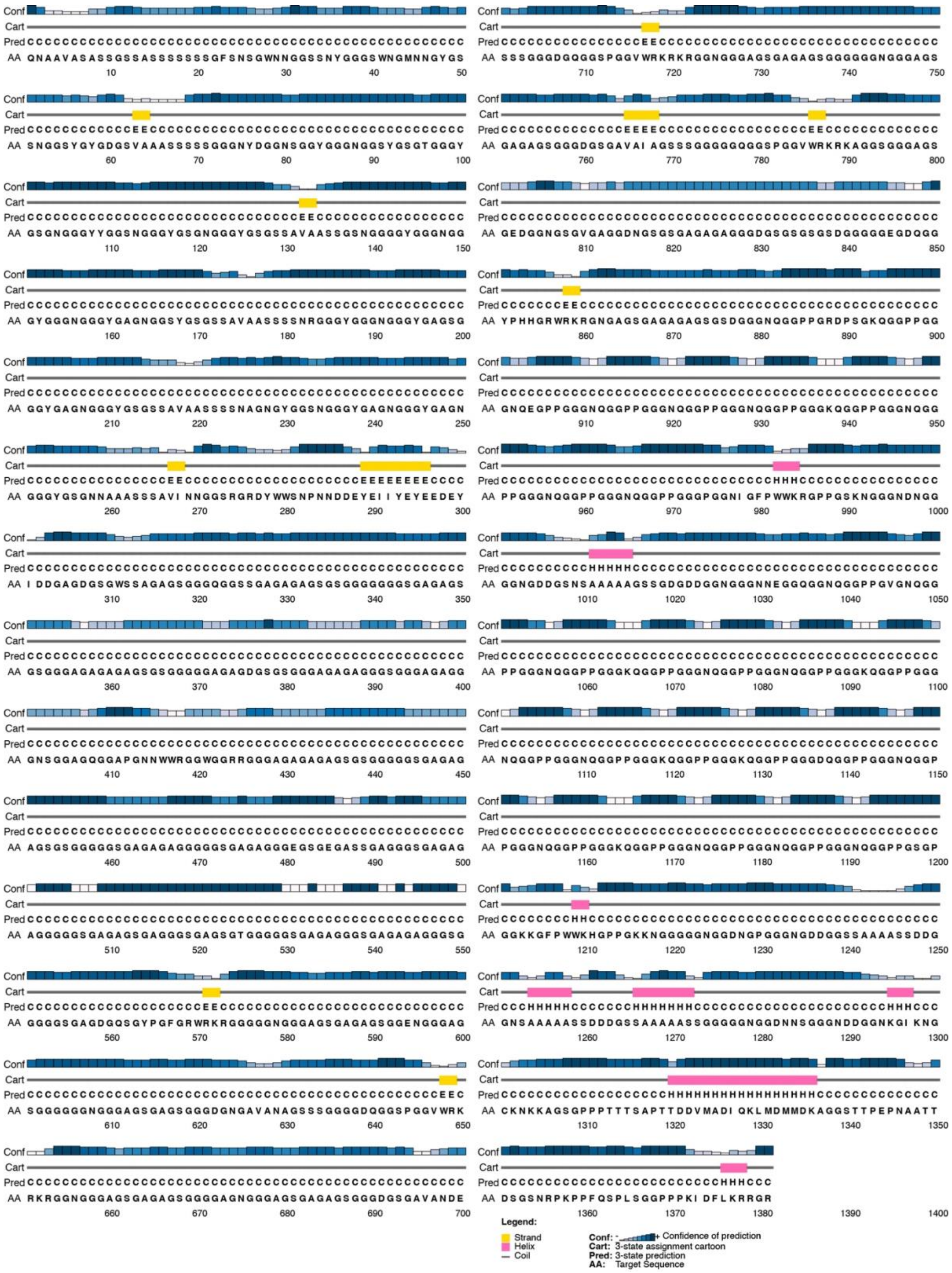

Fig. S21: Secondary structure prediction for SpiCE-LMa2.

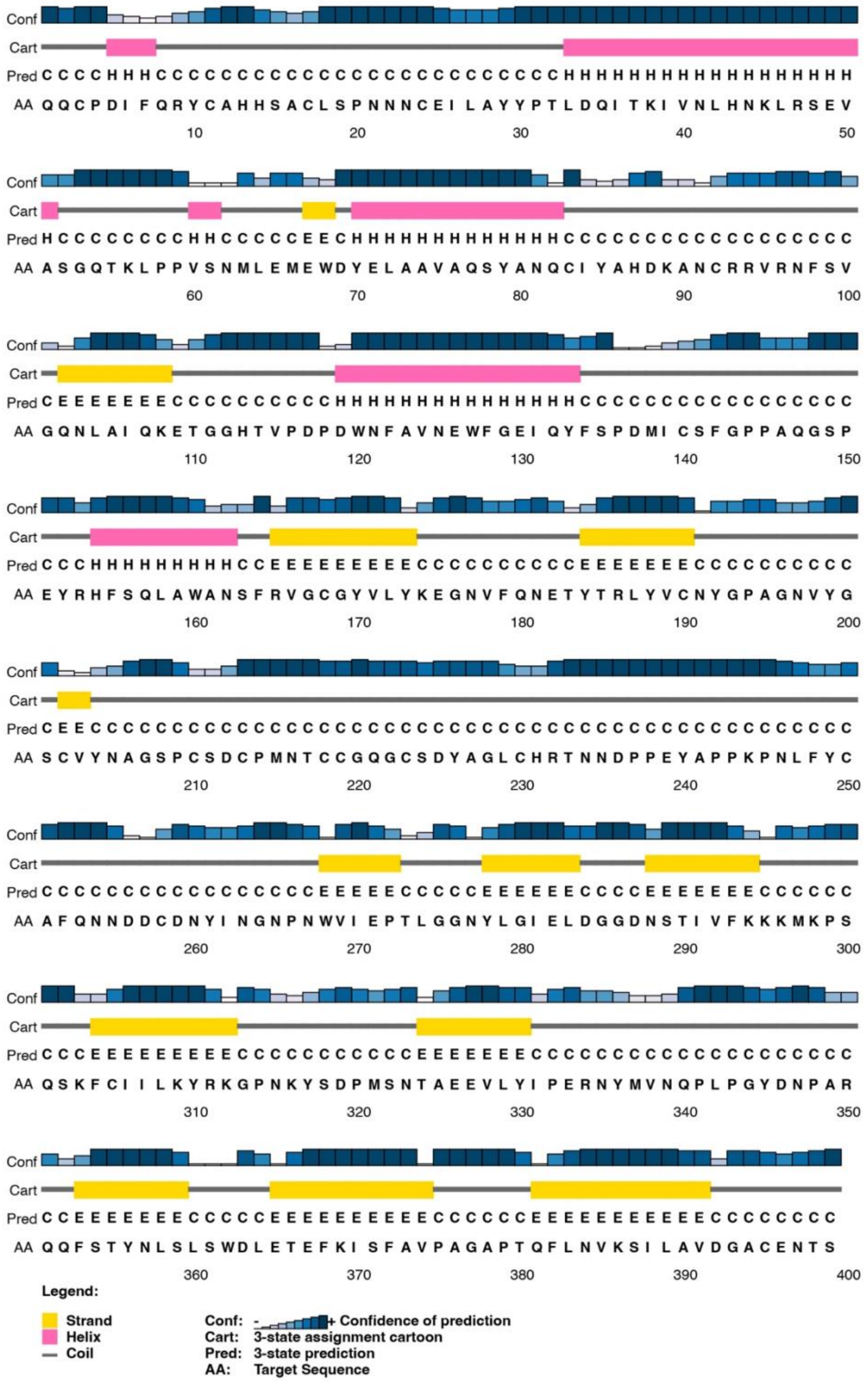

Fig. S22: Secondary structure prediction for SpiCE-LMa3.

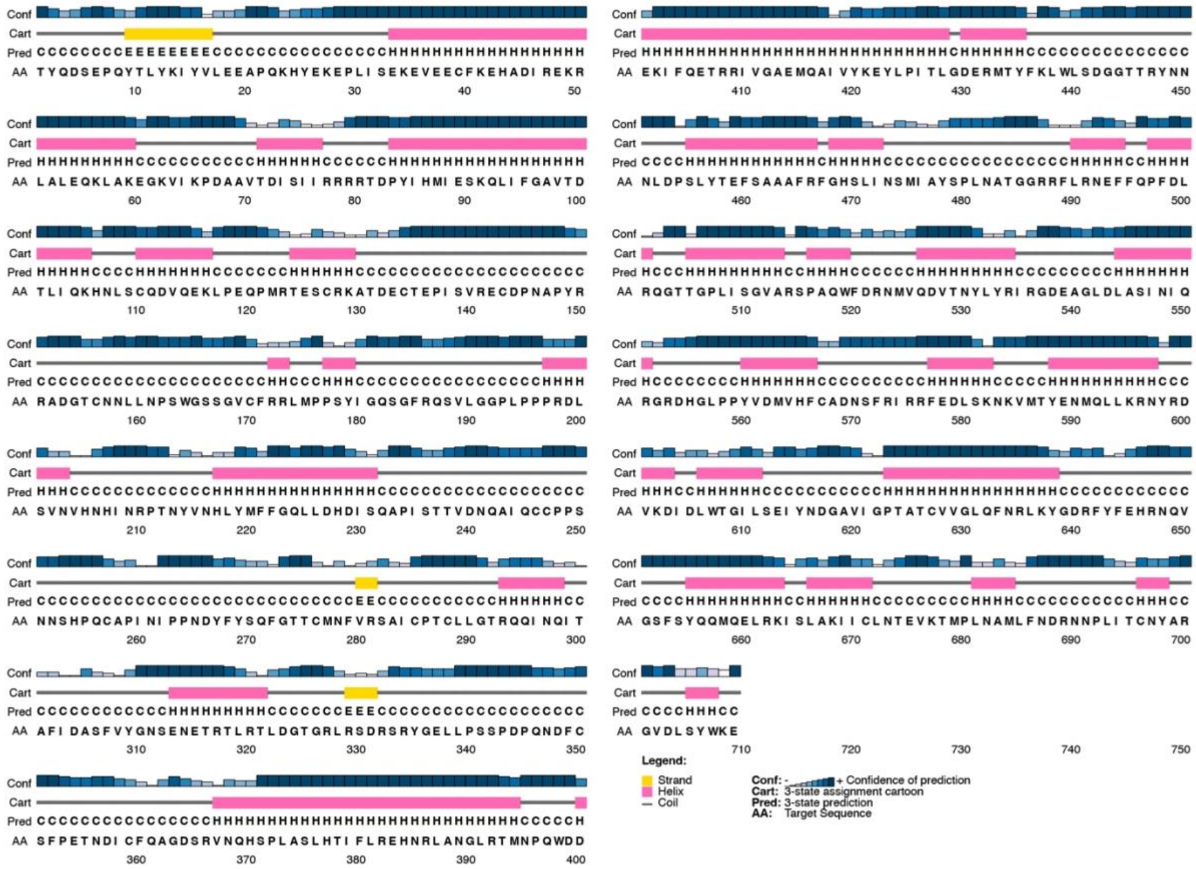

Fig. S23: Secondary structure prediction for SpiCE-LMa4.

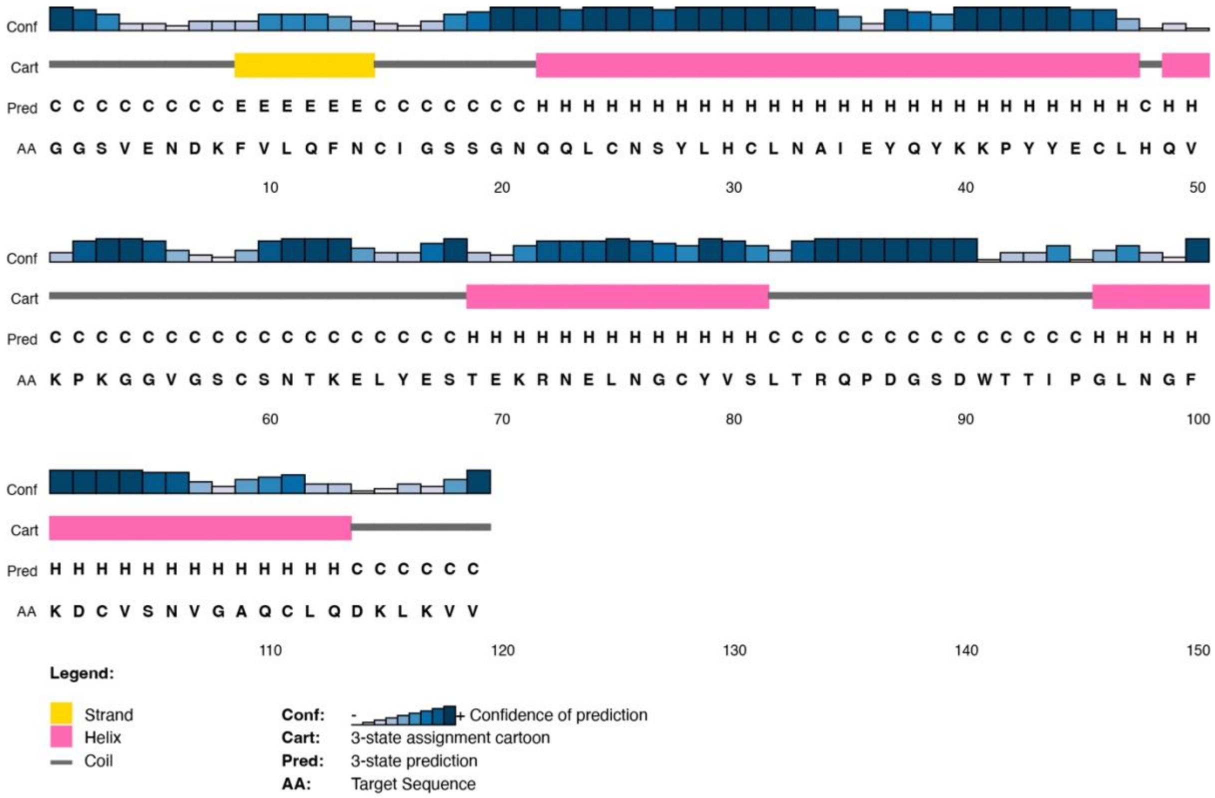

Fig. S24: Secondary structure prediction for SpiCE-LMa5.

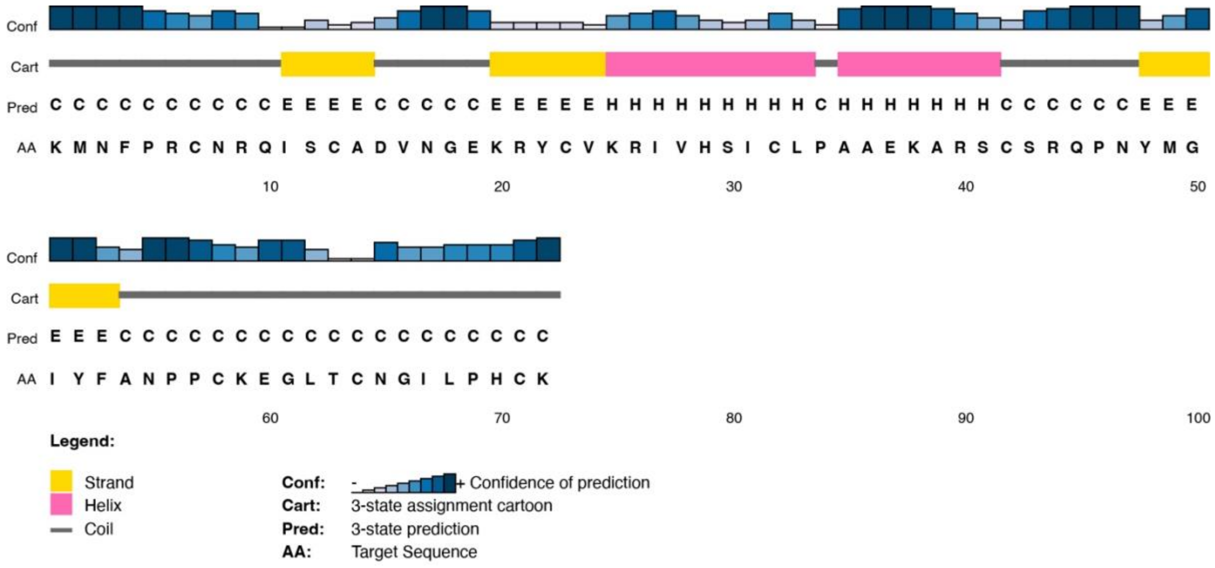

Fig. S25: Secondary structure prediction for SpiCE-LMa6.

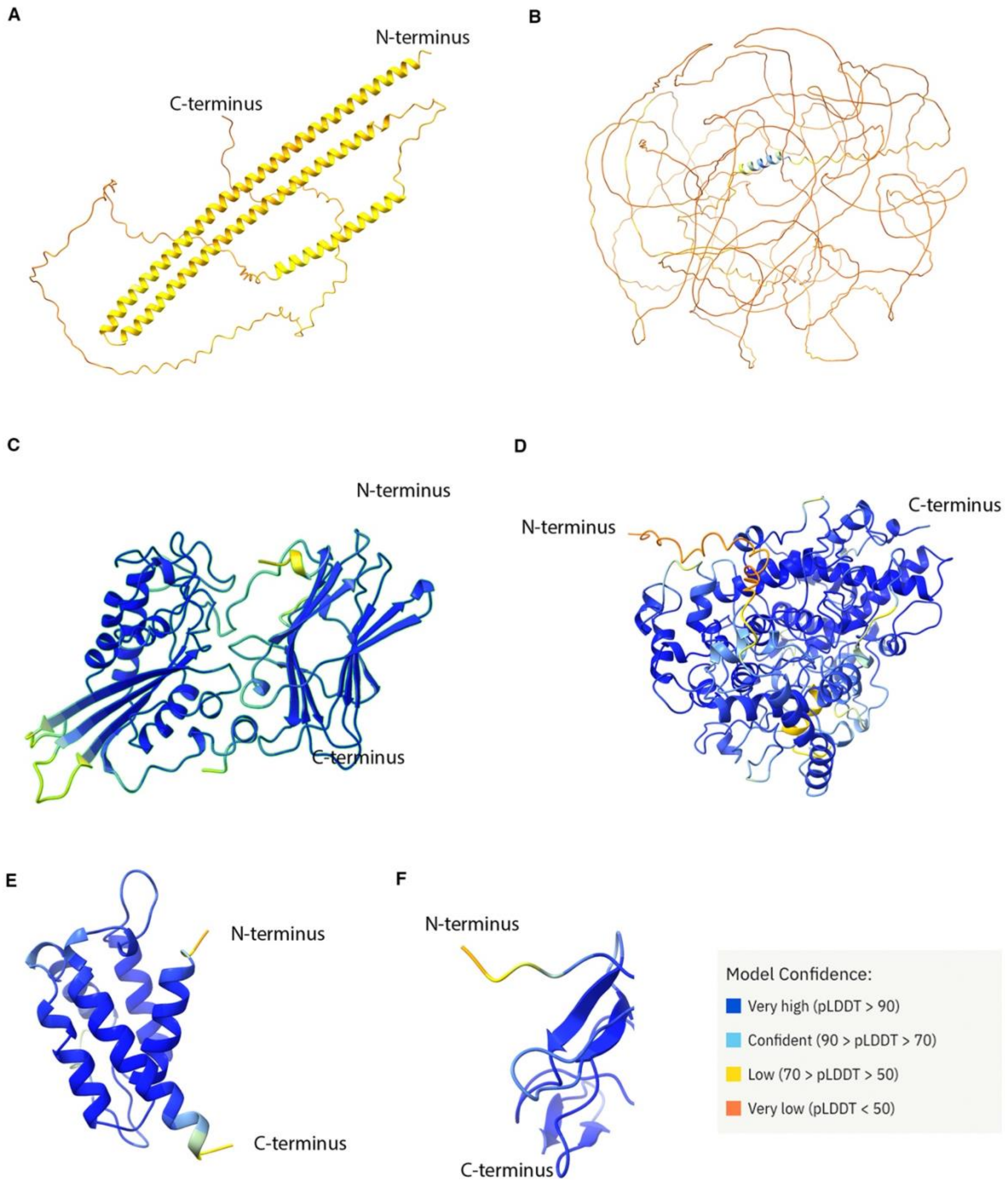

Fig. S26: Tertiary structure prediction for SpiCE-LMa proteins.
(A) SpiCE-LMa1. **(B)** SpiCE-LMa2. **(C)** SpiCE-LMa3. **(D)** SpiCE-LMa4. **(E)** SpiCE-LMa5. **(F)** SpiCE-LMa6. The structure predictions were performed using AlphaFold (*42*) tool in ChimeraX (v1.6) with default parameters. Colors indicate per-residue confidence scores (0-100) determined using predicted local distance difference test (pLDDT). Residues with scores greater than 90 are colored as blue, between 90 and 70 as light blue, between 70 and 50 as yellow and below 50 as orange (see legend). The predicted structures likely relate to the soluble form of the proteins.

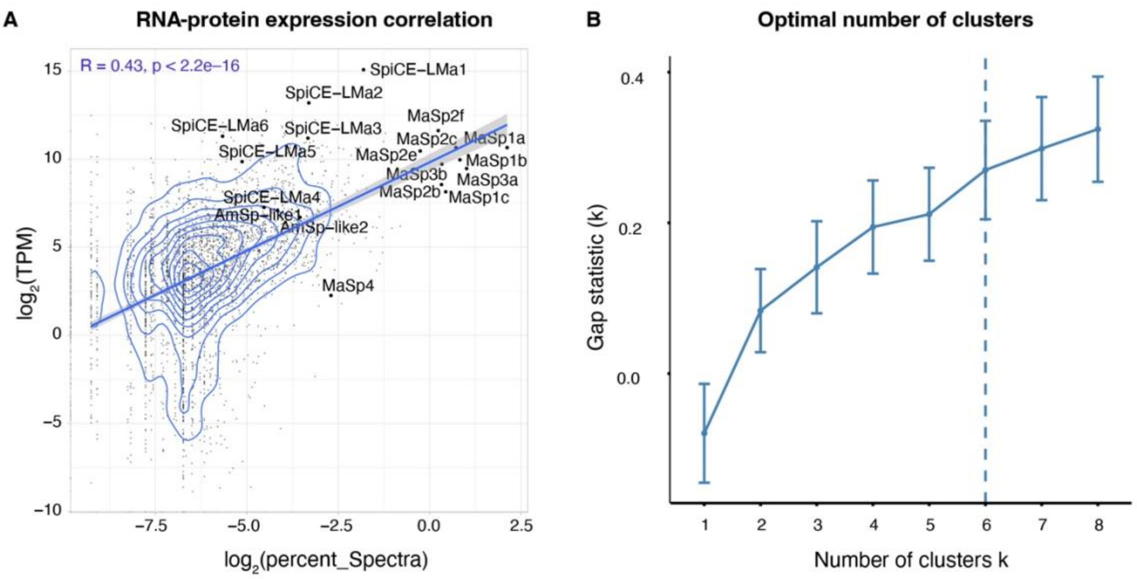

Fig. S27: Bulk RNA expression correlation and clustering.

(A) Pearson correlation of the RNA expression (TPM values) and protein levels (percent of total spectra) of all 3985 proteins identified in the major ampullate gland. The 18 silk genes/proteins are indicated. **(B)** Hierarchical cluster analysis to identify optimal number of clusters based on expression profiles from 17 samples including tail (6), sac (6) and duct (5). Heatmap of samples and hierarchical tree is shown in Figure 2.

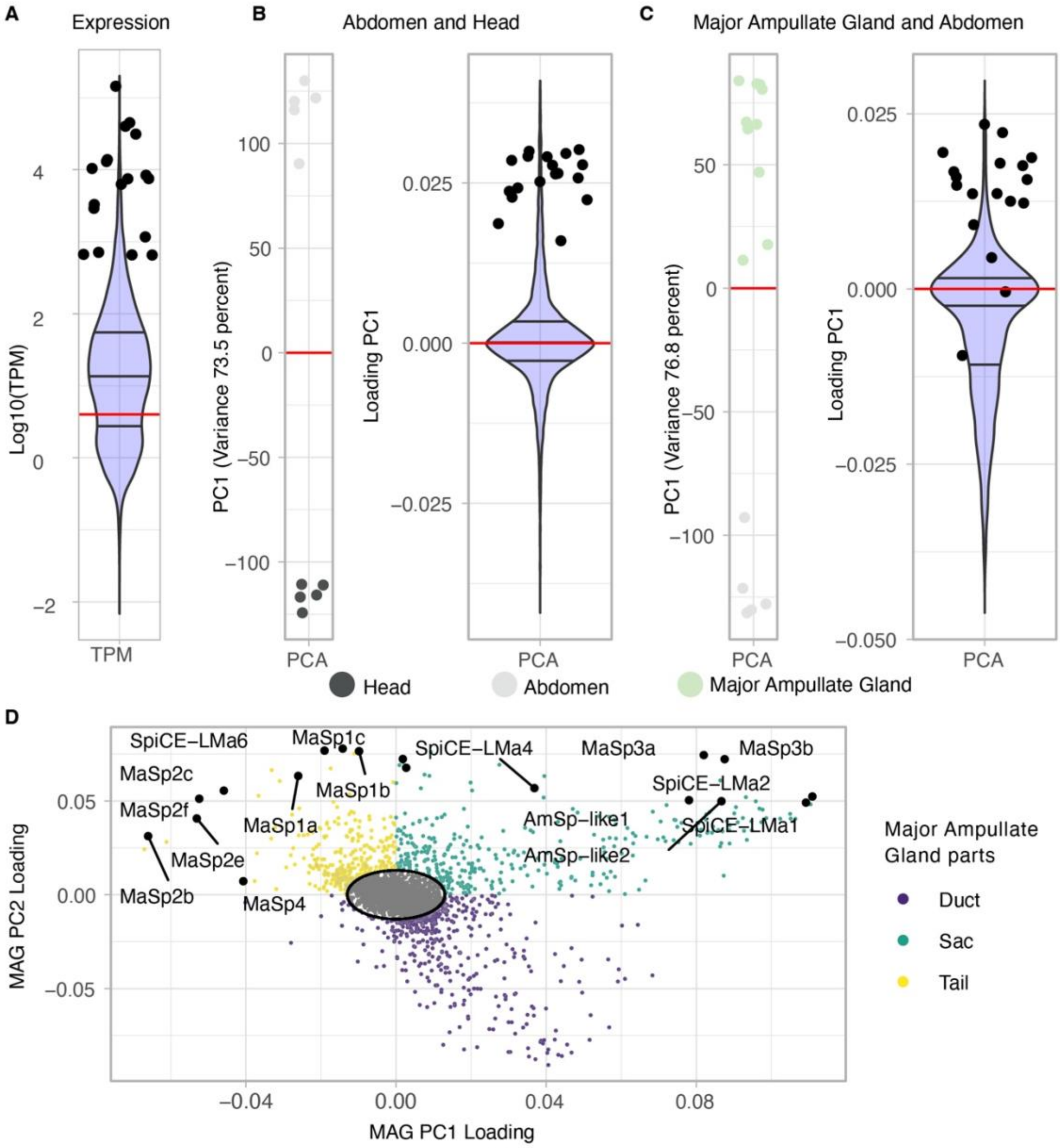

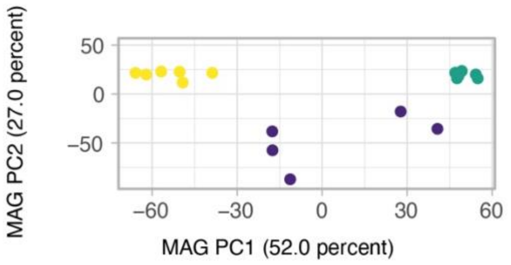

E

Step	Filter	Cutoff	Count
0	None		22 860
1	A	Max(TPM) > 4	15 086
2	B	Loading PC1 > 0	7777
3	C	Loading PC1 >0	2776
4	D	$(PC1^2+PC2^2)^{1/2} > 0.13$	1048

Fig. S28: Major ampullate specific genes.

(A) Distribution of lowest $\log_{10}$(TPM) per gene for 22860 genes in all samples used for bulk RNA sequencing. Red line indicates cutoff (TPM=4). All genes with TPM <4 were removed. **(B)** PCA analysis of abdomen and head samples for 15086 genes. Left panel shows sample distribution in 1st PC covering 73.5% of all variation in the samples. Right panel shows PCA loading for 15086 genes. Genes with negative loading were removed. **(C)** PCA analysis of abdomen and major ampullate gland samples for 7777 genes. Left panel shows sample distribution in 1st PC covering 76.8% of all variation in the samples. Right panel shows PCA loading for 7777 genes. Genes with negative loading were removed. **(D)** PCA analysis of

samples from the three parts (tail, sac, and duct) of the major ampullate gland (MAG) for 2776 genes. Top panel shows PCA loading for 2776 genes. Genes with Euclidian distance <0.13 from origin (indicated by a black ellipse) were removed resulting in 1048 genes which were designated major ampullate-specific genes. Bottom panel shows sample distribution in 1st and 2nd PCs covering 79% of all variation in the samples. In (A) to (D), black dots indicate the 18 silk genes **(E)** Table summarizing the reduction of genes from steps A to D.

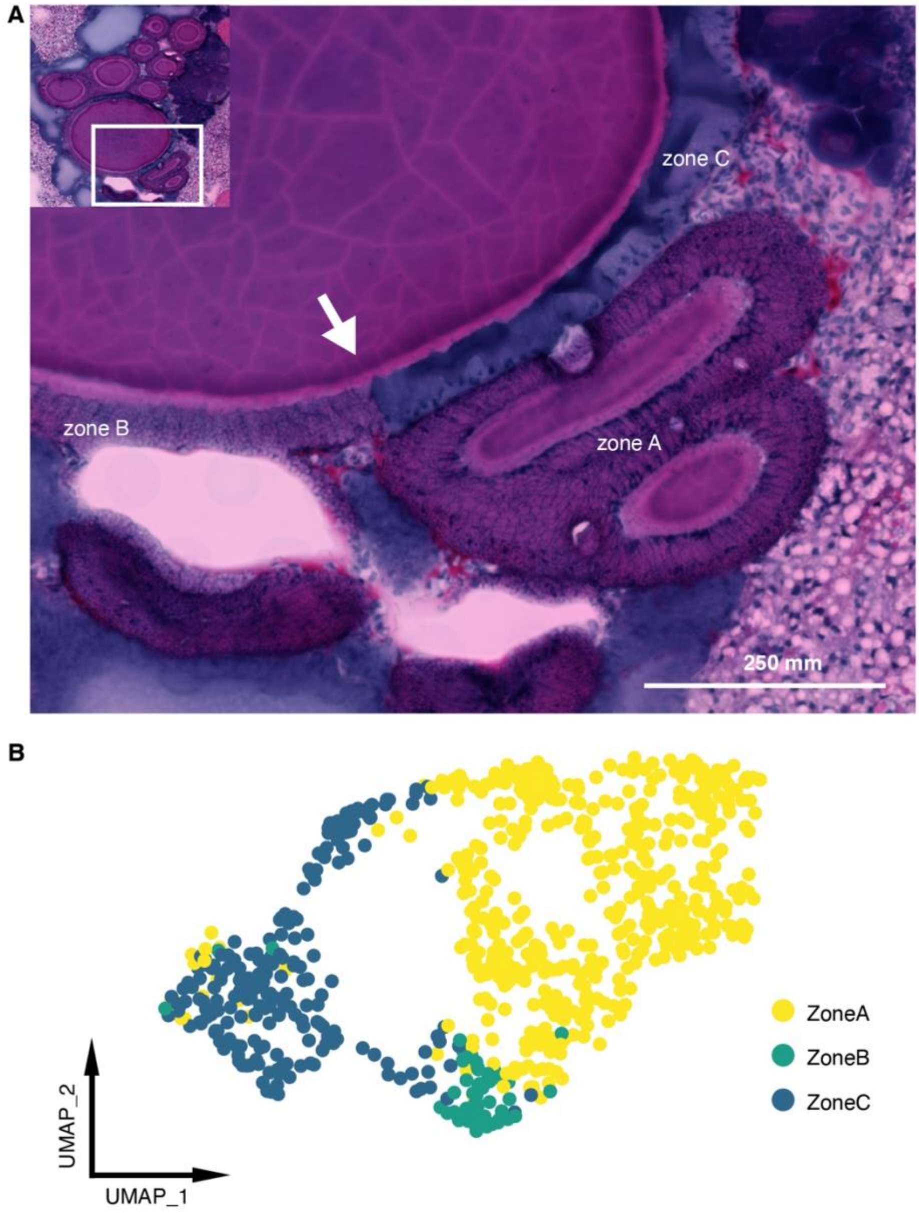

Fig. S29: Major ampullate zones on spatial section.
(A) High magnification HE-stained image showing the morphology of zones in one of the major ampullate glands (top left inset) on spatial section 4, arrow indicates the sharp transition between zone B and C. **(B)** The spots annotated as major ampullate glands were further annotated as zone A, B, and C. All such spots from different sections were pooled together and visualized in the UMAP, colors indicate different zones. Each dot on the UMAP represents a spot on the section.

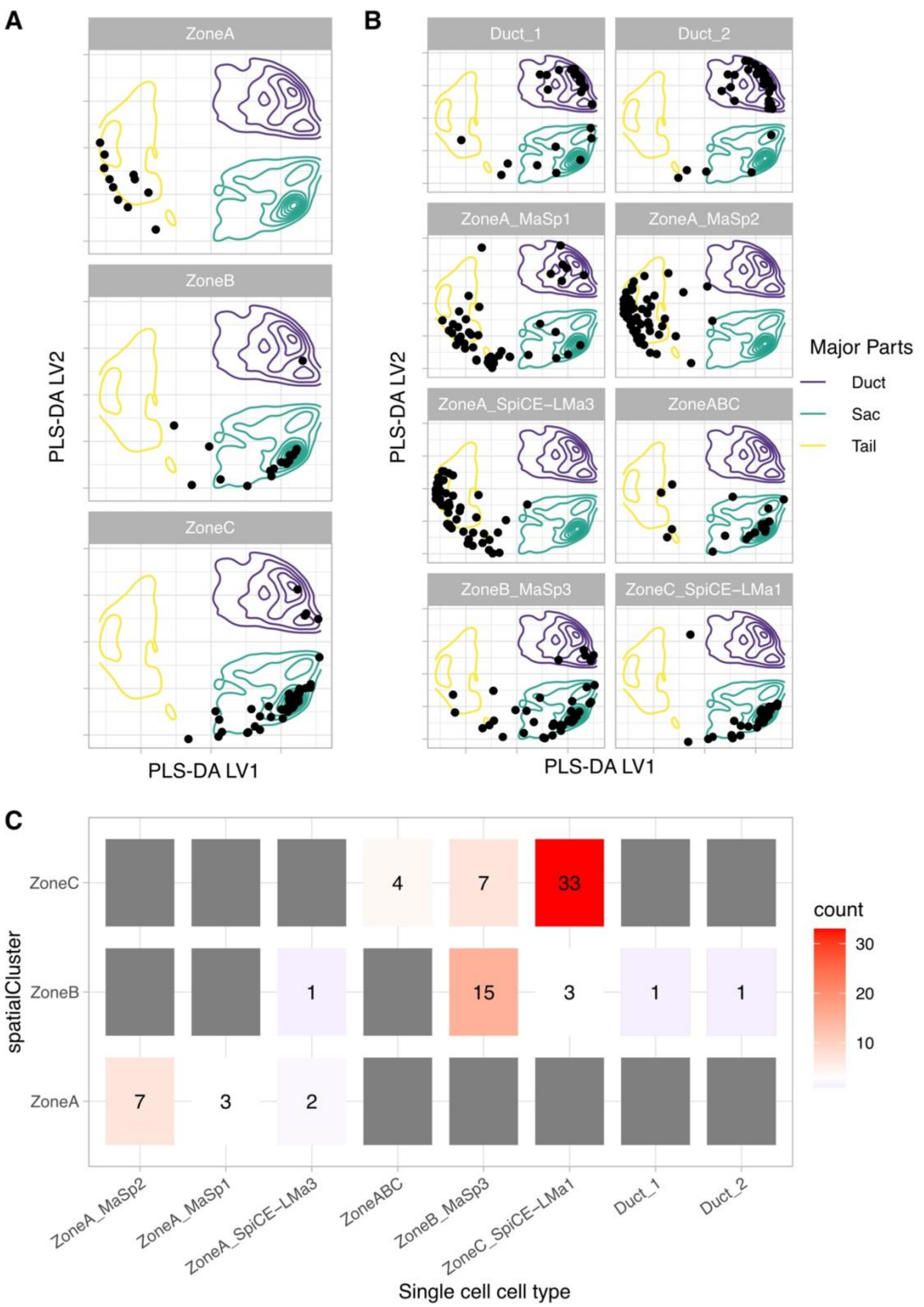

Fig. S30: Location of marker genes of clusters obtained from single-cell and spatial transcriptomic data on PLS plots obtained from bulkRNA data.

Loading of genes mapped onto the first two PLS dimensions that separates the bulk RNA samples tail, sac, and duct. The distribution of the loading for tail, sac, and duct are visualized with 2D density plot. **(A)** Overlap of top marker genes for the three major ampullate zones

obtained from spatial transcriptomics with the PLS plots of tail, sac, and duct bulk RNA. **(B)** Overlap of top marker genes from single-cell RNA clusters with the PLS plots of tail, sac, and duct bulk RNA. In (A) and (B), the tail, sac and duct (yellow, green, blue, respectively) are represented by the 2D density plots, and the marker genes are shown as black dots. **(C)** Heatmap showing gene overlap between top 50 marker genes from single-cell clusters and top 50 marker genes from spatial transcriptomic data.

Figures S31 toS35 show the expression profiles of the 18 silk genes in the major ampullate glands on the spatial sections. The major ampullate zones and gene counts are indicated. The scale of expression for each gene goes from blue (count = 0) to red (count = max), shades of yellow indicate intermediate count values.

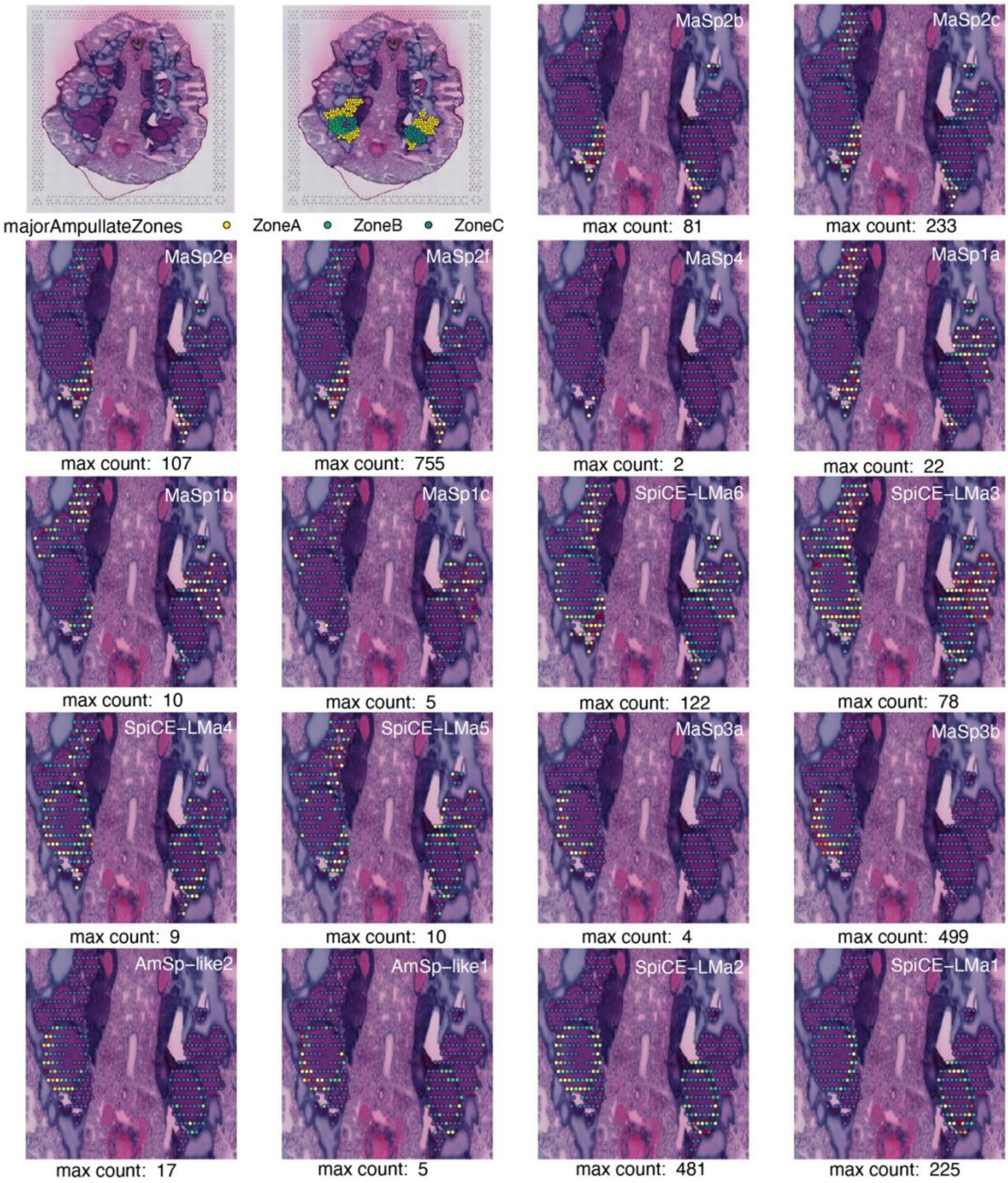

Fig. S31: Expression of silk genes in major ampullate glands on the spatial section 1.

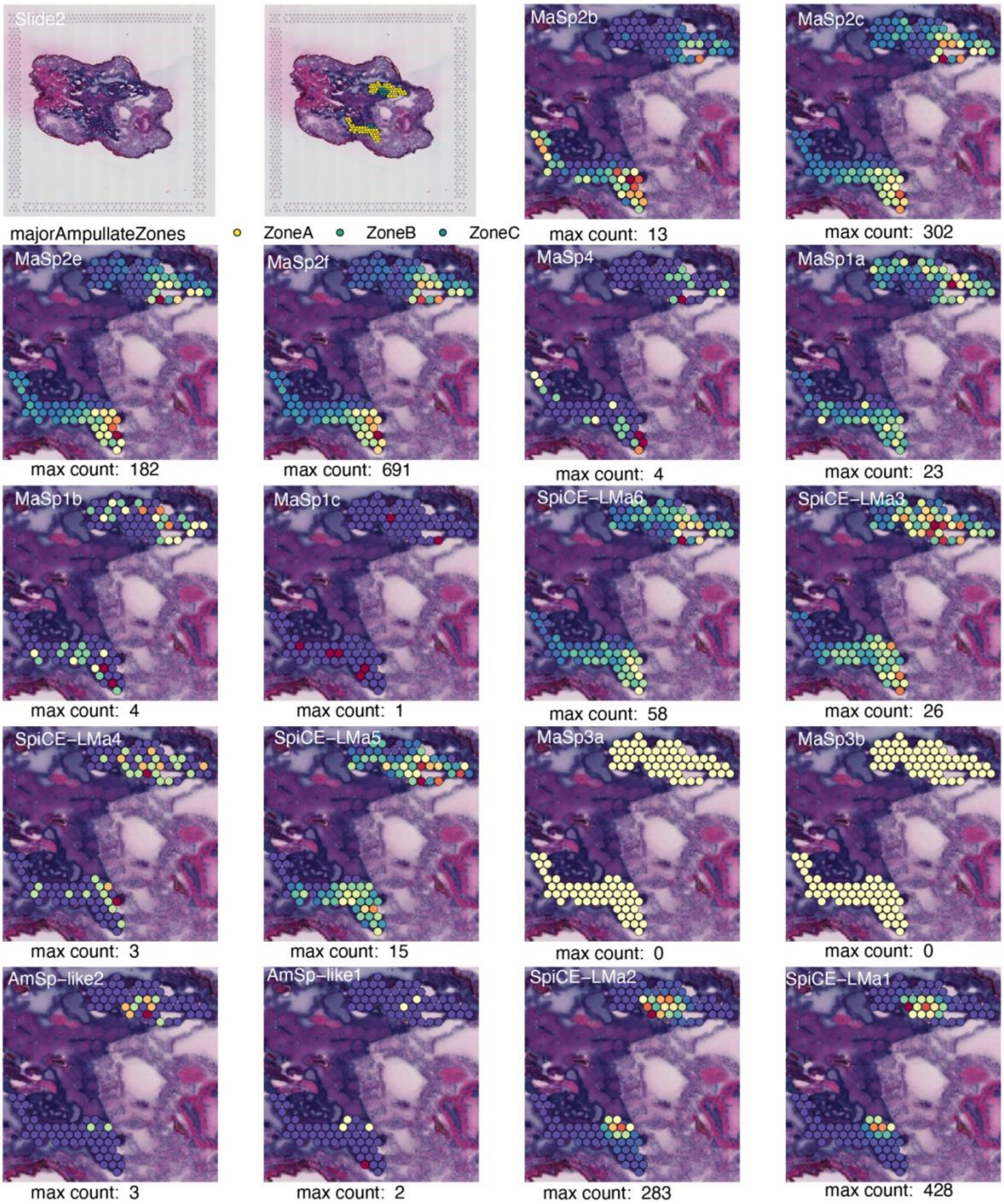

Fig. S32: Expression of silk genes in major ampullate glands on the spatial section 2.

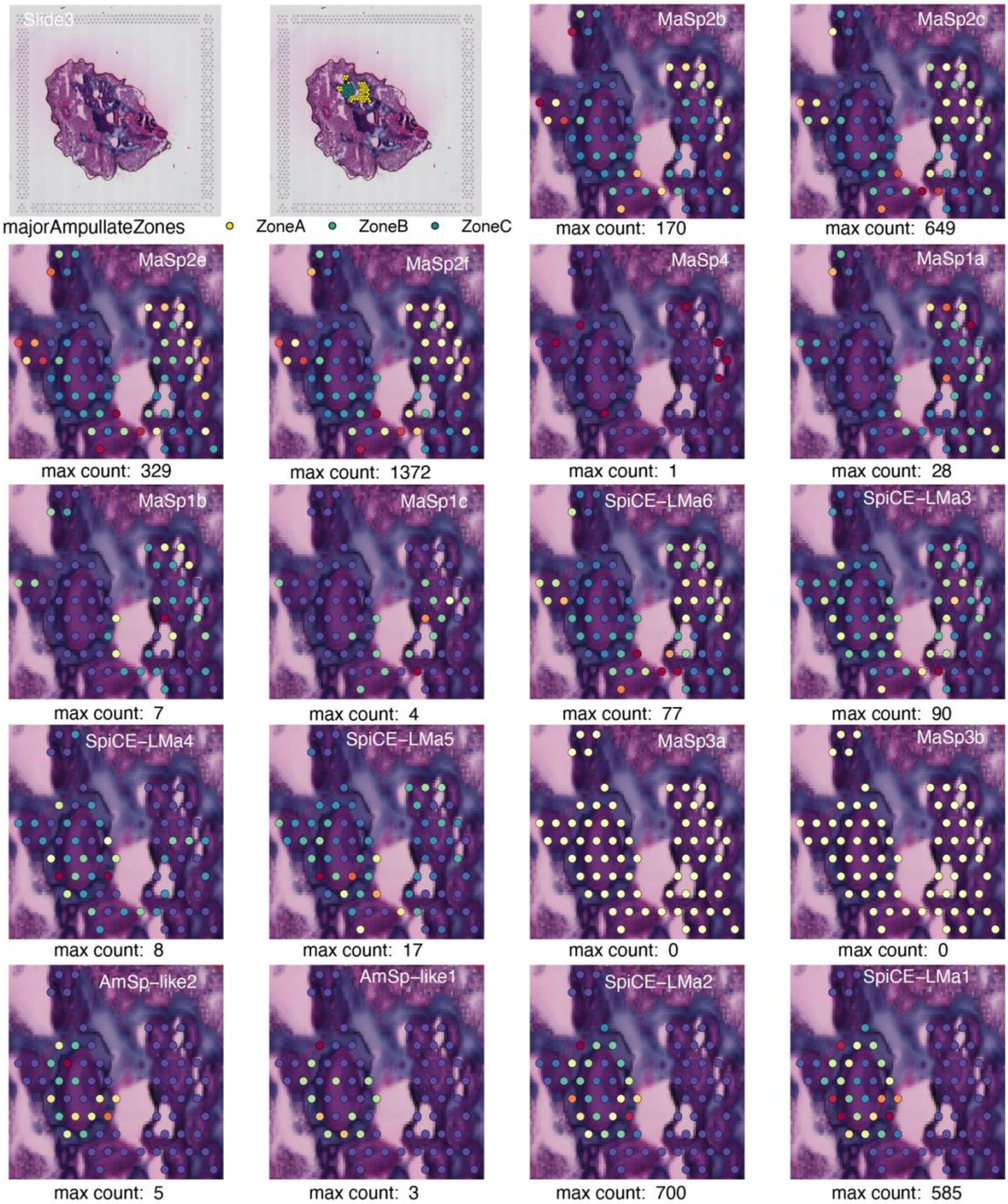

Fig. S33: Expression of silk genes in major ampullate glands on the spatial section 3.

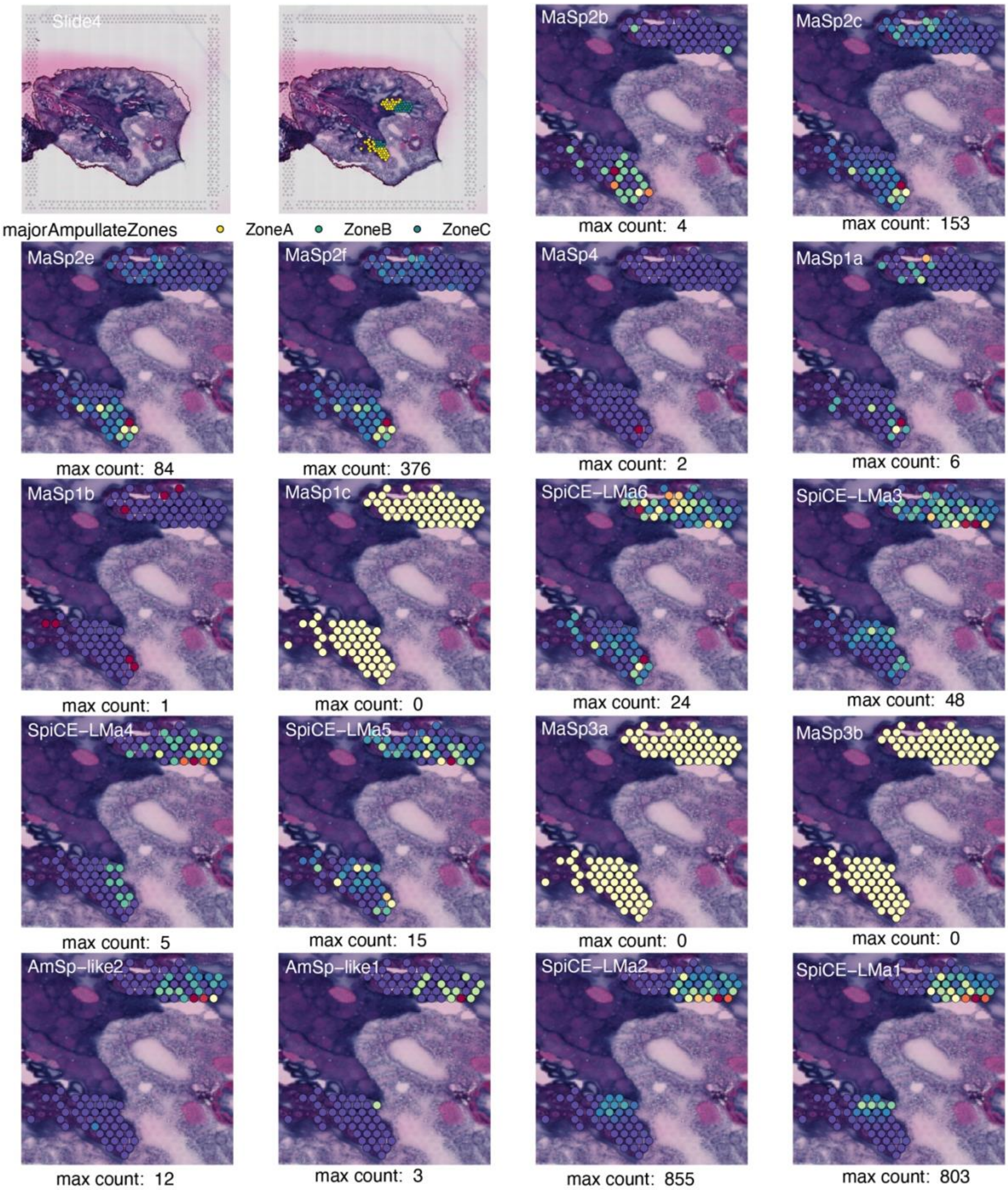

Fig. S34: Expression of silk genes in major ampullate glands on the spatial section 4.

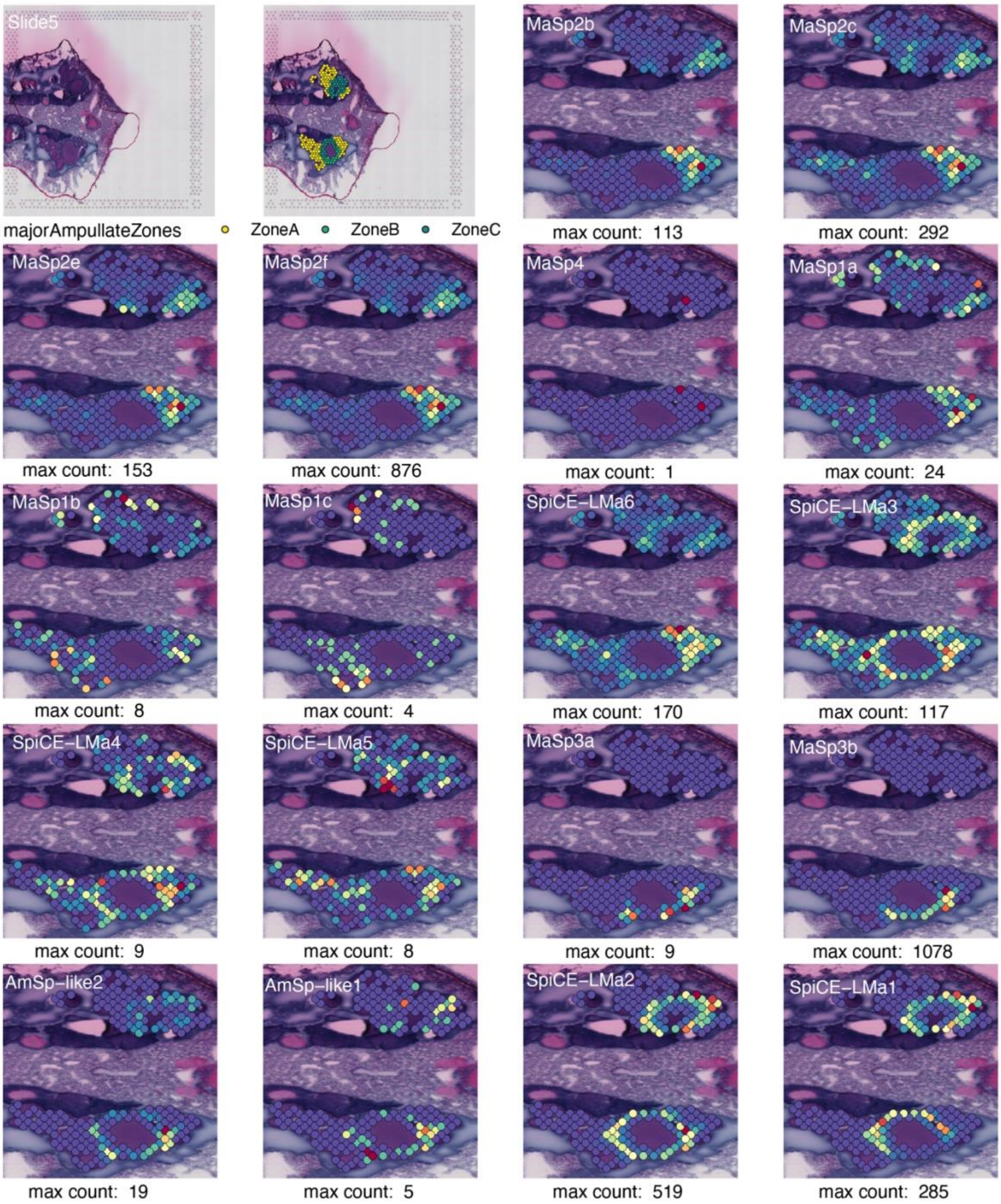

Fig. S35: Expression of silk genes in major ampullate glands on the spatial section 5.

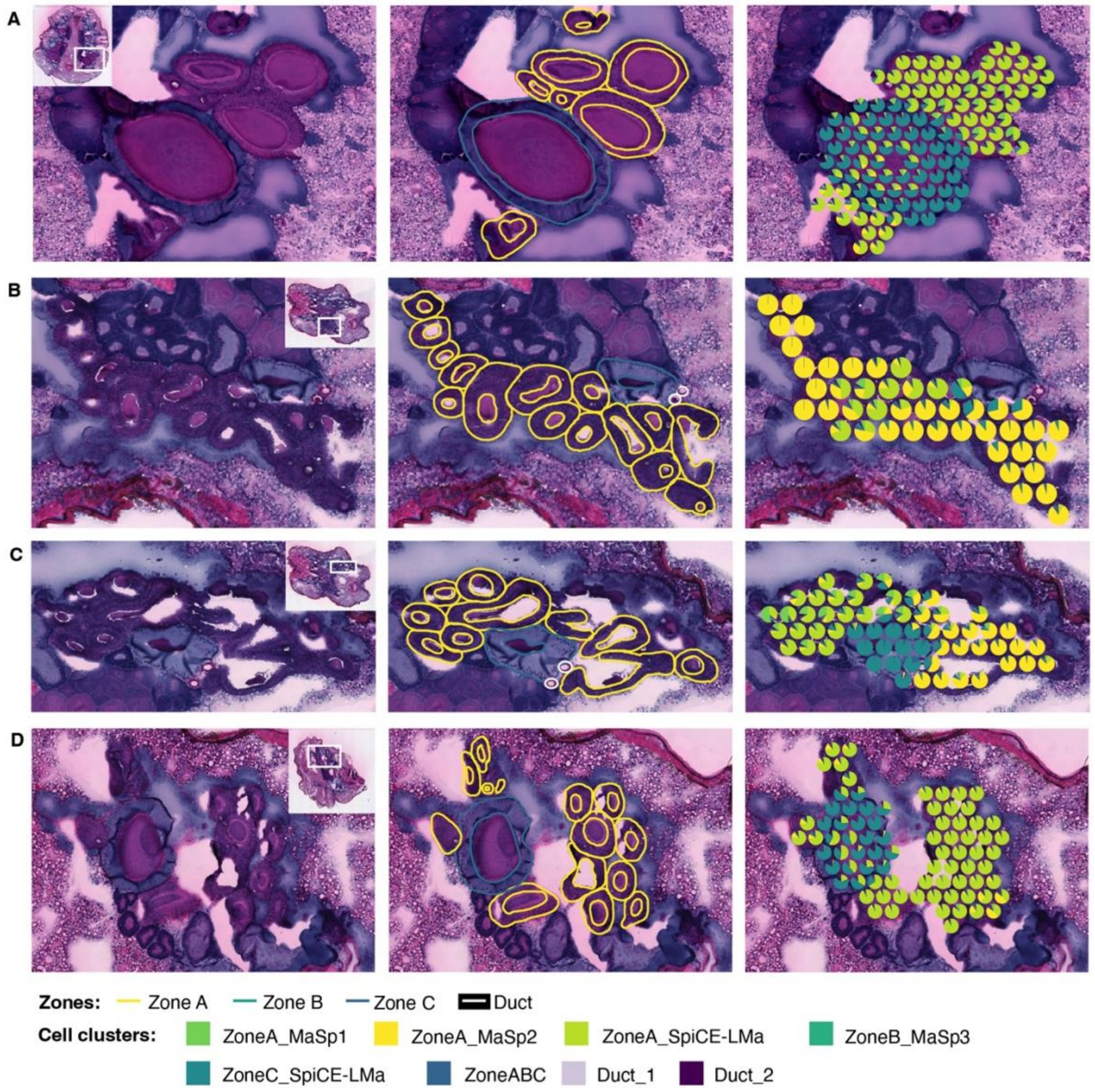

Fig. S36: Deconvolution plots for the major ampullate glands on spatial sections 1, 2, and 3.

(A) Major ampullate gland on section 1. **(B, C)** Major ampullate glands on section 2. **(D)** Major ampullate gland on section 3. In all, the left panels show HE-stained sections with inset showing the original images and rectangles indicating magnified regions. Middle panels show annotation of gland spots as zone A/B/C and duct with corresponding colors. Right panels show deconvolution of spatial spots into different major ampullate cell types identified from single-cell transcriptomic data, colors indicate cell types.

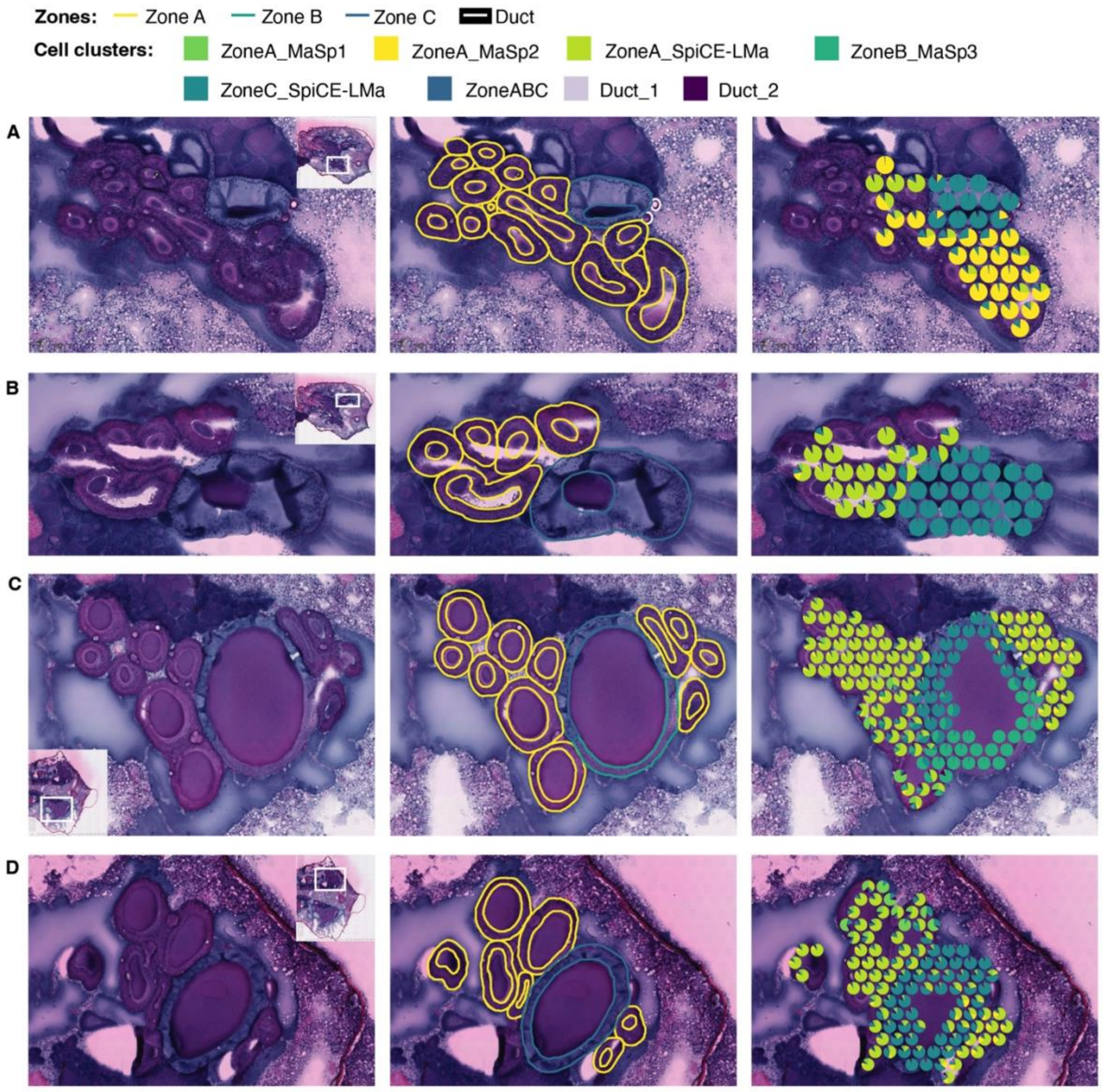

Fig. S37: Deconvolution plots for the major ampullate glands on spatial sections 5 and 6. (A, B) Major ampullate glands on section 5. **(C, D)** Major ampullate glands on section 6. In all, the left panels show HE-stained sections with inset showing the original images and rectangles indicating magnified regions. Middle panels show annotation of gland spots as zone A/B/C and duct with corresponding colors. Right panels show deconvolution of spatial spots into different cell types identified from single-cell transcriptomic data, colors indicate cell types.

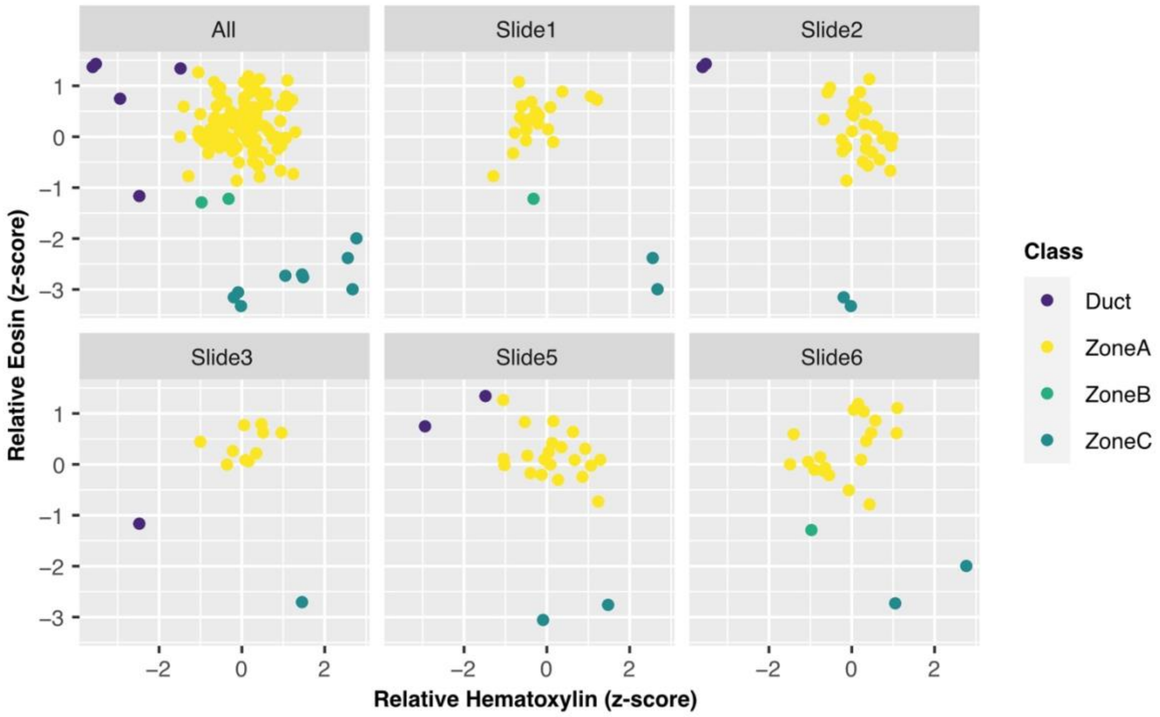

Fig. S38: Hematoxylin-Eosin plots for cross-sections of the different major ampullate zones from sections used for spatial transcriptomics.

The relative hematoxylin and eosin intensity values (z-scores) were generated for all the cross sections on different zones using QuPath. Each dot represents a cross-section annotated as zone A, zone B, zone C or duct.

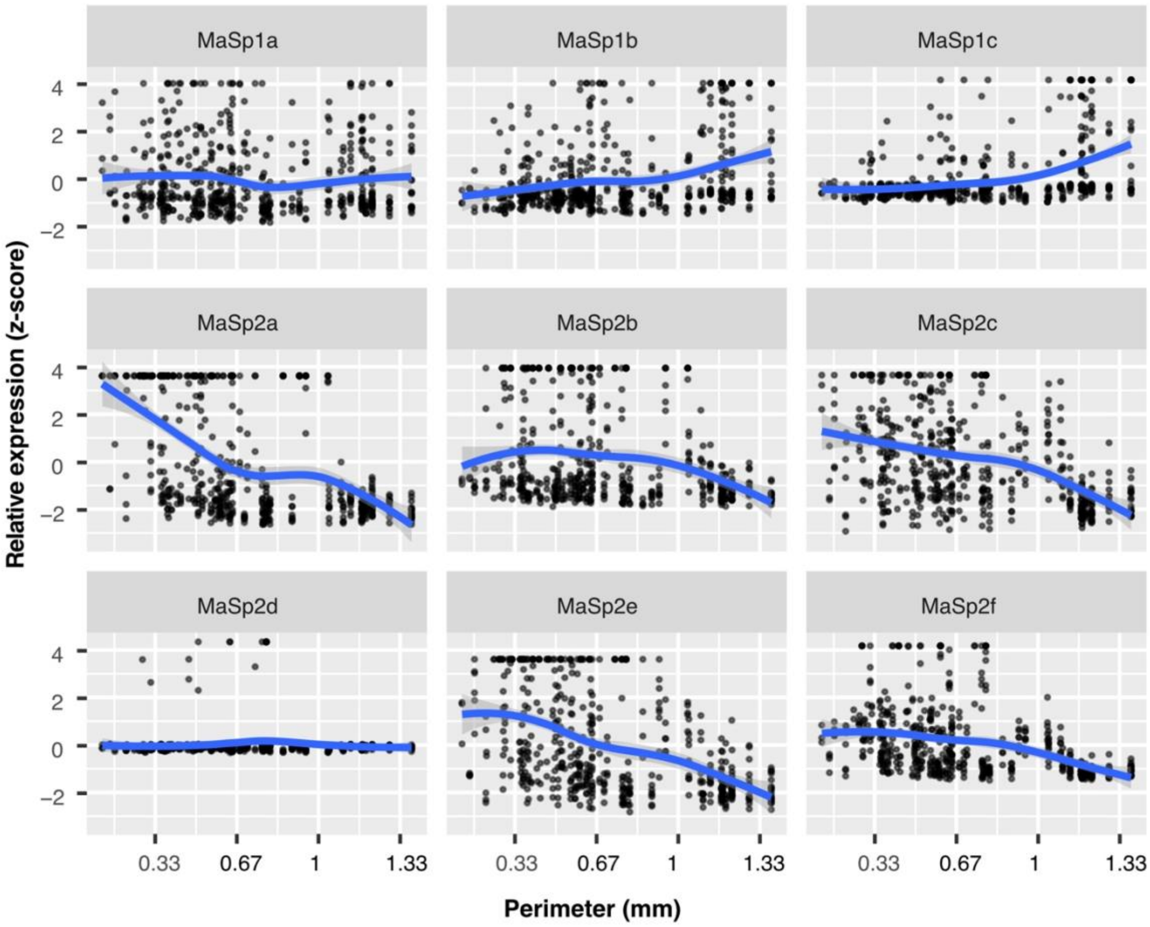

Fig. S39: Expression of *MaSp1* and *MaSp2* genes in zone A cross sections as a function of their perimeter.

Relative expression of *MaSp1 (a–c)* and *MaSp2 (a–f)* genes in the spots extracted from major ampullate zone A cross-sections. X-axis indicates the perimeter of the cross-section and y-axis indicates the relative expression level (z-scores) of the gene. Each dot represents a spot on the spatial section.

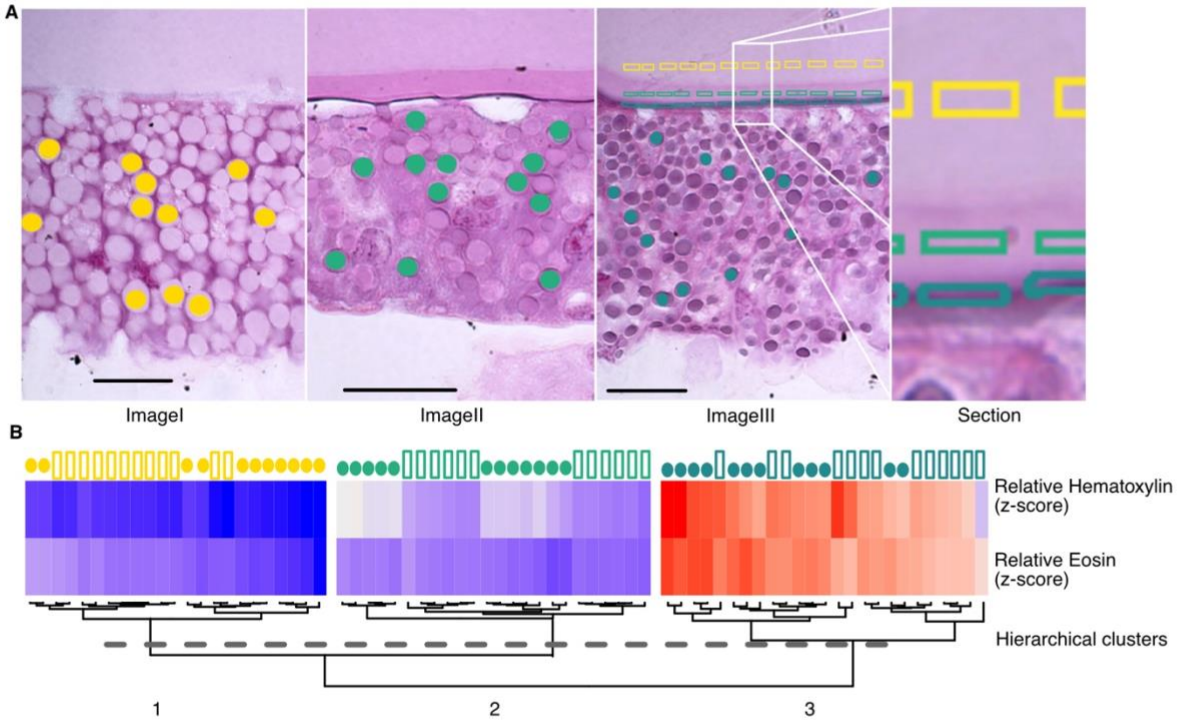

Fig. S40: QuPath analysis of the three zones of major ampullate gland.

(A) H&E-stained histological sections, indicated as Image I, II and II, show the morphology of the cells in the zone A, B and C, respectively, in the *L. sclopetarius* major ampullate gland. The round structures in ImageI–III (intracellular vesicles) and the rectangles in the three layers in the lumen shown in ImageIII indicate the annotated regions used for image analysis in QuPath. A small region of ImageIII is magnified in the last panel. Scale bar = 20 µm. **(B)** Heatmap showing the hematoxylin and eosin intensity values of the annotation objects shown in (A). The scale goes from blue (low values) to red (higher values). Colors above the heatmap correspond to the annotated regions in different zones: yellow (zone A), green (zone B) and dark green (zone C). Hierarchical clustering divides the annotated regions into three groups corresponding to the three zones.

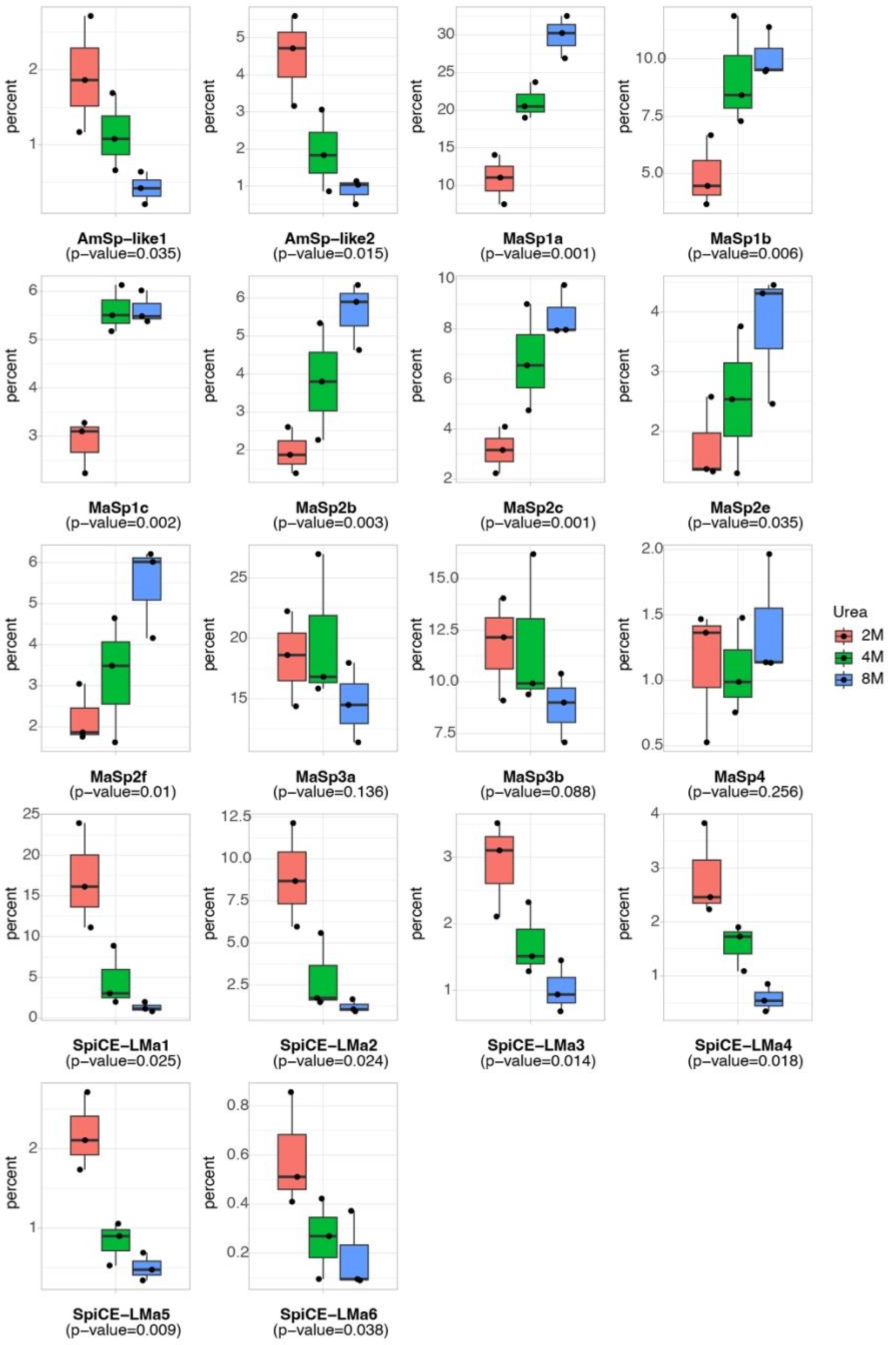

Fig. S41: Fraction of identified peptides from the 18 silk proteins in urea extracts from intact silk samples.

Fraction of peptides identified from spectral counts for the 18 silk proteins in the silk samples that were dissolved in different concentration of urea: 2M (yellow), 4M (green) and 8M (dark green). Proteins with p-values < 0.05 (when comparing levels between 2M and 8M urea samples) were considered as significantly enriched as shown in Fig. 7E.

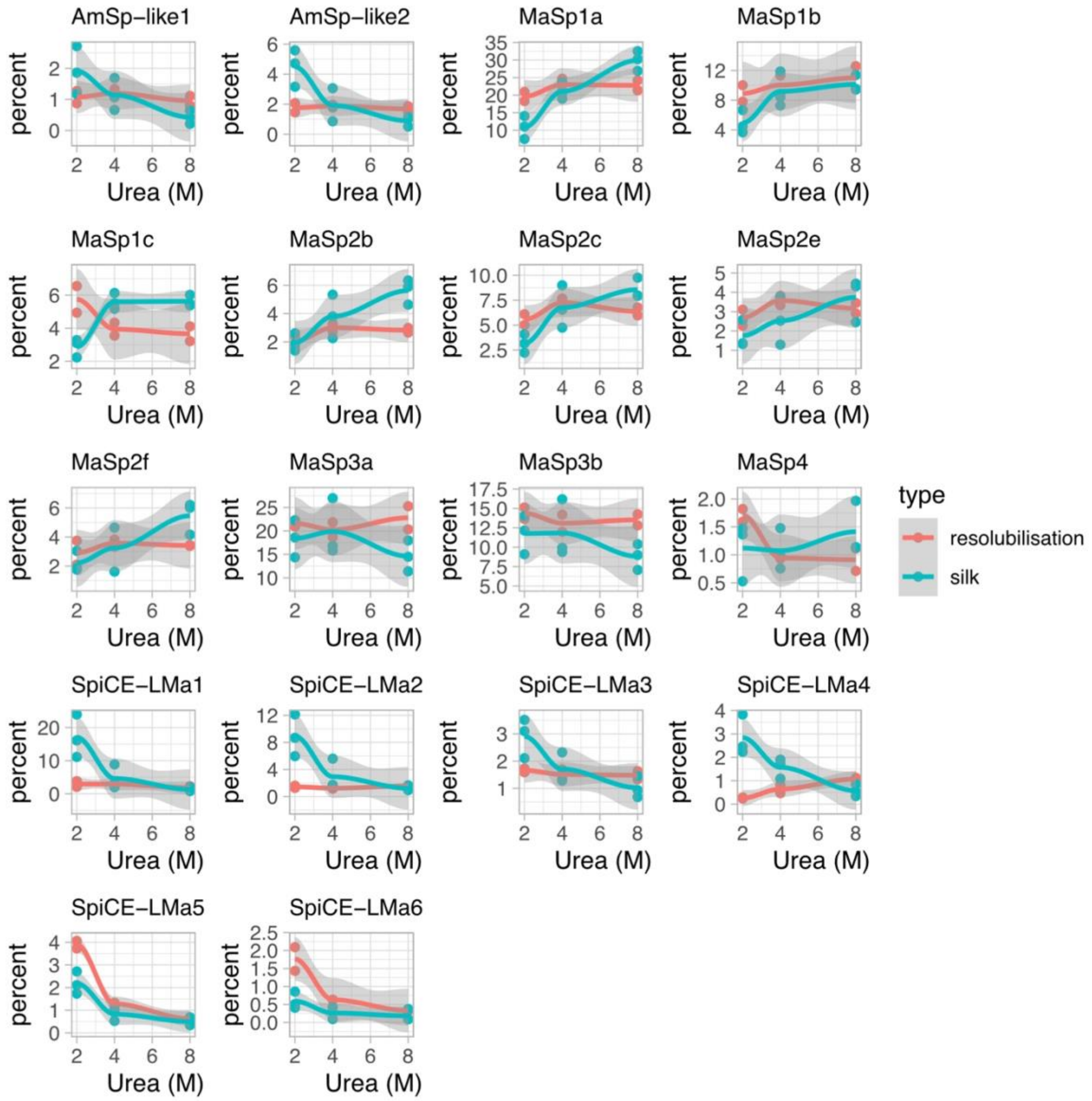

Fig. S42: Fraction of the peptides from 18 silk proteins in urea extracts from intact fibers and from dissolved fibers.

Plots show percent of peptides from the 18 silk proteins in supernatants from major ampullate silk fibers incubated in 2, 4 or 8M Urea (red). Green curves indicate the relative fraction of the peptides corresponding to different proteins in supernatants from major ampullate silk fibers that were first dissolved in formic acid, dried and then solubilized in 2, 4 or 8M urea, respectively.

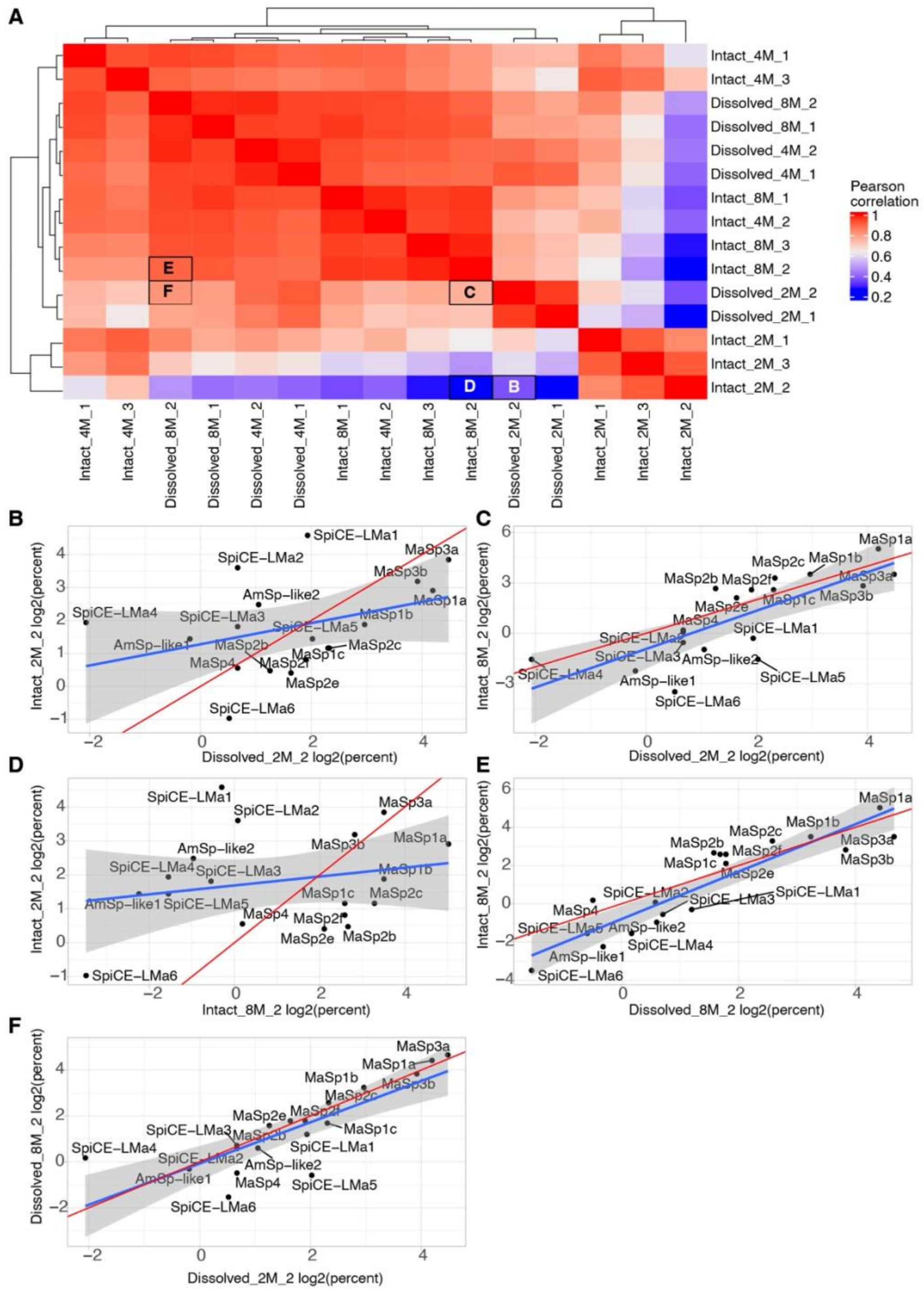

Fig. S43: Pearson correlation of intact and dissolved silk samples

Comparison of the relative protein abundance of the 18 silk proteins in urea extracts of the intact and dissolved samples of the silk. A total of 105 pairwise comparisons were conducted. Each comparison receives a Pearson r correlation value based on protein abundance similarity between the samples. (A) Hierarchical clustering of the Pearson correlation r-values between all samples. Red color indicates very similar protein abundance between the samples, while blue shows greater variation. The dendrogram shows the hierarchical cluster of the samples. (B) to (F) A subset of the pairwise comparisons highlighted in (A) as B, C, D, E, and F are presented as correlation plots. The blue line represents the linear model between the two samples, and the red line is a 1:1 ratio slope. Each spot represents one of the 18 silk proteins.

All proteins to the left of the red line are enriched in the sample named on the y-axis, and all points on the right side of the red line are enriched in the sample named on the X-axis. (B) Intact silk in 2M urea (y-axis) vs. dissolved silk in 2M urea (x-axis), Pearson correlation r = 0.368. (C) Intact silk in 8M urea (y-axis) vs. dissolved silk in 2M urea (x-axis), Pearson correlation r = 0.763***. (D) Intact silk in 8M urea (y-axis) vs. intact silk in 2M urea (x-axis), Pearson correlation r = 0.225. (E) Intact silk in 8M urea (y-axis) vs. intact silk in 8M urea (x-axis), Pearson correlation r = 0.907***. (F) Dissolved silk in 8M urea (y-axis) vs. dissolved silk in 2M urea (x-axis), Pearson correlation r = 0.785***. Three stars represent p-value < 0.001.

REFERENCES AND NOTES

1. A. Rising, M. J. Harrington, Biological materials processing: Time-tested tricks for sustainable fiber fabrication. *Chem. Rev.* **123**, 2155–2199 (2023).

2. K. Bourzac, Spiders: Web of intrigue. *Nature* **519**, S4–S6 (2015).

3. L. Brunetta, C. L. Craig, *Spider Silk: Evolution and 400 Million Years of Spinning, Waiting, Snagging, and Mating* (Yale Univ. Press, 2010).

4. D. B. Peakall, Synthesis of silk, mechanism and location. *Am. Zool.* **9**, 71–79 (1969).

5. F. Vollrath, Strength and structure of spiders' silks. *J. Biotechnol.* **74**, 67–83 (2000).

6. N. A. Ayoub, J. E. Garb, R. M. Tinghitella, M. A. Collin, C. Y. Hayashi, Blueprint for a high-performance biomaterial: Full-length spider dragline silk genes. *PLOS ONE* **2**, e514 (2007).

7. P. L. Babb, N. F. Lahens, S. M. Correa-Garhwal, D. N. Nicholson, E. J. Kim, J. B. Hogenesch, M. Kuntner, L. Higgins, C. Y. Hayashi, I. Agnarsson, B. F. Voight, The *Nephila clavipes* genome highlights the diversity of spider silk genes and their complex expression. *Nat. Genet.* **49**, 895–903 (2017).

8. N. Kono, H. Nakamura, R. Ohtoshi, D. A. P. Moran, A. Shinohara, Y. Yoshida, M. Fujiwara, M. Mori, M. Tomita, K. Arakawa, Orb-weaving spider *Araneus ventricosus* genome elucidates the spidroin gene catalogue. *Sci. Rep.* **9**, 8380 (2019).

9. N. Kono, H. Nakamura, M. Mori, Y. Yoshida, R. Ohtoshi, A. D. Malay, D. A. Pedrazzoli Moran, M. Tomita, K. Numata, K. Arakawa, Multicomponent nature underlies the extraordinary mechanical properties of spider dragline silk. *Proc. Natl. Acad. Sci. U.S.A.* **118**, e2107065118 (2021).

10. N. Kono, R. Ohtoshi, A. D. Malay, M. Mori, H. Masunaga, Y. Yoshida, H. Nakamura, K. Numata, K. Arakawa, Darwin's bark spider shares a spidroin repertoire with *Caerostris extrusa* but achieves extraordinary silk toughness through gene expression. *Open Biol.* **11**, 210242 (2021).

11. P. L. Babb, M. Gregoric, N. F. Lahens, D. N. Nicholson, C. Y. Hayashi, L. Higgins, M. Kuntner, I. Agnarsson, B. F. Voight, Characterization of the genome and silk-gland transcriptomes of Darwin's bark spider (*Caerostris darwini*). *PLOS ONE* **17**, e0268660 (2022).

12. K. Arakawa, N. Kono, A. D. Malay, A. Tateishi, N. Ifuku, H. Masunaga, R. Sato, K. Tsuchiya, R. Ohtoshi, D. Pedrazzoli, A. Shinohara, Y. Ito, H. Nakamura, A. Tanikawa, Y. Suzuki, T. Ichikawa, S. Fujita, M. Fujiwara, M. Tomita, S. J. Blamires, J.-A. Chuah, H. Craig, C. P. Foong, G. Greco, J. Guan, C. Holland, D. L. Kaplan, K. Sudesh, B. B. Mandal, Y Norma-Rashid, N. A. Oktaviani, R. C. Preda, N. M. Pugno, R. Rajkhowa, X. Wang, K. Yazawa, Z. Zheng, K. Numata, 1000 spider silkomes: Linking sequences to silk physical properties. *Sci. Adv.* **8**, eabo6043 (2022).

13. W. Hu, A. Jia, S. Ma, G. Zhang, Z. Wei, F. Lu, Y. Luo, Z. Zhang, J. Sun, T. Yang, T. Xia, Q. Li, T. Yao, J. Zheng, Z. Jiang, Z. Xu, Q. Xia, Y. Wang, A molecular atlas reveals the tri-sectional spinning mechanism of spider dragline silk. *Nat. Commun.* **14**, 837 (2023).

14. J. E. Garb, N. A. Ayoub, C. Y. Hayashi, Untangling spider silk evolution with spidroin terminal domains. *BMC Evol. Biol.* **10**, 243 (2010).

15. M. Xu, R. V. Lewis, Structure of a protein superfiber: Spider dragline silk. *Proc. Natl. Acad. Sci. U.S.A.* **87**, 7120–7124 (1990).

16. M. B. Hinman, R. V. Lewis, Isolation of a clone encoding a second dragline silk fibroin. *Nephila clavipes* dragline silk is a two-protein fiber. *J. Biol. Chem.* **267**, 19320–19324 (1992).

17. M. A. Collin, T. H. Clarke III, N. A. Ayoub, C. Y. Hayashi, Genomic perspectives of spider silk genes through target capture sequencing: Conservation of stabilization mechanisms and homology-based structural models of spidroin terminal regions. *Int. J. Biol. Macromol.* **113**, 829–840 (2018).

18. J. E. Garb, R. A. Haney, E. E. Schwager, M. Gregoric, M. Kuntner, I. Agnarsson, T. A. Blackledge, The transcriptome of Darwin's bark spider silk glands predicts proteins contributing to dragline silk toughness. *Commun. Biol.* **2**, 275 (2019).

19. M. Saric, L. Eisoldt, V. Doring, T. Scheibel, Interplay of different major ampullate spidroins during assembly and implications for fiber mechanics. *Adv. Mater.* **33**, e2006499 (2021).

20. C. Y. Hayashi, R. V. Lewis, Evidence from flagelliform silk cDNA for the structural basis of elasticity and modular nature of spider silks. *J. Mol. Biol.* **275**, 773–784 (1998).

21. K. W. Sanggaard, J. S. Bechsgaard, X. Fang, J. Duan, T. F. Dyrlund, V. Gupta, X. Jiang, L. Cheng, D. Fan, Y. Feng, L. Han, Z. Huang, Z. Wu, L. Liao, V. Settepani, I. B. Thogersen, B. Vanthournout, T. Wang, Y. Zhu, J. Wang, Spider genomes provide insight into composition and evolution of venom and silk. *Nat. Commun.* **5**, 3765 (2014).

22. C. Larracas, R. Hekman, S. Dyrness, A. Arata, C. Williams, T. Crawford, C. A. Vierra, Comprehensive proteomic analysis of spider dragline silk from black widows: A recipe to build synthetic silk fibers. *Int. J. Mol. Sci.* **17**, 1–16 (2016).

23. T. Pham, T. Chuang, A. Lin, H. Joo, J. Tsai, T. Crawford, L. Zhao, C. Williams, Y. Hsia, C. Vierra, Dragline silk: A fiber assembled with low-molecular-weight cysteine-rich proteins. *Biomacromolecules* **15**, 4073–4081 (2014).

24. J. Kovoor, Comparative structure and histochemistry of silk-producing organs in arachnids, in *Ecophysiology of Spiders*, W. Nentwig, Ed. (Springer, 1987), pp. 160–186.

25. F. Vollrath, D. P. Knight, Structure and function of the silk production pathway in the spider *Nephila edulis*. *Int. J. Biol. Macromol.* **24**, 243–249 (1999).

26. M. Andersson, L. Holm, Y. Ridderstrale, J. Johansson, A. Rising, Morphology and composition of the spider major ampullate gland and dragline silk. *Biomacromolecules* **14**, 2945–2952 (2013).

27. S. F. Li, A. J. McGhie, S. L. Tang, New internal structure of spider dragline silk revealed by atomic force microscopy. *Biophys. J.* **66**, 1209–1212 (1994).

28. F. Vollrath, T. Holtet, H. C. Thøgersen, S. Frische, Structural organization of spider silk. *R. Soc. Lond.* **263**, 147–151 (1996).

29. S. Frische, A. B. Maunsbach, F. Vollrath, Elongate cavities and skin-core structure in *Nephila* spider silk observed by electron microscopy. *J. Microsc.* **189**, 64–70 (1998).

30. K. Augsten, P. Muhlig, C. Herrmann, Glycoproteins and skin-core structure in *Nephila clavipes* spider silk observed by light and electron microscopy. *Scanning* **22**, 12–15 (2000).

31. P. Poza, J. Pérez-Rigueiro, M. Elices, J. Llorca, Fractographic analysis of silkworm and spider silk. *Eng. Fract. Mech.* **69**, 1035–1048 (2002).

32. A. Sponner, W. Vater, S. Monajembashi, E. Unger, F. Grosse, K. Weisshart, Composition and hierarchical organisation of a spider silk. *PLOS ONE* **2**, e998 (2007).

33. I. Iachina, J. Fiutowski, H. G. Rubahn, F. Vollrath, J. R. Brewer, Nanoscale imaging of major and minor ampullate silk from the orb-web spider *Nephila madagascariensis*. *Sci. Rep.* **13**, 6695 (2023).

34. A. Sponner, E. Unger, F. Grosse, K. Weisshart, Differential polymerization of the two main protein components of dragline silk during fibre spinning. *Nat. Mater.* **4**, 772–775 (2005).

35. D. H. Hijirida, K. G. Do, C. Michal, S. Wong, D. Zax, L. W. Jelinski, 13C NMR of *Nephila clavipes* major ampullate silk gland. *Biophys. J.* **71**, 3442–3447 (1996).

36. M. Andersson, G. Chen, M. Otikovs, M. Landreh, K. Nordling, N. Kronqvist, P. Westermark, H. Jörnvall, S. Knight, Y. Ridderstråle, L. Holm, Q. Meng, K. Jaudzems, M. Chesler, J. Johansson, A. Rising, Carbonic anhydrase generates CO_2 and H^+ that drive spider silk formation via opposite effects on the terminal domains. *PLOS Biol.* **12**, e1001921 (2014).

37. F. Hagn, L. Eisoldt, J. G. Hardy, C. Vendrely, M. Coles, T. Scheibel, H. Kessler, A conserved spider silk domain acts as a molecular switch that controls fibre assembly. *Nature* **465**, 239–242 (2010).

38. N. Kronqvist, M. Otikovs, V. Chmyrov, G. Chen, M. Andersson, K. Nordling, M. Landreh, M. Sarr, H. Jornvall, S. Wennmalm, J. Widengren, Q. Meng, A. Rising, D. Otzen, S. D. Knight, K.

Jaudzems, J. Johansson, Sequential pH-driven dimerization and stabilization of the N-terminal domain enables rapid spider silk formation. *Nat. Commun.* **5**, 3254 (2014).

39. M. Landreh, G. Askarieh, K. Nordling, M. Hedhammar, A. Rising, C. Casals, J. Astorga-Wells, G. Alvelius, S. D. Knight, J. Johansson, H. Jörnvall, T. Bergman, A pH-dependent dimer lock in spider silk protein. *J. Mol. Biol.* **404**, 328–336 (2010).

40. A. Rising, J. Johansson, Toward spinning artificial spider silk. *Nat. Chem. Biol.* **11**, 309–315 (2015).

41. J. Sparkes, C. Holland, Analysis of the pressure requirements for silk spinning reveals a pultrusion dominated process. *Nat. Commun.* **8**, 594 (2017).

42. J. Jumper, R. Evans, A. Pritzel, T. Green, M. Figurnov, O. Ronneberger, K. Tunyasuvunakool, R. Bates, A. Žídek, A. Potapenko, A. Bridgland, C. Meyer, S. A. A. Kohl, A. J. Ballard, A. Cowie, B. Romera-Paredes, S. Nikolov, R. Jain, J. Adler, T. Back, S. Petersen, D. Reiman, E. Clancy, M. Zielinski, M. Steinegger, M. Pacholska, T. Berghammer, S. Bodenstein, D. Silver, O. Vinyals, A. W. Senior, K. Kavukcuoglu, P. Kohli, D. Hassabis, Highly accurate protein structure prediction with AlphaFold. *Nature* **596**, 583–589 (2021).

43. M. Varadi, S. Anyango, M. Deshpande, S. Nair, C. Natassia, G. Yordanova, D. Yuan, O. Stroe, G. Wood, A. Laydon, A. Zidek, T. Green, K. Tunyasuvunakool, S. Petersen, J. Jumper, E. Clancy, R. Green, A. Vora, M. Lutfi, S. Velankar, AlphaFold protein structure database: Massively expanding the structural coverage of protein-sequence space with high-accuracy models. *Nucleic Acids Res.* **50**, D439-D444 (2022).

44. P. L. Ståhl, F. Salmén, S. Vickovic, A. Lundmark, J. F. Navarro, J. Magnusson, S. Giacomello, M. Asp, J. O. Westholm, M. Huss, A. Mollbrink, S. Linnarsson, S. Codeluppi, Å. Borg, F. Pontén, P. I. Costea, P. Sahlén, J. Mulder, O. Bergmann, J. Lundeberg, J. Frisén, Visualization and analysis of gene expression in tissue sections by spatial transcriptomics. *Science* **353**, 78–82 (2016).

45. L. McInnes, J. Healy, J. Melville, Umap: Uniform manifold approximation and projection for dimension reduction. arXiv:1802.03426 [stat.ML] (2018).

46. P. Bankhead, M. B. Loughrey, J. A. Fernandez, Y. Dombrowski, D. G. McArt, P. D. Dunne, S. McQuaid, R. T. Gray, L. J. Murray, H. G. Coleman, J. A. James, M. Salto-Tellez, P. W. Hamilton, QuPath: Open source software for digital pathology image analysis. *Sci. Rep.* **7**, 16878 (2017).

47. R. C. Chaw, S. M. Correa-Garhwal, T. H. Clarke, N. A. Ayoub, C. Y. Hayashi, Proteomic evidence for components of spider silk synthesis from black widow silk glands and fibers. *J. Proteome Res.* **14**, 4223–4231 (2015).

48. K. Yazawa, A. D. Malay, H. Masunaga, K. Numata, Role of skin layers on mechanical properties and supercontraction of spider dragline silk fiber. *Macromol. Biosci.* **19**, 1800220 (2019).

49. B. Madsen, Z. Z. Shao, F. Vollrath, Variability in the mechanical properties of spider silks on three levels: Interspecific, intraspecific and intraindividual. *Int. J. Biol. Macromol.* **24**, 301–306 (1999).

50. S. Sonavane, P. Westermark, A. Rising, L. Holm, Regionalization of cell types in silk glands of *Larinioides sclopetarius* suggest that spider silk fibers are complex layered structures. *Sci. Rep.* **13**, 22273 (2023).

51. K. Jaudzems, G. Askarieh, M. Landreh, K. Nordling, M. Hedhammar, H. Jornvall, A. Rising, S. D. Knight, J. Johansson, pH-dependent dimerization of spider silk N-terminal domain requires relocation of a wedged tryptophan side chain. *J. Mol. Biol.* **422**, 477–487 (2012).

52. S. Keten, M. J. Buehler, Atomistic model of the spider silk nanostructure. *Appl. Phys. Lett.* **96**, 153701 (2010).

53. W. Lu, D. L. Kaplan, M. J. Buehler, Generative modeling, design, and analysis of spider silk protein sequences for enhanced mechanical properties. *Adv. Funct. Mater.* **34**, 2311324 (2024).

54. C. S. Chin, P. Peluso, F. J. Sedlazeck, M. Nattestad, G. T. Concepcion, A. Clum, C. Dunn, R. O'Malley, R. Figueroa-Balderas, A. Morales-Cruz, G. R. Cramer, M. Delledonne, C. Luo, J. R. Ecker, D. Cantu, D. R. Rank, M. C. Schatz, Phased diploid genome assembly with single-molecule real-time sequencing. *Nat. Methods* **13**, 1050–1054 (2016).

55. B. J. Walker, T. Abeel, T. Shea, M. Priest, A. Abouelliel, S. Sakthikumar, C. A. Cuomo, Q. Zeng, J. Wortman, S. K. Young, A. M. Earl, Pilon: An integrated tool for comprehensive microbial variant detection and genome assembly improvement. *PLOS ONE* **9**, e112963 (2014).

56. A. M. Bolger, M. Lohse, B. Usadel, Trimmomatic: A flexible trimmer for Illumina sequence data. *Bioinformatics* **30**, 2114–2120 (2014).

57. G. Marcais, C. Kingsford, A fast, lock-free approach for efficient parallel counting of occurrences of k-mers. *Bioinformatics* **27**, 764–770 (2011).

58. T. R. Ranallo-Benavidez, K. S. Jaron, M. C. Schatz, GenomeScope 2.0 and Smudgeplot for reference-free profiling of polyploid genomes. *Nat. Commun.* **11**, 1432 (2020).

59. A. R. Quinlan, I. M. Hall, BEDTools: A flexible suite of utilities for comparing genomic features. *Bioinformatics* **26**, 841–842 (2010).

60. M. Bernt, A. Donath, F. Juhling, F. Externbrink, C. Florentz, G. Fritzsch, J. Putz, M. Middendorf, P. F. Stadler, MITOS: Improved de novo metazoan mitochondrial genome annotation. *Mol. Phylogenet. Evol.* **69**, 313–319 (2013).

61. H. Li, Minimap2: Pairwise alignment for nucleotide sequences. *Bioinformatics* **34**, 3094–3100 (2018).

62. F. A. Simao, R. M. Waterhouse, P. Ioannidis, E. V. Kriventseva, E. M. Zdobnov, BUSCO: Assessing genome assembly and annotation completeness with single-copy orthologs. *Bioinformatics* **31**, 3210–3212 (2015).

63. B. L. Cantarel, I. Korf, S. M. Robb, G. Parra, E. Ross, B. Moore, C. Holt, A. Sanchez Alvarado, M. Yandell, MAKER: An easy-to-use annotation pipeline designed for emerging model organism genomes. *Genome Res.* **18**, 188–196 (2008).

64. T. M. Lowe, S. R. Eddy, tRNAscan-SE: A program for improved detection of transfer RNA genes in genomic sequence. *Nucleic Acids Res.* **25**, 955–964 (1997).

65. E. P. Nawrocki, D. L. Kolbe, S. R. Eddy, Infernal 1.0: Inference of RNA alignments. *Bioinformatics* **25**, 1335–1337 (2009).

66. S. W. Burge, J. Daub, R. Eberhardt, J. Tate, L. Barquist, E. P. Nawrocki, S. R. Eddy, P. P. Gardner, A. Bateman, Rfam 11.0: 10 years of RNA families. *Nucleic Acids Res.* **41**, D226–D232 (2013).

67. M. Stanke, R. Steinkamp, S. Waack, B. Morgenstern, AUGUSTUS: A web server for gene finding in eukaryotes. *Nucleic Acids Res.* **32**, W309–W312 (2004).

68. I. Korf, Gene finding in novel genomes. *BMC Bioinformatics* **5**, 59 (2004).

69. T. Paysan-Lafosse, M. Blum, S. Chuguransky, T. Grego, B. L. Pinto, G. A. Salazar, M. L. Bileschi, P. Bork, A. Bridge, L. Colwell, J. Gough, D. H. Haft, I. Letunić, A. Marchler-Bauer, H. Mi, D. A. Natale, C. A. Orengo, A. P. Pandurangan, C. Rivoire, C. J. A. Sigrist, I. Sillitoe, N. Thanki, P. D. Thomas, S. C. E. Tosatto, C. H. Wu, A. Bateman, InterPro in 2022. *Nucleic Acids Res.* **51**, D418–D427 (2023).

70. T. Smith, A. Heger, I. Sudbery, UMI-tools: Modeling sequencing errors in unique molecular identifiers to improve quantification accuracy. *Genome Res.* **27**, 491–499 (2017).

71. E. Lee, G. A. Helt, J. T. Reese, M. C. Munoz-Torres, C. P. Childers, R. M. Buels, L. Stein, I. H. Holmes, C. G. Elsik, S. E. Lewis, Web Apollo: A web-based genomic annotation editing platform. *Genome Biol.* **14**, R93 (2013).

72. F. Teufel, J. J. A. Armenteros, A. R. Johansen, M. H. Gíslason, S. I. Pihl, K. D. Tsirigos, O. Winther, S. Brunak, G. von Heijne, H. Nielsen, SignalP 6.0 predicts all five types of signal peptides using protein language models. *Nat. Biotechnol.* **40**, 1023–1025 (2022).

73. K. Okonechnikov, O. Golosova, M. Fursov; UGENE team, Unipro UGENE: A unified bioinformatics toolkit. *Bioinformatics* **28**, 1166–1167 (2012).

74. E. Birney, M. Clamp, R. Durbin, GeneWise and genomewise. *Genome Res.* **14**, 988–995 (2004).

75. O. Keller, F. Odronitz, M. Stanke, M. Kollmar, S. Waack, Scipio: Using protein sequences to determine the precise exon/intron structures of genes and their orthologs in closely related species. *BMC Bioinformatics* **9**, 278 (2008).

76. D. M. Emms, S. Kelly, OrthoFinder: Solving fundamental biases in whole genome comparisons dramatically improves orthogroup inference accuracy. *Genome Biol.* **16**, 157 (2015).

77. A. Dobin, C. A. Davis, F. Schlesinger, J. Drenkow, C. Zaleski, S. Jha, P. Batut, M. Chaisson, T. R. Gingeras, STAR: Ultrafast universal RNA-seq aligner. *Bioinformatics* **29**, 15–21 (2013).

78. Y. Liao, G. K. Smyth, W. Shi, FeatureCounts: An efficient general purpose program for assigning sequence reads to genomic features. *Bioinformatics* **30**, 923–930 (2014).

79. Y. Liao, G. K. Smyth, W. Shi, The R package Rsubread is easier, faster, cheaper and better for alignment and quantification of RNA sequencing reads. *Nucleic Acids Res.* **47**, e47 (2019).

80. M. I. Love, W. Huber, S. Anders, Moderated estimation of fold change and dispersion for RNA-seq data with DESeq2. *Genome Biol.* **15**, 550 (2014).

81. E. A. Thévenot, A. Roux, Y. Xu, E. Ezan, C. Junot, Analysis of the human adult urinary metabolome variations with age, body mass index, and gender by implementing a comprehensive workflow for univariate and OPLS statistical analyses. *J. Proteome Res.* **14**, 3322–3335 (2015).

82. R. Satija, J. A. Farrell, D. Gennert, A. F. Schier, A. Regev, Spatial reconstruction of single-cell gene expression data. *Nat. Biotechnol.* **33**, 495–502 (2015).

83. Y. Hao, S. Hao, E. Andersen-Nissen, W. M. Mauck III, S. Zheng, A. Butler, M. J. Lee, A. J. Wilk, C. Darby, M. Zager, P. Hoffman, M. Stoeckius, E. Papalexi, E. P. Mimitou, J. Jain, A. Srivastava, T. Stuart, L. M. Fleming, B. Yeung, R. Satija, Integrated analysis of multimodal single-cell data. *Cell* **184**, 3573–3587.e29 (2021).

84. Y. Ma, X. Zhou, Spatially informed cell-type deconvolution for spatial transcriptomics. *Nat. Biotechnol.* **40**, 1349–1359 (2022).

85. R. Foelix, *Biology of Spiders* (Oxford Univ. Press, 1996).

86. Y. Perez-Riverol, J. Bai, C. Bandla, D. Garcia-Seisdedos, S. Hewapathirana, S. Kamatchinathan, D. J. Kundu, A. Prakash, A. Frericks-Zipper, M. Eisenacher, M. Walzer, S. Wang, A. Brazma, J. A. Vizcaino, The PRIDE database resources in 2022: A hub for mass spectrometry-based proteomics evidences. *Nucleic Acids Res.* **50**, D543–D552 (2022).

87. R. Challis, E. Richards, J. Rajan, G. Cochrane, M. Blaxter, BlobToolKit—Interactive quality assessment of genome assemblies *Genetics* **10**, 1361–1374 (2020).

88. T. H. Clarke, J. E. Garb, R. A. Haney, R. C. Chaw, C. Y. Hayashi, N. A. Ayoub, Evolutionary shifts in gene expression decoupled from gene duplication across functionally distinct spider silk glands. *Sci. Rep.* **7**, 8393 (2017).

89. L. J. McGuffin, K. Bryson, D. T. Jones, The PSIPRED protein structure prediction server. *Bioinformatics* **16**, 404–405 (2000).
